# One‐Dimensional Polycyclic Aromatic Hydrocarbons Incorporating Multiple Dithiafulvene Units—Novel Multi‐Redox and Electrochromic Systems

**DOI:** 10.1002/anie.202525025

**Published:** 2026-03-05

**Authors:** Cecilie Rindom, Florim Seljmani, Lukas Bradley Woodcock, Mogens Brøndsted Nielsen

**Affiliations:** ^1^ Department of Chemistry University of Copenhagen Copenhagen Ø Denmark

**Keywords:** conjugation, intermolecular association, polycycles, redox chemistry, tetrathiafulvalene

## Abstract

The combination of polycyclic aromatic hydrocarbons (PAHs) and dithiafulvene (DTF) units provides large π‐conjugated scaffolds with multi‐redox behavior. Here, we present the synthesis and properties of PAHs containing seven or nine alternating and fused six‐ and five‐membered rings and three or four DTF units located along the PAHs, tri‐DTF and tetra‐DTF PAH scaffolds. The synthesis relied on the generation of PAH cores with carbonyl groups at the five‐membered rings to allow for Horner–Wadsworth–Emmons reactions to introduce the DTF units. These planar molecules exhibited very strong associations in neutral and oxidized states, and the various redox states had distinct UV–vis–NIR absorptions. Proceeding from an odd number to an even number of DTF units had a remarkable consequence for the redox properties. Thus, cyclic voltammetry revealed the tri‐DTF PAH to undergo stepwise one‐electron oxidations to form the trication with a broadened first oxidation wave owing to associations. Instead, the radical cation of the tetra‐DTF PAH underwent a single three‐electron oxidation, signaling an exceptional driving force for generating the tetracation. By electrocrystallization both mixed‐valence neutral–radical cation and radical cation salts were generated. These multi‐DTF PAHs are particularly interesting as potential components for electrochromic and conducting materials or as redox‐controllable tectons for molecular self‐/disassembly.

## Graphical abstract

Planar polycyclic aromatic hydrocarbons (PAHs) incorporating three or four dithiafulvene (DTF) units were synthesized via Horner–Wadsworth–Emmons reactions to create large π‐conjugated redox‐active frameworks. These molecules display strong intermolecular associations and distinct odd–even redox behavior, enabling formation of mixed‐valence and radical‐cation salts by electrocrystallization with characteristic strong NIR absorptions.

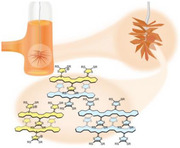

## 1 Introduction

The drive toward smaller and more efficient electronic components has placed molecular electronics at the forefront of materials research. Individual molecules can function as active elements such as wires, diodes, switches, or memory units [1, 2, 3]. Conventional inorganic materials hit limits in miniaturization, whereas tailored π‐conjugated organic molecules offer modularity, tunable redox behavior, and the possibility of self‐assembly into ordered architectures [4, 5]. Among the wide palette of candidate molecular systems, tetrathiafulvalene (TTF) and its derivatives have held a privileged status [6, 7, 8].

**FIGURE 1:**
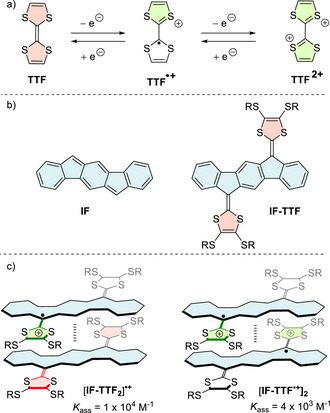
(a) Structure of TTF and its redox properties. (b) Structure of **IF** (left) and **IF‐TTF** (right). (c) Schematic illustration of mixed‐valence (left) and π‐dimer complexes (right) of oxidized **IF‐TTF** in CH_2_Cl_2_ [29].

TTF undergoes two reversible, stepwise oxidations (Figure 1a) while maintaining structural integrity and stability at ambient conditions [9, 10]. This redox “switchability” enables the design of functional materials in which conductivity, charge distribution, or conformation can be modulated electrochemically or by interaction with electron acceptors [11, 12]. Indeed, the charge‐transfer salt with tetracyanoquinodimethane (TCNQ) was shown in the 1970's to exhibit metallic conductivity at low temperature [13, 14, 15]. Over decades, TTF‐based systems have been incorporated into redox‐responsive molecular wires, donor–acceptor assemblies, molecular switches, and supramolecular architectures [16, 17, 18, 19, 20, 21]. TTF is known to form mixed‐valence complexes between a neutral and radical cation species or π‐dimer complexes between two radical cation species [22]. These complexes are, however, very weak in solution, but can be enhanced in mechanically interlocked systems or in cage structures [23, 24, 25]. Extension of TTF into larger π‐systems by separating the two dithiole rings by a spacer allows for modulation of not only optical and electronic properties but also in some cases the ability to associate [26, 27, 28, 29, 30, 31].

The indeno[1,2‐*b*]fluorene (IF)‐extended TTF motif has attracted particular interest (Figure 1b) for redox‐controlled assembly/disassembly and for exhibiting distinct UV–vis–NIR optical properties of its various redox states (0, +1, +2) [29, 30, 31]. Thus, self‐association complexes of the parent **IF‐TTF** (R = Bu, Figure 1c) exhibit a four‐orders of magnitude increase in association constants for both mixed‐valence and π‐dimers compared to those of the parent TTF [29]. In addition, electrocrystallization has provided cation salts showing semi‐conducting properties. These salts were either composed of radical cations or the mixed‐valence radical cation/dication combination, though it never turned possible to obtain the combination of neutral and radical cation that would be particularly interesting for intervalence charge transport [32, 33, 34, 35]. Various approaches for achieving this mixed‐valence state in the solid state have been attempted, for example, by covalently linking two IF‐TTF units, but without success [36].

**FIGURE 2:**
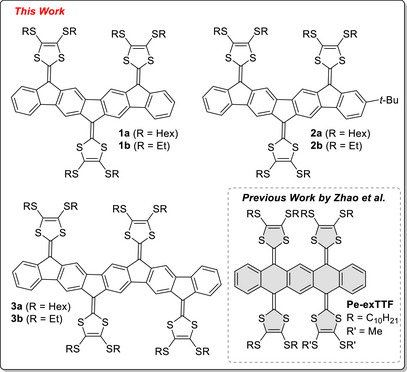
Extended multi‐DTF systems **1**, **2**, and **3** presented in this work and previously published pentacene‐extended TTF (**Pe‐exTTF**) analogue reported by Zhao and co‐workers [37].

By further extending the length of a polycyclic aromatic hydrocarbon (PAH) core, more than two dithiafulvene (DTF) units can be introduced. Zhao and co‐workers [37] reported in 2015 the synthesis of a pentacene‐extended TTF analogue, embedding four DTF units along the same backbone (**Pe‐exTTF**, Figure 2). Electrochemical studies revealed two reversible two‐electron processes, thereby resembling the two‐electron oxidation of the well‐known anthracene‐extended TTF [38]. In the neutral state, the pentacene‐TTF analogue showed a bent geometry that upon oxidation to the tetracation underwent a dramatic conformational change to a planar pentacene structure with four 1,3‐dithiolium appendages. Other multi‐DTF systems have been reported, for example, with acetylenic/phenyleneethynylene, olefinic (cross‐conjugated), or truxene cores [26, 27, 39, 40, 41].

In this work, aiming for planar structures and access to the radical cation, we present the design, synthesis, and investigation of a new class of multi‐DTF systems, separated by a π‐linker based on the IF‐scaffold—specifically compounds **1a**/**b**, **2a**/**b**, and **3a**/**b** (Figure 2). These systems incorporate three or four DTF units along one‐dimensional IF‐based PAH cores that span seven or nine fused rings. This architecture enables systematic investigation of how an increasing number of DTF units, in particular odd versus even numbers, and an extended π‐system influence redox properties, optical transitions, and intermolecular associations. We hypothesized that the increased π‐surface and the planarity of these molecules in the neutral state should promote strong associations both in solution and solid states. Here, we demonstrate this hypothesis to be true, and, gratifyingly, the expanded multi‐DTF PAH structure also allows accessibility to mixed‐valence salts in the solid state, which sets the scene for future work toward new intervalence charge transport materials.

## 2 Results and Discussion

### 2.1 Synthesis

**FIGURE 3:**
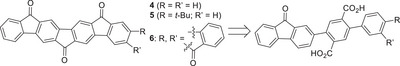
PAH carbonyl target molecules to be prepared by ring closure of carboxylic acid precursors.

We base our synthesis of the novel multi‐DTF PAHs on Horner–Wadsworth–Emmons (HWE) olefination reactions between PAH cores bearing carbonyl functionalities and phosphonate esters of the 1,3‐dithiole rings. This approach provides three PAH cores (**4**, **5**, and **6**) as initial targets to be accessed from dicarboxylic acid precursors as outlined in Figure 3.

**SCHEME 1:**
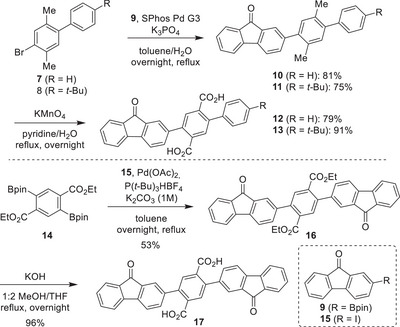
Synthesis of dicarboxylic acids **12**, **13**, and **17**.

Initiated by a Suzuki cross‐coupling reaction between aryl bromide **7** [42] or **8** [43] and boronic ester **9** [44], compounds **10** and **11** were first obtained (Scheme 1). These compounds were subjected to benzylic oxidation using KMnO_4_, yielding dicarboxylic acids **12** and **13**. Suzuki couplings between diboronic ester **14** [45] and compound **15** [46] yielded compound **16** that was hydrolyzed to dicarboxylic acid **17**.

**SCHEME 2:**
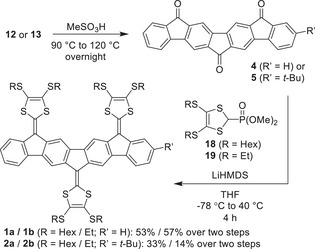
Synthesis of IF‐based extended tri‐DTFs **1a**/**b** and **2a**/**b**.

**SCHEME 3:**
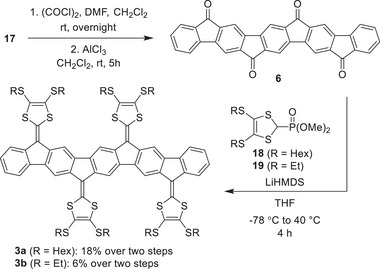
Synthesis of IF‐based extended tetra‐DTF **3**.

PAH cores **4** and **5** were next synthesized by treatment of **12** and **13** with MeSO_3_H via a two‐fold intramolecular electrophilic aromatic substitution (Scheme 2). For PAH core **6**, the intramolecular ring closure was achieved by in situ formation of the acyl chloride by treatment with oxalyl chloride, followed by Friedel–Crafts acylation in the presence of AlCl_3_ (Scheme 3). Initial attempts to synthesize **6** were performed under the same conditions as for formation of **4** and **5**, by treatment of MeSO_3_H. This method proved to be inconsistent for initiating the desired ring closure and produced mainly by‐products. The acyl chloride/Friedel–Crafts strategy therefore proved essential for obtaining **6** in sufficient quality. Ketones **4**, **5**, and **6** exhibited extremely poor solubility in common organic solvents, which complicated purification and characterization. Conventional chromatographic purification was not feasible; instead, crude solids were extensively washed with a range of organic solvents to remove impurities. This procedure proved critical for the subsequent chemical transformation to be successful.

Tri/tetra‐DTFs **1**, **2** (Scheme 2), and **3** (Scheme 3) were synthesized by HWE olefinations of the corresponding PAH‐ketones by treatment with deprotonated dimethyl(1,3‐dithiol‐2‐yl)phosphonate reagents obtained from **18** and **19** [47]. However, **3b** exhibited considerably low solubility, rendering its purification and characterization challenging, and it was therefore not subjected to detailed studies.

### 2.2 Single Crystal X‐ray Diffraction

**FIGURE 4:**
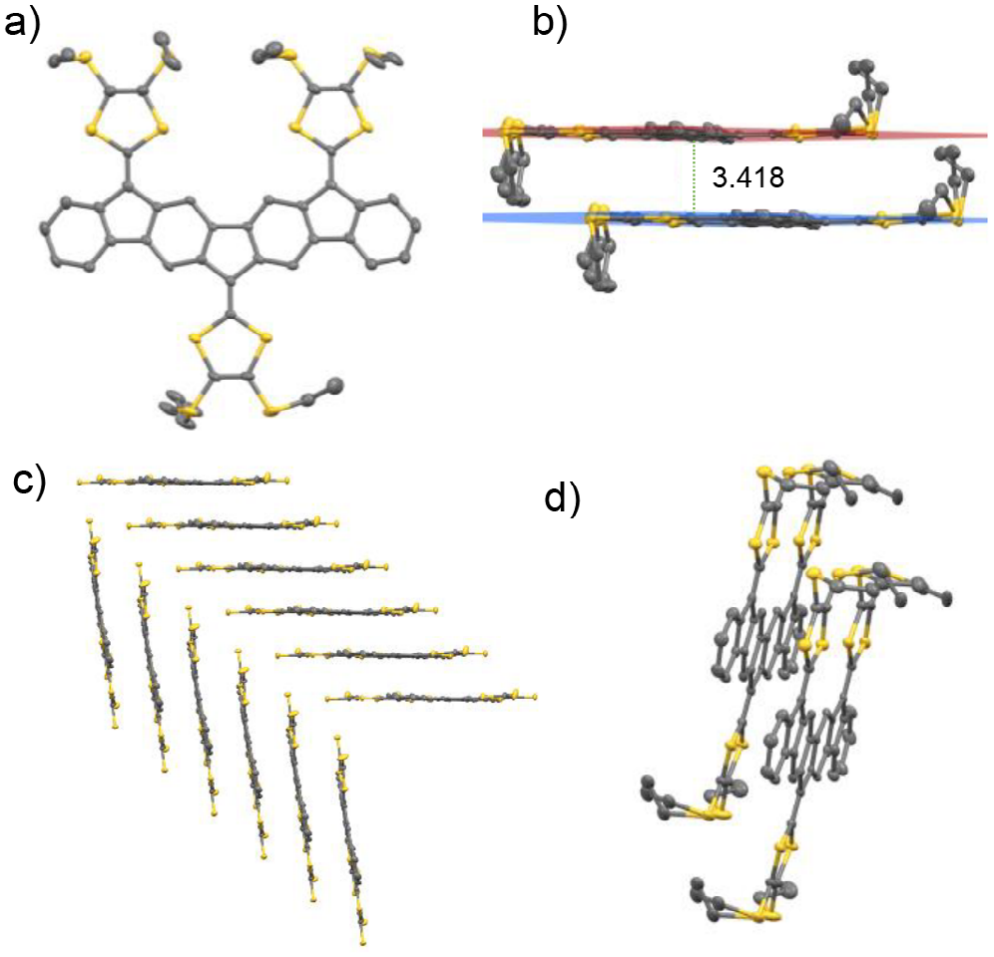
(a) Molecular structure of tri‐DTF **1b** according to X‐ray crystallography; structure shown as 50% probability thermal ellipsoids. One of the ethyl chains is disordered. (b) Stacking of two molecules of **1b** with planes defined as a mean from the atoms in the core and DTF units (excluding the SEt chains) for each monomer. The distance between the two planes is 3.418 Å. (c) Crystal packing of compound **1b** in the monoclinic space group P2_1_/c. Ethyl chains were omitted for clarity. (d) Another view of the stacking of two molecules of **1b**. In all structures, hydrogen atoms were removed for clarity. Carbon and sulfur atoms are colored gray and yellow, respectively.

Single crystals of **1b** suitable for x‐ray diffraction were obtained by slow vapor diffusion of *n*‐pentane into a solution of compound **1b** in chloroform. Figure 4 shows the molecular structure modeled from the x‐ray diffraction data [48]. One ethyl substituent exhibits minor positional disorder.

The structure reveals a nearly co‐planar arrangement of the PAH core and the three DTF units (Figures 4a,b), supporting efficient π‐conjugation across the entire framework. In the solid state, **1b** forms extended π–π stacked columns along the *a*‐axis (Figure 4c). The centroid‐to‐centroid distance between neighboring extended‐IF cores is 3.418 Å, consistent with significant π–π overlap between adjacent molecules.

The crystal packing features two distinct molecular orientations intersecting at an angle of 82.2°, giving rise to a quasi‐herringbone arrangement. A slight translational offset between neighboring molecules is observed, which may help relieve steric congestion from the peripheral DTF groups. Interestingly, all DTF units orient in the same direction within a stack, suggesting directional preferences in intermolecular interactions (Figure 4d). The combination of planarity and directional packing suggests a strong propensity for intermolecular association in the solid state.

### 2.3 NMR Dilution Experiments and DOSY

**FIGURE 5:**
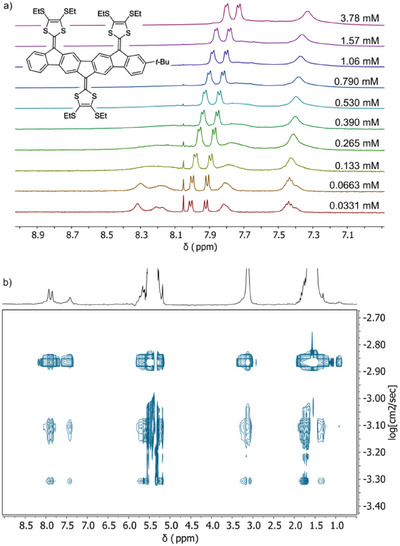
(a) Segment of ^1^H NMR spectroscopic data of tri‐DTF **2b** recorded at different concentrations in CD_2_Cl_2_ at 25°C. (b) DOSY experiment of **2b** recorded at 1.06 mM in CD_2_Cl_2_ at 25°C showing three sets of distinct resonances.

The small **IF‐TTF** is known to undergo intermolecular self‐association with an equilibrium constant of ca. 16 M^−1^ in CDCl_3_ [29, 36]. For the novel multi‐DTF systems, the association was significantly stronger. A ^1^H NMR spectroscopic dilution series was performed for compound **2b** in CD_2_Cl_2_ (Figure 5a), revealing a significant downfield shift of the ^1^H resonances with decreasing concentration. At high concentrations, the ^1^H NMR resonances appeared as broad, overlapping signals. For the related **1a**, the signals remained sharp in the concentration range 0.04–5.5 mM with the central fluorene protons shifting downfield from 8.21 / 8.33 ppm to 7.85 / 7.90 ppm (Figure S36). By curve fitting of chemical shifts against concentration using the online tool *Bindfit* [49], a self‐association constant of ca. 600 M^−1^ ± 10% at 25°C in CD_2_Cl_2_ is estimated (using dimer aggregation model for simplicity). Thus, expanding from a bis‐DTF (**IF‐TTF**) to a tri‐DTF system increases the self‐association constant by a factor of ca. 38 in a chlorinated solvent.

Complementary diffusion‐ordered NMR spectroscopy (DOSY) experiments of compound **2b** at 1.06 mM in CD_2_Cl_2_ further confirmed the aggregation (Figure 5b). The experiment revealed three distinct sets of resonances with different diffusion coefficients, providing clear evidence for the coexistence of multiple aggregate species. While the exact aggregation numbers cannot be determined from DOSY alone, the data strongly support the presence of at least three discrete aggregate states under the studied conditions.

### 2.4 Electrochemical Studies

**FIGURE 6:**
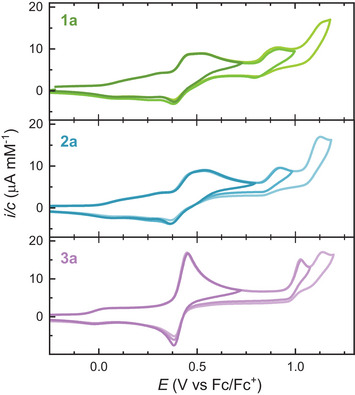
Cyclic voltammograms of **1a** (0.25 mM, top, green), **2a** (0.50 mM, middle, blue) and **3a** (0.25 mM, bottom, purple) recorded in CH_2_Cl_2_ + *n*‐Bu_4_NPF_6_ (0.1 M) at room temperature and a scan rate of 0.1 V/s. All potentials are reported versus Fc/Fc^+^. Full scans including all oxidations are shown in bright color and partial scans in darker colors (the overlapping waves confirm reversible individual oxidations).

The redox behavior of the multi‐DTF compounds was investigated by cyclic voltammetry (CV) and differential pulse voltammetry (DPV) in CH_2_Cl_2_ with 0.1 M *n*‐Bu_4_NPF_6_ as supporting electrolyte. The cyclic voltammograms of the hexyl‐functionalized derivatives (**1a**, **2a**, and **3a**) are shown in Figure 6, while those of the ethyl‐functionalized analogues (**b**) and all DPV traces are provided in the Supporting Information (SI). The corresponding oxidation potentials of **1a**, **2a**, and **3a** derived from DPV are summarized in Table 1 along with electrochemical data of the parent **IF‐TTF** [29, 50].

**TABLE 1:** Electrochemical oxidation potentials derived from DPVs. All potentials are reported versus Fc/Fc^+^.

Compound	*E*_ox(1)_	*E*_ox(2)_	*E*_ox(3)_	*E*_ox(4)_	*E*_ox(5)_	*E*_ox(6)_
**1a**	0.10	0.27	0.41	0.50	0.85^a^	1.08^a^
**2a**	0.08	0.27	0.41	0.50	0.86^a^	1.07^a^
**3a**	0.01			0.41^b^	0.98^a^	1.09^a^
**IF‐TTF** ^c^	0.24	0.42	1.09			

^a^ Quasi‐reversible.

^b^ Observed as a three‐electron oxidation.

^c^ IF‐TTF with peripheral *n*‐butyl groups on DTF units instead of *n*‐hexyl groups [29, 50].

Tri‐DTFs **1a** and **2a** exhibit nearly identical electrochemical responses, indicating that the introduction of a single *tert*‐butyl substituent on the PAH periphery—rendering **2a** unsymmetrical—has little effect on the redox properties. The oxidation processes of **1a** and **2a**, however, are notably complex. This complexity arises from intermolecular associations (vide supra). The oxidation potentials of the different neutral aggregate species should differ, giving rise to multiple oxidation waves for a single‐electron oxidation as a result of oxidizing multiple species. This effect is diffusion dependent and affected by the different size aggregates. For compounds **1a** and **2a**, the first oxidation event appears as two reversible, broad, waves corresponding in total to the transfer of one electron per molecule. Moreover, mixed‐valence complexes between the neutral and radical cationic species further broaden the waves [29]. Accordingly, the first oxidation event in **1a** and **2a** is assigned to the overall formation of the radical cation, encompassing contributions from both monomeric and aggregated species. An electrochemical study at different concentrations was carried out on **2b** (Figure S63). However, the voltammetric profiles remained essentially unchanged upon dilution, suggesting strong and persistent associations even at the lowest concentration required for the measurement.

At higher potentials, two reversible one‐electron oxidations are observed at 0.41 and 0.50 V versus Fc/Fc^+^ for both tri‐DTF compounds **1a** and **2a**, corresponding to formation of the dication and trication, respectively. The reversibility of these redox processes maintained over multiple CV cycles (Figure S64), which indicates that the oxidized species are chemically stable under the conditions. Two additional quasi‐reversible oxidations appear at still higher potentials and are tentatively assigned to oxidation of the PAH core.

**FIGURE 7:**
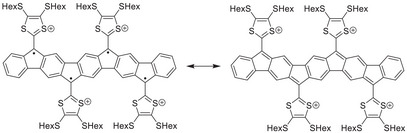
Two borderline case resonance structures of **3** ^4+^, open‐shell (left) and closed‐shell (right).

Interestingly, tetra‐DTF **3a** displays a distinctly different redox profile (Figure 6, bottom). A reversible one‐electron oxidation at 0.01 V leads to formation of the radical cation. The oxidation wave is significantly broad, indicating again associations between neutral species and formation of mixed‐valence and π‐dimer complexes (and likely higher‐order complexes). At higher potential, a reversible three‐electron oxidation at 0.41 V is observed, resulting in the direct generation of the tetracation. The absence of distinct intermediate oxidation steps suggests a highly cooperative multielectron process, in which full oxidation of all DTF units is thermodynamically favored. This concerted behavior likely originates from strong electronic coupling through the extended IF‐core, enabling extensive charge delocalization and stabilization of the free radicals through rearrangement to the quinoid structure (Figure 7). ESR studies show, however, that it also exhibits some open‐shell character (vide infra). Similar multielectron oxidation events have previously been reported for other extended TTF systems, typically associated with favorable structural reorganization, for example, through relief of molecular strain or increased aromatic stabilization within the conjugated core separating the DTF units [37, 38, 49]. The first two oxidation processes of **3a** remain fully reversible over repeated cycles (Figure S65), confirming the intrinsic chemical stability of the oxidized states. At last, two quasi‐reversible oxidations were observed for **3a**, corresponding to oxidation of the core. Tetra‐DTF derivative **3b** showed a similar oxidation profile, exhibiting initialy a one‐electron oxidation followed by a three‐electron oxidation, supporting the hypothesis of a highly stabilized tetracation species (Figure S61). The three‐electron process appeared, however, as a sharp peak in the CV, presumably due to adsorption at the electrode as a result of the limited solubility of **3b**.

The new IF‐based multi‐DTF systems exhibit significantly lower first oxidation potentials compared to the parent **IF‐TTF** compound. As summarized in Table 1, **IF‐TTF** undergoes two sequential, reversible one‐electron oxidations at 0.24 and 0.42 V versus Fc/Fc^+^ to form the radical cation and dication, respectively [29, 50]. In contrast, the first oxidation potentials of **1a**, **2a**, and **3a** are markedly decreased to 0.10, 0.08, and 0.01 V, respectively. This systematic decrease reflects the increased electron‐donating character conferred by additional DTF units and the enhanced associations between molecules. Interestingly, the second oxidation potential of **1a** and **2a** nearly coincides with the first oxidation of **IF‐TTF**, while the third oxidation of these compounds occurs at potentials similar to the second oxidation of both **3a** and **IF‐TTF**.

### 2.5 Optical Properties of Neutral Molecules

**FIGURE 8:**
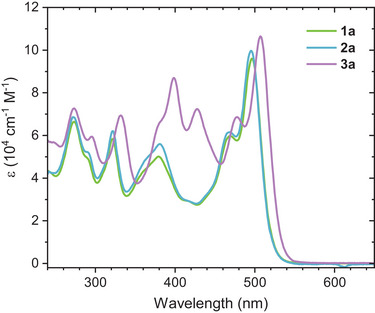
UV–vis absorption spectra of **1a** (green), **2a** (blue), and **3a** (purple) measured in CH_2_Cl_2_ at room temperature.

The optical properties were investigated by UV–vis absorption spectroscopy in CH_2_Cl_2_. The spectra of **1a**, **2a**, and **3a** are shown in Figure 8, while those of the ethyl analogues are provided in the Supporting Information. Absorption maxima and extinction coefficients are summarized in Table S3.

The tri‐DTF derivatives **1a** and **2a** show nearly identical absorption profiles with a longest‐wavelength band at 496 nm, confirming that the peripheral *tert*‐butyl group in **2a** has no significant effect on the electronic structure. For the tetra‐DTF compound **3a**, the lowest‐energy transition appears at 507 nm, representing a modest bathochromic shift of 11 nm relative to **1a** and **2a**. This trend is consistent with that observed for the parent **IF‐TTF**, which absorbs at 475 nm [29]. The progressive redshift of approximately 10–20 nm per additional DTF unit indicates a systematic extension of π‐conjugation. The intensity of the lowest‐energy transition also increases with the number of DTF units, with **3a** exhibiting the highest extinction coefficient within the series. Beyond the low‐energy transitions, the introduction of one or more DTF units significantly alters the high‐energy absorption region compared to **IF‐TTF**. For **1a** and **2a**, new intense bands appear at 322 and 379–391 nm, while **3a** displays an analogous feature at 332 nm, and new bands at 399 and 428 nm.

### 2.6 Optical Properties of Oxidized Species

**FIGURE 9:**
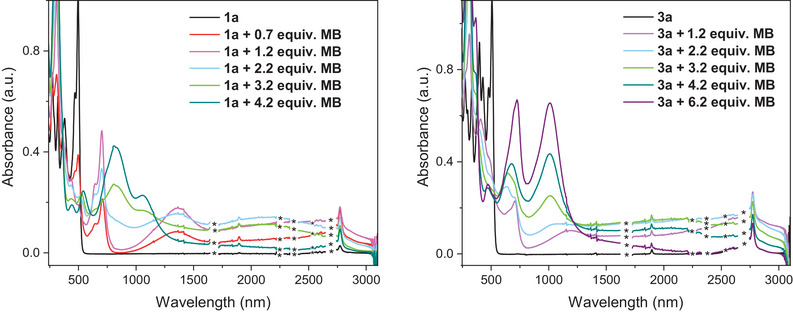
UV–vis–NIR absorption spectra of **1a** (left) and **3a** (right) with addition of different equivalents of tris(4‐bromophenyl)ammoniumyl hexachloroantimonate (‘magic blue", **MB**), recorded in CH_2_Cl_2_ at room temperature on 0.008 mM solutions. Spectral defects in the NIR‐area (1500–3000 nm) have been removed for clarity—indicated by asterisks—full spectra are found in the Supporting Information.

To further elaborate on the intermolecular interactions of the oxidized species, UV–vis–NIR absorption spectroscopy was conducted for tri‐DTF **1a** and tetra‐DTF **3a** in CH_2_Cl_2_. Spectra were recorded after addition of increasing equivalents (equiv.) of tris(4‐bromophenyl)ammoniumyl hexachloroantimonate (‘magic blue’, **MB**), previously used as a chemical oxidant for extended TTF derivatives [36, 51, 52]. Addition of 0.7 equiv. of **MB** generated a mixture of radical cation species **1a^•+^** and neutral **1a**, characterized by the emergence of new absorption bands spanning 700–3000 nm (Figure 9, left, red curve). A distinct band centered at ca. 1360 nm was observed. Upon addition of 1.2 equiv. **MB**, a particularly broad absorption extending from 1600 to 3000 nm was observed, accompanied by an increase in the 1360‐nm band, indicating the formation of mixed‐valence and π‐dimer complexes (pink curve). Upon further oxidation to **1a^2+^** (2.2 equiv. **MB**, blue curve), the broad NIR absorption intensifies while the 1360‐nm feature diminishes. Comparable NIR transitions have previously been observed for chemically oxidized IF‐TTF derivatives, where bands near 1990, 1650, and 1450 nm were attributed to isolated radical cations, mixed‐valence species, and π‐dimers, respectively [29]. In contrast, for the extended multi‐DTF architecture of **1a**, the NIR response is substantially broadened and less structured, consistent with the strong associations of molecules in cationic complexes. The 1360 nm band likely arises from intermolecular π‐dimerization of radical cations, which is gradually suppressed as oxidation progresses.

Upon addition of 3.2 equiv. of **MB**, the NIR features gradually diminish, while new absorption bands at 830 and 1050 nm emerge, consistent with formation of the tricationic species (green curve). However, close to complete disappearance of the NIR bands is not achieved until 4.2 equiv. of **MB** are added under these conditions, accompanied by intensified visible absorption, indicating full oxidation of all DTF units to **1a^3+^** (cyan curve).

Chemical oxidation of the tetra‐DTF compound **3a** similarly revealed the formation of strongly interacting oxidized species (Figure 9, right). Addition of 1.2 equiv. of **MB** produced new absorptions at 1185 nm and a broad band spanning 1300–3000 nm (pink curve), attributed to π‐dimer and mixed‐valence aggregates of **3a^•+^** (as well as monomeric species—intrinsic absorption). Oxidation with 2.2 equiv. of **MB** (blue curve) caused only minor spectral changes, reflecting a greater stabilization and oxidation resistance of **3a** relative to **1a**. At 3.2 equiv. of **MB** (green curve), new bands emerged at 640 and 1010 nm, indicating partial formation of higher oxidation states while lower‐valent species persist. Continued oxidation with 4.2 equiv. **MB** (cyan curve) led to increased absorption around 1100 nm and a concurrent decrease of the broad NIR band, signaling progressive oxidation. Full conversion to **3a^4+^** was achieved after 6.2 equiv. of **MB** (purple curve), evidenced by the disappearance of the NIR continuum accompanied by an increase in the tetracation absorption at 1100 nm. The sharp peak at 725 nm corresponds to excess **MB** due to slight overtitration.

No concentration dependence of the spectral features was observed when oxidation of **1a** and **3a** with 1.2 equiv. **MB** was repeated at various concentrations (Figures S67 and S68), confirming that the mixed‐valence and π‐dimer species (or higher oligomers) formed in both systems are strongly associated, persistent aggregates rather than transient bimolecular species.

Overall, the chemical oxidation study highlights the exceptional electronic delocalization and redox cooperativity inherent to these multi‐DTF IF systems. The low first oxidation potentials observed by cyclic voltammetry (0.10 V for **1a**, 0.01 V for **3a**) correspond to the formation of delocalized radical cations stabilized by π–π interactions, as evidenced by the broad NIR absorption. This suggests that oxidation of aggregated mixed‐valence species and charge delocalization across multiple DTF units extend beyond simple one‐electron redox equilibria, emphasizing the strong electronic coupling and self‐association.

### 2.7 ESR Studies of Oxidized Species

ESR measurements were performed on tri‐DTF **1a** (0.1 mM) and tetra‐DTF **3a** (0.1 mM) after chemical oxidation with **MB** in CH_2_Cl_2_ (Figures S70 and S71). The radical cation of **1a** shows a signal, which almost disappears for the dication, signalling closed‐shell character of **1a^2+^**. Further oxidation to the trication regenerates an ESR signal, but of significantly weaker intensity as that of the monocation. In contrast, the ESR signal increases for **3a** when proceeding from the radical cation to the dication, signalling significant open‐shell character of **3a^2+^**. This is reasonably explained by generation of a positively charged 1,3‐dithiolium ring at each end of the molecule and hence localization of an unpaired electron at each end. Further oxidation to the trication reduces the ESR signal, and it is further reduced for the tetracation on account of electron pairing. The fact that a small signal is still present for **3a^4+^** indicates, however, that the tetracation should not solely be described by the closed‐shell structure shown in Figure 7, but likely also by some diradical character.

### 2.8 Electrocrystallization

Tri‐DTF **1b** was subjected to electrochemical oxidation in an *H*‐shaped cell (Figure S1) to induce crystallization of oxidized salts. Two electrocrystallization attempts were performed in anhydrous chlorobenzene containing *n‐*Bu_4_NPF_6_ as supporting electrolyte, following a previously reported procedure [36]. In the first attempt, a 0.1‐M *n‐*Bu_4_NPF_6_ solution was used and a constant current of 1.0 µA was applied, leading to the formation of a dark solid material at the anode after 2 days. After 5 days, the anode was removed, and the deposited solids were collected, washed thoroughly with chlorobenzene and methanol, and dried under a stream of nitrogen. In the second attempt, a 0.05‐M *n‐*Bu_4_NPF_6_ solution was used and a current of 1.5 µA was employed. The experiment was allowed to proceed for 4 days, whereafter the resulting precipitate was isolated.

**FIGURE 10:**
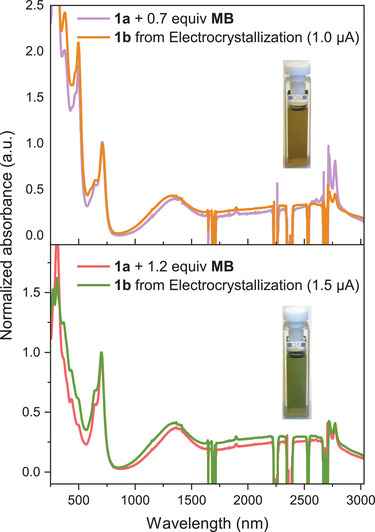
UV–vis–NIR absorption spectra of **1a** + 0.7 equiv. of **MB** (violet curve) and electrocrystallized material of **1b** obtained at low current (orange curve) (top) and of **1a** + 1.2 equiv. **MB** (red curve) and electrocrystallized material of **1b** obtained at high current (green curve) (bottom) measured in CH_2_Cl_2_, showing excellent resemblance.

UV–vis–NIR absorption spectra of the collected materials were recorded in CH_2_Cl_2_ and compared to those of oxidized **1a** (Figure 10). Given the structural similarity between **1a** and **1b**, comparable spectral characteristics are expected. The product obtained at low current (1.0 µA) exhibited absorption features consistent with those observed for **1a** upon oxidation with 0.7 equiv. of **MB**, showing similar bands in the 300–500 nm region. This suggests that the isolated salt predominantly consists of a mixture of **1b^•+^** and neutral **1b**, corresponding to a mixed‐valence state (Figure 10, top). In contrast, the material obtained from the experiment at higher current (1.5 µA) displayed an absorption profile matching that of **1a** oxidized with 1.2 equiv. of **MB**, indicative of the **1b^•+^** π‐dimer salt (Figure 10, bottom). These results suggest that electrocrystallization at lower current favors formation of mixed‐valence salts, whereas higher current promotes generation of the radical cation π‐dimer species. This is interesting in view of the fact that previous attempts to promote neutral/radical cation mixed‐valence salts of the smaller **IF‐TTF** all failed [29].

## 3 Conclusion

In conclusion, very long PAHs with alternating six‐ and five‐membered rings and incorporating three or four DTF units were successfully prepared via HWE olefination reactions. These molecules exhibited very strong associations between neutral and/or cationic states, reflected by very broad first oxidation waves in the cyclic voltammograms and NIR absorptions of the oxidized species. Moreover, a remarkable difference in the redox properties was observed between odd‐ and even‐numbered DTF structures. Thus, while stepwise oxidations was observed for the tri‐DTF PAH structure, the tetra‐DTF PAH structure revealed a broad one‐electron oxidation followed by a three‐electron oxidation, indicating a strong propensity to form the tetracation. Each redox state had distinct UV–vis–NIR absorptions, rendering these compounds promising for electrochromic materials.

By careful control of the current, electrocrystallization experiments on the tri‐DTF compound provided either mixed‐valence or monocation salts. Thus, the one‐dimensional expansion of the IF‐TTF motif is a promising way of generating neutral/radical cation salts that were previously never obtained for the smaller IF‐TTF structures. In the future, we hope to be able to obtain single crystals of the mixed‐valence salt to elucidate in detail molecular packing and conductivities.

## Conflicts of Interest

The authors declare no conflicts of interest.

## Supporting information




**Supporting File 1**: Experimental details for synthetic protocols, self‐association studies (NMR dilution), electrochemical, UV–vis absorption, UV–vis–NIR absorption, ESR, electrocrystallization and crystallographic details along with all characterization data can be found in Supporting Information. The authors have cited additional references within the Supporting Information [53, 54, 55, 56, 57, 58].

**Supporting File 2**: anie71734‐sup‐0002‐Data.zip.

## Data Availability

The data that support the findings of this study are available from the corresponding author upon reasonable request.
