# Supplementary material for: One‐Dimensional Polycyclic Aromatic Hydrocarbons Incorporating Multiple Dithiafulvene Units—Novel Multi‐Redox and Electrochromic Systems
Source: Angew Chem Int Ed Engl. 2026 Mar 5;65(16):e25025. doi: 10.1002/anie.202525025 (PMC13080414; doi:10.1002/anie.202525025)
Supplement: Supplementary file 1 — Supporting File 1: Experimental details for synthetic protocols, self‐association studies (NMR dilution), electrochemical, UV–vis absorption, UV–vis–NIR absorption, ESR, electrocrystallization and crystallographic details along with all characterization data can be found in Supporting Information. The authors have cited additional references within the Supporting Information [53, 54, 55, 56, 57, 58]. [file ANIE-65-e25025-s002.pdf]

Contents

General Methods....2
UV-Vis Absorption Spectroscopy....2
Electrochemistry....2
Chemical Oxidation....2
ESR Spectroscopy....2
Electrocrystallization....2
X-Ray Crystallography....3
Synthetic Protocols....4
NMR Spectra....13
NMR Dilution Study of 1a....30
HRMS Spectra....32
UV-Vis Absorption Spectra....47
Electrochemistry Data....51
Chemical Oxidation....54
ESR Measurements....56
Crystallography....59
References....70

General Methods

All commercially available materials were used without further purification, unless stated otherwise. Flash column chromatography was performed using silica gel (Silica gel 60 (43–60 μm) purchased from VWR). NMR spectra were recorded on a Bruker instrument equipped with an observe cryoprobe or a cryo-inverse probe instrument (Novo Nordisk Foundation NMR facility at the Department of Chemistry, University of Copenhagen; Grant: NNF21OC0067315) at 500 MHz and 126 MHz for ^{1}H and ^{13}C NMR spectroscopy, respectively. Spectra were recorded at 25 °C and referenced using internal residues: $CDCl_3$ (δ_H = 7.26 ppm, δ_C = 77.16 ppm), CD_2Cl_2 (δ_H = 5.32 ppm, δ_C = 53.84 ppm), CS_2 (DMSO-d_6 lock tube) (δ_H = 2.50 ppm, δ_C = 39.52 ppm), DMSO-d_6 (δ_H = 2.50 ppm, δ_C = 39.52 ppm). Chemical shift values are referenced to the ppm scale; coupling constants are expressed in Hertz (Hz); and apparent multiplicities are reported as s (singlet), d (doublet), t (triplet), q (quartet), dd (doublet of doublets), td (triplet of doublets), dt (doublet of triplets) or m (multiplet). HRMS analysis was performed on a Bruker SolariX XR MALDI-FT-ICR instrument with dithranol as a matrix. All solvents used were HPLC grade from VWR and used without further purification. Anh. Tetrahydrofuran (THF) was obtained by distillation from sodium and benzophenone under N_2.

UV-Vis Absorption Spectroscopy

All measurements were carried out in 10-mm quartz cuvettes from Hellma using a Varian Cary 50 UV-Vis spectrophotometer. All spectra were recorded in HPLC grade CH_2Cl_2 at room temperature. Baseline corrections were performed with a blank sample.

Electrochemistry

Cyclic voltammograms (CV) and differential pulse voltammograms (DPV) were performed using an Autolab PGSTAT12 instrument and Nova 1.11 software. A platinum disk electrode (1.6 mm) was used as the working electrode, a platinum wire as the counter electrode, and a silver wire (isolated by a ceramic frit) as the reference electrode. Measured potentials were referenced to the ferrocene/ferrocenium (Fc/Fc^+) redox couple, measured before and after the experiment for a 1.0-mM solution of Fc. All measurements were carried out in a 0.1-M n-Bu_4NPF_6 buffer solution in HPLC grade CH_2Cl_2 at 25 °C with a 0.1-V scan rate for CVs. All compounds were measured at 0.25-mM concentrations in CH_2Cl_2 with the exception of compound **2a** and **2b**, which were measured at 0.5-mM.

Chemical Oxidation

Compounds **1a** and **3a** were subjected to chemical oxidation using tris(4-bromophenyl)ammoniumyl hexachloroantimonate 'Magic Blue' (MB) as oxidizing agent. UV-Vis-NIR absorption measurements were performed of the oxidized species on an Agilent Cary 5000 UV-Vis-NIR spectrophotometer. All spectra were recorded in HPLC grade CH_2Cl_2 at room temperature.

ESR Spectroscopy

ESR samples of compounds **1a** and **3a** were prepared under a N_2 atmosphere in anh. CH_2Cl_2 at a final compound concentration of 0.10 mM and chemically oxidized by addition of the desired equivalents of MB.

ESR measurements were performed at room temperature on a commercial Bruker Elexsys E580 spectrometer fitted with a Flexline ER 4118X-MD5 Resonator. Measurements were performed at X-

Band (ca. 9.7 GHz) with a modulation frequency of 100 kHz, a modulation amplitude of 1 G, and a microwave power of 4.743 mW. The magnetic field was centered at B_0 = 3434 G with scan widths (Bscan) of 1000 G (a scan width of 6868 G was used for the neutral spectrum of **1a**). All measurements were carried out at the Novo Nordisk Foundation Copenhagen Pulse EPR Facility.

Electrocrystallization

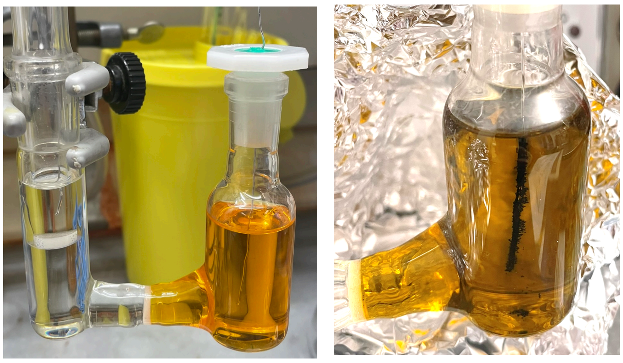

Start *After 4 days*

Figure S1. Photos showing electrocrystallization of tri-DTF **1b** in PhCl with Bu_4NPF_6 as electrolyte (0.05 M) (I = 1.5 µA). After four days, precipitation on the anode was observed and harvested.

Two separate electrocrystallization experiments were performed on compound **1a**. Both experiments were performed in a flame-dried H-shaped cell with two chambers separated by a ceramic frit. In one chamber was added a solution of supporting electrolyte n-Bu_4NPF_6 in anh. chlorobenzene and to the larger chamber was added a solution of **1b** in chlorobenzene, and the solvent levels were left to equilibrate. A platinum wire was placed in each chamber connected to the cathode for the electrolyte solution and to the anode for the compound solution. A current was applied and the setup covered in tin foil to protect the experiment from light.

Table S1. Employed conditions in the two electrocrystallization experiments of **1b**. Solvent: PhCl

Entry #	Electrolyte n-Bu_4NPF_6	Conc. of **1b**	Current	Time
1	0.1 M	0.3 mM	1.5 µA	4 days
2	0.05 M	0.2 mM	1.0 µA	5 days

X-Ray Crystallography

Compound **1b** was recrystallized by vapor diffusion (chloroform/pentane). A clear orange, needle-shaped crystal was mounted on the goniometer. Data were collected from a shock-cooled single crystal at 100.00 K on a Bruker D8 Venture and a Photon III 28 detector. The diffractometer used MoK_α radiation (λ = 0.71073 Å). All data were integrated with SAINT V8.40B, and a multi-scan

absorption correction using SADABS 2016/2 was applied.[53,54] The structure was solved by dual methods with SHELXT and refined by full-matrix least-squares methods against F^2 using SHELXL 2019/3.[55,56] All non-hydrogen atoms were refined with anisotropic displacement parameters. All C-bound hydrogen atoms were refined isotropic on calculated positions using a riding model with their U_{iso} values constrained to 1.5 times the U_{eq} of their pivot atoms for terminal sp^3 carbon atoms and 1.2 times for all other carbon atoms. Disordered moieties were refined using bond length restraints and displacement parameter restraints. Crystallographic data for the structures reported in this paper have been deposited with the Cambridge Crystallographic Data Centre.[57] CCDC 2501210 contains the supplementary crystallographic data for this paper. These data can be obtained free of charge from The Cambridge Crystallographic Data Centre via www.ccdc.cam.ac.uk/structures. This report and the CIF file were generated using FinalCif.[58]

Synthetic Protocols

Compound **1a**

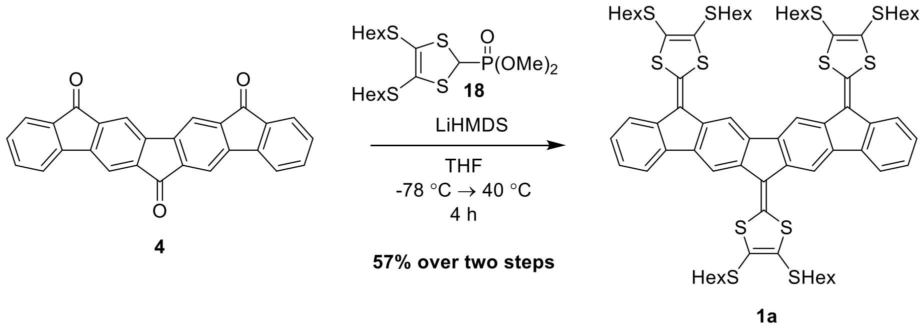

To a flame-dried Schlenk flask under a positive flow of N_2 was added phosphonate ester **18**[47] (1.40 mg, 3.15 mmol) and anh. THF (30 mL). The solution was degassed and cooled to -78 °C on a dry ice/acetone bath for 30 min. LiHMDS (1 M in toluene, 3.20 mL, 3.20 mmol) was added dropwise, and the reaction mixture was stirred at -78 °C for 1 h. To a separate flame-dried Schlenk flask was added compound **4** (150 mg) in anh. THF (30 mL). The suspension was degassed and cooled to -78 °C on a dry ice/acetone bath for 30 min before the solution containing **18** was added fast via cannulation. After 1 h the dry ice/acetone bath was removed, and the reaction mixture was allowed to reach room temperature for 1 h before it was heated to 40 °C for 1 h. The reaction was quenched with aqueous NH_4Cl (sat., 40 mL), washed with H_2O (50 mL), and extracted with CH_2Cl_2 (3 x 70 mL). The combined organic phase was dried over $MgSO_4$ and concentrated *in vacuo*. The product was purified by flash column chromatography (SiO_2, 50% CH_2Cl_2/heptane). The product was redissolved in CH_2Cl_2 and the solution dropwise added to cold MeOH, resulting in precipitation of the product. This yielded compound **1a** (350 mg, 0.261 mmol, 57% over two steps) as an orange solid. ^{1}H NMR (500 MHz, $CDCl_3$) δ 7.97 (s, 2H), 7.90 (s, 2H), 7.84 (dd, *J* = 7.0, 1.5 Hz, 2H), 7.69 (d, *J* = 7.6 Hz, 2H), 7.37 (td, *J* = 7.6, 1.5 Hz, 2H), 7.33 (td, *J* = 7.0, 1.2 Hz, 2H), 3.02 (dt, *J* = 15.2, 7.4 Hz, 8H), 2.96 (t, *J* = 7.4 Hz, 4H), 1.81 – 1.69 (m, 12H), 1.53 – 1.43 (m, 12iH), 1.23 – 1.29 (m, 24H), 0.93 – 0.89 (m, 12H), 0.87 – 0.82 (m, 6H). ^{13}C NMR (126 MHz, $CDCl_3$) δ 138.77, 137.86, 137.81, 137.14, 136.32, 135.85, 135.59, 135.02, 129.63, 128.59, 128.00, 126.52, 125.58, 123.05, 121.42, 121.37, 119.49, 114.13, 113.91, 37.05, 36.77, 36.73, 31.62, 31.58, 31.54, 30.12, 30.04, 30.00, 28.54, 28.53, 28.51, 22.77, 22.75, 22.74, 14.21, 14.20, 14.19. HRMS (MALDI$^+$ FT-ICR, dithranol): *m/z* = 1338.3695 $[M]^{•+}$, calcd. for $[C_{72}H_{90}S_{12}^{•+}]$ *m/z* = 1338.3686.

Compound **1b**

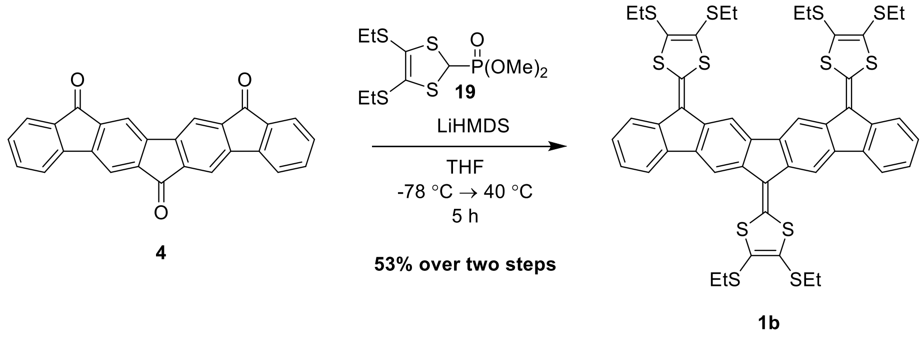

To a flame-dried Schlenk flask under a positive flow of N_2 were added phosphonate ester **19**[47] (961 mg, 2.89 mmol) and anh. THF (30 mL). The solution was degassed and cooled to -78 °C on a dry ice/acetone bath for 30 min. LiHMDS (1 M in toluene, 3.20 mL, 3.20 mmol) was added dropwise, and the reaction mixture was stirred at -78 °C for 1 h. To a separate flame-dried Schlenk flask was added compound **4** (150 mg) in anh. THF (30 mL). The suspension was degassed and cooled to -78 °C on a dry ice/acetone bath for 30 min before the solution containing **19** was added fast via cannulation. After 1 h the dry ice/acetone bath was removed and the reaction mixture was allowed to reach room temperature for 30 min before it was heated to 40 °C for 2 h. The reaction was quenched with aq. NH_4Cl (sat., 20 mL), washed with H_2O (50 mL), and extracted with CH_2Cl_2 (3 x 70 mL). The combined organic phase was dried over $MgSO_4$ and concentrated *in vacuo*. The product was purified by filtration through a plug of SiO_2 (50% CH_2Cl_2/CS_2) and concentrated. The product was redissolved in CH_2Cl_2 and the solution dropwise added to cold MeOH, resulting in precipitation of the product. This yielded compound **1b** (244 mg, 0.243 mmol, 53% over two steps) as an orange solid. ^{1}H NMR (500 MHz, $CDCl_3$) δ 7.93 (s, 2H), 7.90 (s, 2H), 7.84 (dd, *J* = 6.6, 1.8 Hz, 2H), 7.67 (dd, *J* = 6.6, 1.3 Hz, 2H), 7.37 – 7.31 (m, 4H), 3.09 – 2.96 (m, 12H), 1.51 – 1.34 (m, 18H). ^{13}C NMR (126 MHz, $CDCl_3$) δ 138.75, 137.88, 137.78, 137.20, 136.12, 135.61, 135.03, 129.35, 128.76, 128.06, 126.55, 125.64, 123.07, 121.52, 121.45, 119.51, 114.14, 113.91, 31.14, 30.98, 30.91, 15.63, 15.29, 15.21. HRMS (MALDI$^+$ FT-ICR, dithranol): *m/z* = 1001.9936 $[M]^{\bullet+}$, calcd. for $[C_{48}H_{42}S_{12}^{\bullet+}]$ *m/z* = 1001.9930.

Compound **2a**

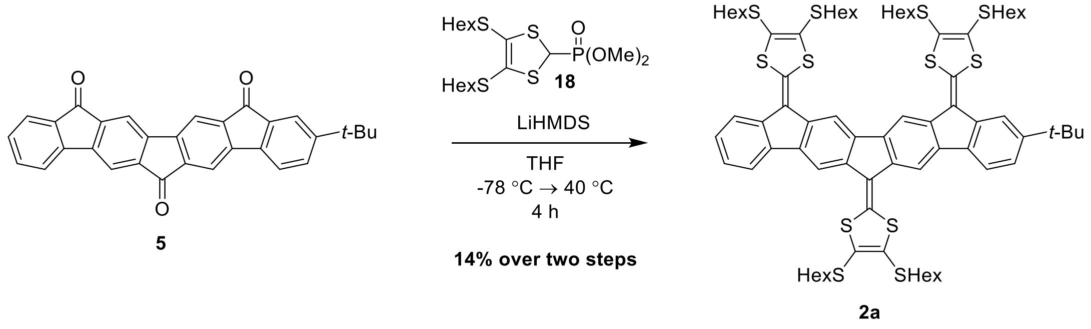

To a flame-dried flask were added phosphonate ester **18**[47] (514 mg, 1.16 mmol) and anh. THF (20 mL). The solution was degassed with N_2 for 20 min and cooled to -78 °C on a dry ice/acetone bath. LiHMDS (1 M in toluene, 1.20 mL, 1.20 mmol) was added dropwise, and the solution was stirred at

-78 °C for 1 h. To a separate flame-dried flask were added compound **5** (56.0 mg) and anh. THF (25 mL). The suspension was degassed with N_2 for 30 min, sonicated for 20 min and cooled to -78 °C on a dry ice/acetone bath before the solution containing **18** was added fast via cannulation. After 20 min, the dry ice/acetone bath was removed, and the reaction mixture was allowed to reach room temperature for 30 min before it was heated to 40 °C for 2 h. The reaction was quenched with aq. NH_4Cl (sat., 50 mL) and extracted with CH_2Cl_2 (3 x 30 mL). The combined organic phase was dried over $MgSO_4$ and concentrated *in vacuo*. The product was purified by flash column chromatography (SiO_2, 10 – 30% CH_2Cl_2/heptane) followed by recrystallization from CH_2Cl_2/MeOH and subsequent trituration with MeOH (3 x 7 mL). This yielded compound **2a** (24.0 mg, 17.2 µmol, 14% over two steps) as a red solid. ^{1}H NMR (500 MHz, CD_2Cl_2) δ 8.06 (s, 1H), 8.03 (s, 1H), 7.93 (s, 2H), 7.85 (d, J = 7.4 Hz, 1H), 7.79 (d, J = 7.9 Hz, 1H), 7.76 (d, J = 1.6 Hz, 1H), 7.67 (d, J = 7.2 Hz, 1H), 7.41 (dd, J = 7.9, 1.6 Hz, 1H), 7.41 (td, J = 7.4, 1.4 Hz, 1H), 7.32 (td, J = 7.2, 1.4 Hz, 1H), 3.09 – 2.96 (m, 12H), 1.83 – 1.71 (m, 12H), 1.52 (s, 9H), 1.51 – 1.48 (m, 12 H), 1.39 – 1.28 (m, 24H), 0.94 – 0.87 (m, 12H), 0.87 – 0.81 (m, 6H). ^{13}C NMR (126 MHz, CD_2Cl_2) δ 150.40, 138.77, 138.04, 138.02, 137.95, 137.56, 137.54, 137.22, 137.21, 136.78, 136.30, 135.46, 135.15, 130.10, 130.08, 129.00, 128.98, 128.24, 127.97, 126.86, 125.82, 123.39, 123.25, 121.73, 121.20, 120.43, 119.70, 119.33, 114.32, 114.25, 114.18, 114.05, 37.31, 37.03, 37.01, 36.95, 35.44, 31.96, 31.90, 31.86, 31.84, 31.81, 31.79, 30.55, 30.41, 30.39, 30.37, 30.34, 30.30, 28.80, 28.76, 28.74, 23.04, 23.03, 23.02, 23.00, 14.24, 14.23. 4 sp^2-carbon and 12 sp^3-carbon signals missing presumably due to overlap. HRMS (MALDI$^+$ FT-ICR, dithranol): m/z = 1395.4319 $[M]^{\bullet+}$, calcd. for $[C_{76}H_{98}S_{12}^{\bullet+}]$ m/z = 1395.4312.

Compound **2b**

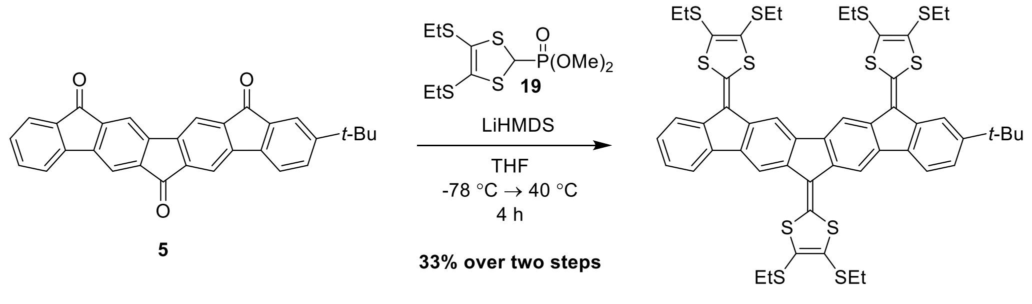

To a flame-dried flask were added phosphonate ester **19**[47] (386 mg, 1.16 mmol) and anh. THF (20 mL). The solution was degassed with N_2 for 20 min and cooled to -78 °C on a dry ice/acetone bath. LiHMDS (1 M in toluene, 1.17 mL, 1.17 mmol) was added, and the solution was stirred at -78 °C for 1 h. To a separate flame-dried flask were added compound **5** (53.0 mg) and anh. THF (20 mL). The suspension was degassed with N_2 for 30 min, sonicated for 20 min and cooled to -78 °C on a dry ice/acetone bath before the solution containing **19** was added fast via cannulation. After 20 min, the dry ice/acetone bath was removed, and the reaction mixture was allowed to reach room temperature for 30 min before it was heated to 40 °C for 2 h. The reaction was quenched with aq. NH_4Cl (sat., 50 mL) and extracted with CH_2Cl_2 (3 x 30 mL). The combined organic phase was dried over $MgSO_4$ and concentrated *in vacuo*. The product was purified by flash column chromatography (SiO_2, 20 – 50% CH_2Cl_2/heptane), which yielded compound **2b** (45.0 mg, 42.5 µmol, 35%) as a red solid. ^{1}H NMR (500 MHz, CS_2 with DMSO-d_6 lock tube) δ 7.79 (s, 1H), 7.78 (s, 1H), 7.68 (s, 1H), 7.64 (s, 1H), 7.53 (dd, J = 6.5, 1.7 Hz 1H), 7.45 (d, J = 7.9 Hz, 1H), 7.37 – 7.35 (m, 2H), 7.09 – 7.00 (m, 3H), 2.84 – 2.74 (m, 12H), 1.30 – 1.15 (m, 27H). ^{13}C NMR (126 MHz, CS_2 with DMSO-d_6 lock tube) δ 148.66, 137.90, 137.28, 137.14, 136.96, 136.82, 136.78, 136.57, 135.51, 135.32, 135.13, 135.04, 134.79, 134.62, 134.49, 128.73, 128.68, 128.55, 128.29, 127.98, 127.56, 125.92, 125.34, 122.70,

122.57, 121.61, 121.28, 121.15, 119.60, 118.85, 118.53, 113.84, 113.70, 113.68, 113.55, 34.16, 31.26, 30.82, 30.81, 30.79, 30.69, 30.62, 15.13, 15.10, 14.89, 14.86, 14.82. 1 sp^2-carbon signal and 2 sp^3-carbon signals missing presumably due to overlap. HRMS (MALDI$^+$ FT-ICR, dithranol): *m/z* = 1058.0528 $[M]^{\bullet+}$, calcd. for $[C_{52}H_{50}S_{12}^{\bullet+}]$ *m/z* = 1058.0555.

Compound **3a**

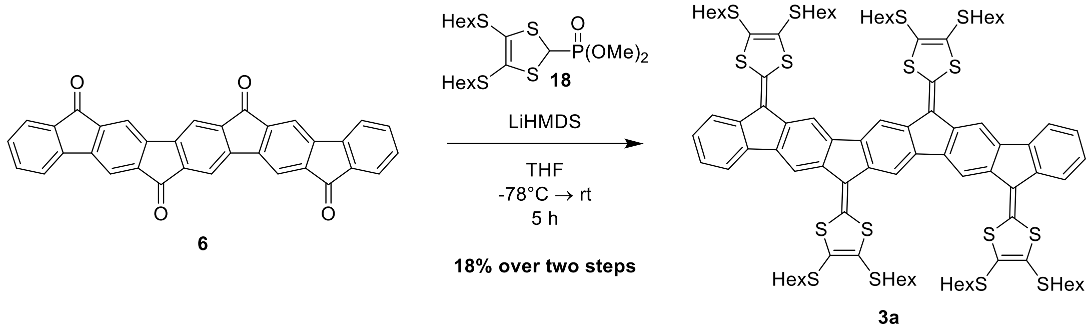

To a flame-dried Schlenk flask under a positive flow of N_2 were added phosphonate ester **18**[47] (661 mg, 1.49 mmol) and anh. THF (10 mL). The solution was degassed and cooled to -78 °C on a dry ice/acetone bath for 30 min. LiHMDS (1 M in toluene, 1.5 mL, 1.50 mmol) was added dropwise, and the reaction mixture was stirred at -78 °C for 1 h. To a separate flame-dried Schlenk flask were added compound **6** (70 mg, 0.144 mmol) and anh. THF (10 mL). The suspension was degassed and cooled to -78 °C on a dry ice/acetone bath for 30 min before the solution containing **18** was added fast via cannulation. After 1 h, the dry ice/acetone bath was removed, and the reaction mixture was allowed to reach room temperature for 3 h. The reaction was quenched with aqueous NH_4Cl (sat., 20 mL), washed with H_2O (30 mL) and extracted with CH_2Cl_2 (3 x 50 mL). The combined organic phase was dried over $MgSO_4$ and concentrated *in vacuo*. The product was purified by flash column chromatography (SiO_2, 50% CH_2Cl_2/heptane) and recrystallized from CH_2Cl_2/heptane. This yielded compound **3a** (47 mg, 0.0267 mmol, 18% over two steps) as a red solid. ^{1}H NMR (500 MHz, $CDCl_3$) δ 7.93 (s, 2H), 7.91 (s, 4H), 7.79 (d, *J* = 7.2 Hz, 2H), 7.61 (d, *J* = 7.5 Hz, 2H), 7.31 (t, *J* = 7.5, 2H), 7.27 (t, *J* = 7.2 Hz, 2H), 3.10 – 2.96 (m, 16H), 1.83 – 1.70 (m, 16H), 1.53 – 1.44 (m, 16H), 1.39 – 1.33 (m, 16H), 1.33 – 1.22 (m, 16H), 0.95 – 0.87 (m, 12H), 0.86 – 0.83 (m, 12H). ^{13}C NMR (126 MHz, $CDCl_3$) δ 138.77, 138.12, 137.76, 137.65, 137.01, 136.31, 135.71, 135.62, 135.47, 135.09, 130.01, 129.59, 128.37, 127.56, 126.37, 125.46, 122.99, 121.72, 121.38, 119.34, 114.13, 113.93, 113.82, 37.08, 36.90, 36.67, 31.67, 31.62, 31.56, 30.16, 30.14, 30.10, 30.02, 28.64, 28.56, 28.53, 22.79, 22.78, 22.75, 14.22, 14.21, 14.20. 5 sp^3-carbon signals missing presumably due to overlap. HRMS (MALDI$^+$ FT-ICR, dithranol): *m/z* = 1759.4714 $[M]^{\bullet+}$, calcd. for $[C_{94}H_{118}S_{16}^{\bullet+}]$ *m/z* = 1759.4793.

Compound **3b**

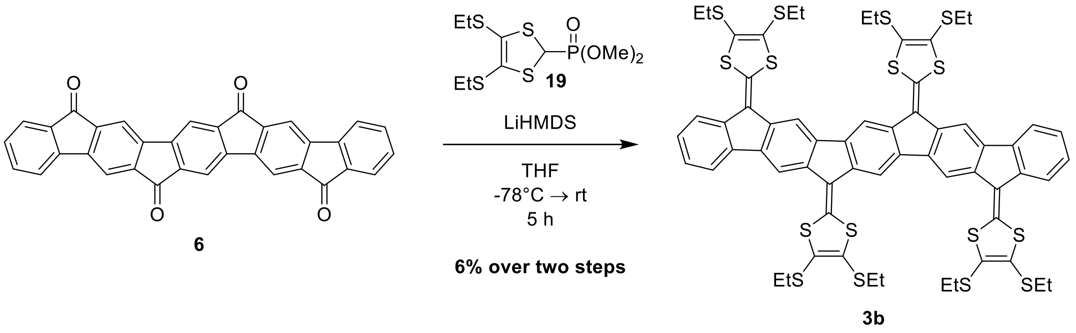

To a flame-dried Schlenk flask under a positive flow of N_2 were added phosphonate ester **19**[47] (477 mg, 1.43 mmol) and anh. THF (10 mL). The solution was degassed and cooled to -78 °C on a dry ice/acetone bath for 30 min. LiHMDS (1 M in toluene, 1.5 mL, 1.50 mmol) was added dropwise, and the reaction mixture was stirred at -78 °C for 1 h. In a separate flame-dried Schlenk flask were added compound **6** (70 mg) and anh. THF (10 mL). The suspension was degassed and cooled to -78 °C on a dry ice/acetone bath for 30 min before the solution containing **19** was added fast via cannulation. After 1 h, the dry ice/acetone bath was removed, and the reaction mixture was allowed to reach room temperature for 3.5 h. The reaction was quenched with aqueous NH_4Cl (sat., 20 mL), washed with H_2O (30 mL) and extracted with CH_2Cl_2 (3 x 60 mL). The combined organic phase was dried over $MgSO_4$ and concentrated *in vacuo*. The product was recrystallized from CH_2Cl_2/heptane, followed by trituration with pentane and MeOH. This yielded compound **3b** (14 mg, 10.7 µmol, 6% over two steps) as a red solid. ^{1}H NMR (500 MHz, CS_2 with DMSO-d_6 lock tube) δ 7.80 (s, 4H), 7.70 (s, 2H), 7.57 – 7.52 (m, 2H), 7.38 – 7.35 (m, 2H), 7.08 – 7.00 (m, 4H), 2.89 – 2.75 (m, 16H), 1.32 – 1.17 (m, 24H). Residual CH_2Cl_2 (5.03 ppm) present in spectrum from recrystallization. Due to low solubility of compound **3b**, it was not possible to obtain ^{13}C NMR resonances. HRMS (MALDI$^+$ FT-ICR, dithranol): *m/z* = 1309.9751 $[M]^{\bullet+}$, calcd. for $[C_{62}H_{54}S_{16}^{\bullet+}]$ *m/z* = 1309.9752.

Compound **4**

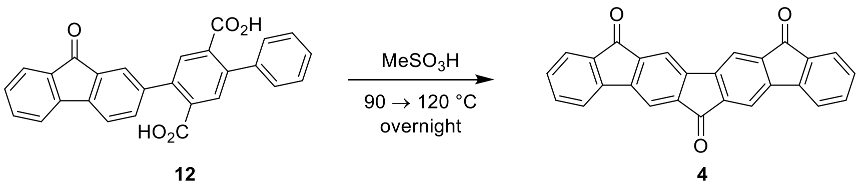

$MeSO_3H$ was heated to 90 °C, and compound **12** (648 mg, 1.54 mmol) was added in small portions over 30 min. The reaction mixture was heated to 120 °C and stirred overnight. The reaction mixture was cooled to room temperature and poured onto ice resulting in precipitation of a dark solid. The suspension was stirred for 10 min, and the solids were collected by filtration and washed extensively with H_2O, EtOAc, and THF. The product was dried under N_2 yielding compound **4** (500 mg) as a dark green solid. The product was used without further purification. HRMS (MALDI$^+$ FT-ICR, dithranol): *m/z* = 385.0872 $[M+H]^+$, calcd. for $[C_{27}H_{13}O_3^+]$ *m/z* = 385.0860.

Compound **5**

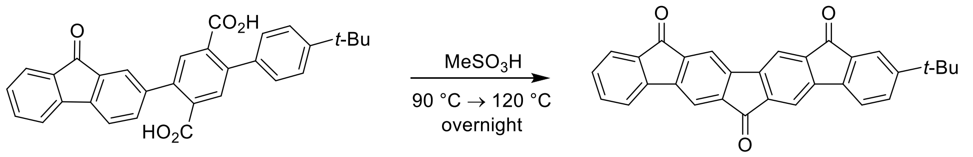

13 **5**

$MeSO_3H$ (20 mL) was heated to 90 °C, and compound **13** (298 mg, 0.625 mmol) was added in small portions over 1 h. The solution was heated to 120 °C, and the reaction mixture was left stirring overnight. The reaction mixture was poured onto ice and stirred for 10 min. The ice was allowed to melt while a black precipitate formed. The solids were collected by filtration and washed extensively with H_2O and EtOAc. The product was dried under N_2 yielding compound **5** (260 mg) as a black solid. The product was used without further purification. HRMS ($MALDI^+$ FT-ICR, dithranol): *m/z* = 463.1704 $[M+Na]^+$, calcd. for $[C_{31}H_{20}O_3Na^{\bullet +}]$ *m/z* = 463.1305.

Compound **6**

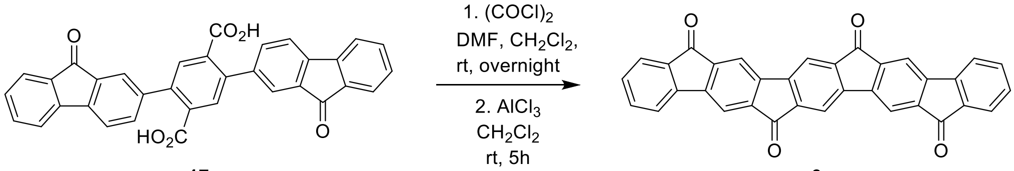

17 **6**

To a flame-dried Schlenk flask was added compound **17** (200 mg, 0.383 mmol) in degassed anh. CH_2Cl_2 (12 mL). Degassed anh. DMF (3 drops) was added, and the suspension was degassed for 10 min. Then $(COCl)_2$ (1.20 mL, 14.2 mmol) was added dropwise, and the reaction mixture was stirred at rt overnight under N_2. The volatiles were removed *in vacuo*, and the flask was evacuated and backfilled with N_2 three times. Anh. CH_2Cl_2 (12 mL) was added, and the solution was degassed for 10 min before $AlCl_3$ (221 mg, 1.66 mmol) was added. The reaction mixture was stirred at rt for 5 h and poured into a 1:1 mixture of ice and aq. HCl (conc., 10 mL) resulting in precipitating of a dark solid. The slurry was stirred for 10 min, and the precipitate was collected by filtration and washed with H_2O, MeOH, THF, and CH_2Cl_2. This yielded compound **6** (177 mg) as a black solid. The product was used without further purification. HRMS ($MALDI^+$ FT-ICR, dithranol): *m/z* = 487.0971 $[M + H]^+$, calcd. for $[C_{34}H_{15}O_4^+]$ *m/z* = 487.0965.

Compound **10**

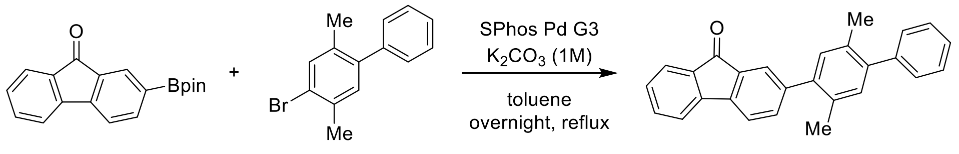

To a solution of compound **9**[44] (800 mg, 2.61 mmol) and compound **7**[42] (820 mg, 3.14 mmol) in toluene (30 mL) was added SPhos Pd G3 (102 mg, 5 mol%), and the solution was degassed for 20 min. Degassed aq. K_2CO_3 (1 M, 10.0 mL, 10.0 mmol) was added, and the reaction mixture was heated to reflux overnight under N_2. The reaction mixture was diluted with H_2O (100 mL) and extracted with CH_2Cl_2 (3 x 70 mL). The combined organic phase was dried over $MgSO_4$ and concentrated *in vacuo.* The product was purified by flash column chromatography (SiO_2, 10 – 20% EtOAc/heptane), which yielded compound **10** (766 mg, 2.13 mmol, 81%) as a yellow solid. HRMS (ESP^+): *m/z* = 361.1601 $[M + H]^+$, calcd. for $[C_{27}H_{21}O^+]$ *m/z* = 361.1587. ^{1}H NMR (500 MHz, $CDCl_3$) δ 7.70 – 7.68 (m, 2H), 7.59 (d, *J* = 7.6, 1H), 7.57 (d, *J* = 7.4, 1H), 7.53 – 7.40 (m, 2H), 7.45 – 7.42 (m, 2H), 7.39 – 7.36 (m, 3H), 7.33 – 7.30 (m, Hz, 1H), 7.17 (s, 2H), 2.31 (s, 3H), 2.29 (s, 3H). ^{13}C NMR (126 MHz, $CDCl_3$) δ 194.07, 144.54, 143.04, 141.65, 141.56, 139.62, 135.61, 134.93, 134.61, 134.39, 133.07, 132.60, 132.24, 131.64, 129.34, 129.31, 129.15, 128.26, 127.01, 125.33, 124.53, 120.48, 120.23, 20.06. 1 sp^3-carbon signal missing presumably due to overlap HRMS (ESP^+): *m/z* = 361.1601 $[M + H]^+$, calcd. for $[C_{27}H_{21}O^+]$ *m/z* = 361.1587.

Compound **11**

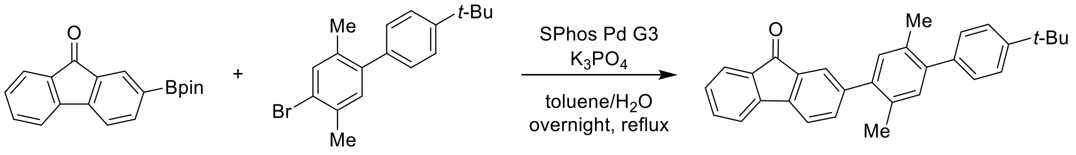

To a solution of compound **9**[44] (306 mg, 1.00 mmol) and **8**[43] (479 mg, 1.51 mmol) in toluene (10 mL) were added K_3PO_4 (832 mg, 3.92 mmol) and H_2O (3 mL). The solution was degassed with Ar for 10 min. SPhos Pd G3 (40 mg, 5 mol%) was added, and the solution was further degassed for 10 min. The reaction mixture was heated to reflux and stirred overnight under N_2, after which it was cooled to room temperature. The reaction mixture was diluted with H_2O (50 mL) and extracted with EtOAc (3 x 50 mL). The combined organic phase was dried over $MgSO_4$ and concentrated *in vacuo*. The product was purified by flash column chromatography (SiO_2, 5 – 10% EtOAc/heptane) followed by recrystallization from heptane. This yielded compound **11** (307 mg, 0.737 mmol, 75%) as a yellow powder. ^{1}H NMR (500 MHz, $CDCl_3$) δ 7.70 – 7.67 (m, 2H), 7.58 (d, *J* = 7.7 Hz, 1H), 7.56 (d, *J* = 7.4f Hz, 1H), 7.53 – 7.49 (m, 2H), 7.44 (d, *J* = 8.2 Hz, 2H), 7.33 – 7.29 (m, 1H), 7.31 (d, *J* = 8.2 Hz, 2H), 7.18 (s, 1H), 7.16(s, 1H), 2.31 (s, 6H), 1.38 (s, 9H). ^{13}C NMR (126 MHz, $CDCl_3$) δ 193.95, 149.71, 144.42, 142.97, 142.87, 141.33, 139.26, 138.46, 135.48, 134.78, 134.25, 133.03, 132.40, 132.23, 131.50, 129.00, 128.83, 125.21, 125.01, 124.39, 120.32, 120.07, 34.57, 31.43, 20.01, 19.92; 1 sp^2-carbon signal missing presumably due to overlap. HRMS (ESP^+): *m/z* = 417.2224 $[M + H]^+$, calcd. for $[C_{31}H_{29}O^+]$ *m/z* = 417.2213.

Compound **12**

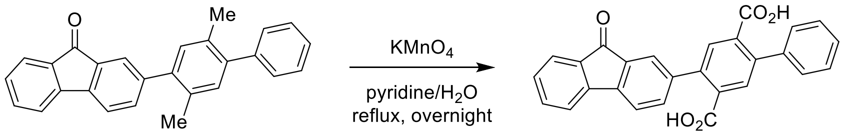

To a solution of compound **10** (752 mg, 2.09 mmol) in pyridine (40 mL) were added $KMnO_4$ (2.95 g, 18.7 mmol) and H_2O (10 mL), and the reaction mixture was heated to reflux. Four portions of $KMnO_4$ (an average of 1.25 g, 7.91 mmol) and H_2O (3 x 10 mL, 1 x 40 mL) were added over 2 h. The reaction mixture was stirred at vigorous reflux overnight, filtered hot, and washed with boiling H_2O. The filtrate was acidified with aq. HCl (conc.) until pH =1, resulting in precipitation of a yellow solid. The product was collected by filtration and washed with H_2O and dried under N_2. This yielded compound **12** (698 mg, 1.66 mmol, 79%) as a yellow solid. HRMS (ESP$^+$): *m/z* = 421.1083 [M + H]$^+$, calcd. for [$C_{27}H_{17}O_5^+$] *m/z* = 421.1071. ^{1}H NMR (500 MHz, DMSO-*d*$_6$) δ 13.23 (s, 2H), 7.89 (d, *J* = 7.7 Hz, 1H), 7.87 (d, *J* = 7.6 Hz, 1H), 7.77 (s, 1H), 7.76 (s, 1H), 7.68 – 7.65 (m, 3H), 7.59 (s, 1H), 7.49 – 7.39 (m, 6H). ^{13}C NMR (126 MHz, DMSO-*d*$_6$) δ 192.87, 168.71, 168.37, 143.62, 143.07, 140.86, 140.02, 139.41, 138.76, 135.57, 135.33, 134.65, 133.53, 133.28, 131.43, 130.83, 129.63, 128.36, 128.27, 127.73, 124.07, 123.78, 121.44, 121.21. 1 sp^2-carbon signal missing presumably due to overlap. HRMS (ESP$^+$): *m/z* = 421.1083 [M + H]$^+$, calcd. for [$C_{27}H_{17}O_5^+$] *m/z* = 421.1071.

Compound **13**

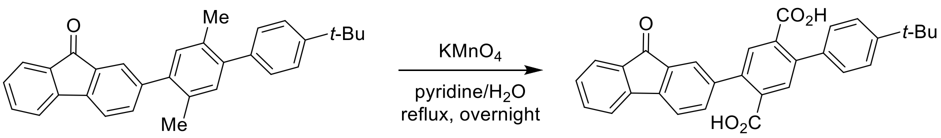

To a solution of compound **11** (498 mg, 1.20 mmol) in pyridine (25 mL) were added $KMnO_4$ (1.79 mg, 11.4 mmol) and H_2O (3 mL), and the reaction mixture was heated to reflux. Four portions of $KMnO_4$ (an average of 755 mg, 4.78 mmol) and H_2O (3 x 7 mL, 1 x 25 mL) were added to the reaction mixture over 2 h. The reaction mixture was stirred at vigorous reflux overnight, filtered hot, and washed with boiling H_2O. After cooling to room temperature, the solution was washed with EtOAc (2 x 100 mL) and extracted with H_2O (2 x 50 mL). The combined aqueous phase was acidified with aq. HCl (conc.) until pH 1, and the solution was extracted with EtOAc (3 x 300 mL). The combined organic phase was dried over $MgSO_4$ and concentrated *in vacuo*, yielding compound **13** (527 mg, 1.11 mmol, 91%) as a white solid. ^{1}H NMR (500 MHz, DMSO-*d*$_6$) δ 13.17 (s, 2H), 7.89 (d, *J* = 7.7 Hz, 1H), 7.88 – 7.86 (m, 1H), 7.77 (s, 1H), 7.72 (s, 1H), 7.68 – 7.64 (m, 3H), 7.59 (s, 1H), 7.49 (d, *J* = 8.5 Hz, 3H), 7.42 (t, *J* = 7.7 Hz, 1H), 7.37 (d, *J* = 8.5 Hz, 2H), 1.33 (s, 9H). ^{13}C NMR (126 MHz, DMSO-*d*$_6$) δ 192.88, 168.91, 168.41, 150.14, 143.62, 143.05, 140.89, 139.66, 138.53, 136.39, 135.57, 135.33, 134.78, 133.51, 133.28, 131.36, 130.63, 129.62, 127.99, 125.23, 124.07, 123.79, 121.43, 121.21, 34.35, 31.14; 1 sp^2-carbon signal missing presumably due to overlap. HRMS (ESP$^+$): *m/z* = 477.1697 [M + H]$^+$, calcd. for [$C_{31}H_{25}O_5^+$] *m/z* = 477.1697.

Compound **16**

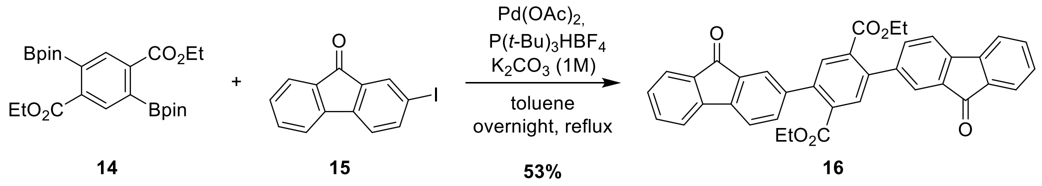

To a N_2 purged flask were added **14**[45] (710 mg, 1.50 mmol), **15**[46] (963 mg, 3.14 mmol), $Pd(OAc)_2$ (63 mg, 20 mol%), Pd(*t*-Bu)$_3$HBF$_4$ (177 mg, 40 mol%) and toluene (50 mL). The solution was degassed for 20 min, and degassed aq. K_2CO_3 (1 M, 8.80 mL, 8.80 mml) was added. The reaction mixture was heated to reflux and stirred overnight under N_2. The reaction mixture was cooled to room temperature, diluted with CH_2Cl_2 (100 mL), washed with H_2O (100 mL), and extracted with CH_2Cl_2 (3 x 60 mL). The combined organic phase was dried over $MgSO_4$ and concentrated *in vacuo*. The product was recrystallized from EtOAc/heptane and washed with heptane. This yielded compound **16** (455 mg, 0.786 mmol, 53%) as a yellow solid. ^{1}H NMR (500 MHz, $CDCl_3$) δ 7.90 (s, 2H), 7.72 – 7.68 (m, 4H), 7.61 – 7.56 (m, 4H), 7.53 (td, *J* = 7.4, 1.2 Hz, 2H), 7.50 (dd, *J* = 7.6, 1.7 Hz, 1H), 7.33 (td, *J* = 7.4, 1.2 Hz, 1H), 4.20 (q, *J* = 7.1 Hz, 4H), 1.14 (t, *J* = 7.1 Hz, 6H). ^{13}C NMR (126 MHz, $CDCl_3$) δ 193.66, 167.19, 144.28, 143.91, 141.26, 140.82, 135.02, 134.91, 134.59, 134.43, 133.42, 132.33, 129.43, 124.64, 124.63, 120.70, 120.25, 61.82, 14.06. HRMS (ESP$^+$): *m/z* = 579.1809 [M + H]$^+$, calcd. for [$C_{38}H_{27}O_6^+$] *m/z* = 579.1803.

Compound **17**

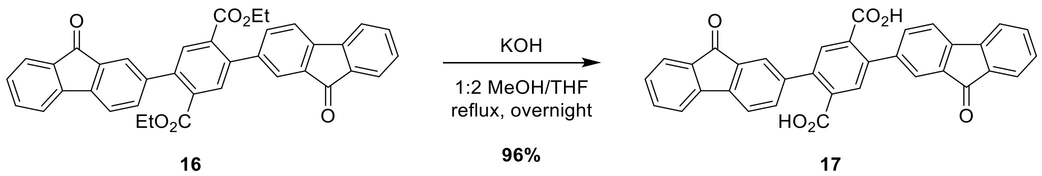

To a solution of compound **16** (184 mg, 0.32 mmol) in a 1:2 mixture of MeOH (10 mL) and THF (20 mL) was added a solution of KOH (418 mg, 7.45 mmol) in H_2O (4 mL). The reaction mixture was heated to reflux and stirred overnight before it was cooled to room temperature. The reaction mixture was diluted with H_2O (150 mL) and washed with CH_2Cl_2 (100 mL). The aqueous phase was concentrated *in vacuo* and acidified with aq. HCl (1 M) resulting in precipitation of a yellow solid. The precipitate was collected via filtration and washed with H_2O and MeOH. The collected product was dried under N_2, yielding compound **17** (158 mg, 0.30 mmol, 96%) as a yellow solid. ^{1}H NMR (500 MHz, DMSO-d$_6$) δ 13.35 (s, 2H), 7.91 (d, *J* = 7.7 Hz, 2H), 7.88 (d, *J* = 7.5 Hz, 2H), 7.85 (s, 2H), 7.69 (d, *J* = 7.7 Hz, 2H), 7.66 (m, 4H), 7.61 (s, 2H), 7.42 (t, *J* = 7.5 Hz, 2H). ^{13}C NMR (126 MHz, DMSO-d$_6$) δ 192.84, 168.31, 143.59, 143.16, 140.70, 139.18, 135.58, 135.32, 134.00, 133.50, 133.31, 131.30, 129.65, 124.08, 123.76, 121.47, 121.26. HRMS (ESP$^+$): *m/z* = 545.1009 [M + Na]$^+$, calcd. for [$C_{34}H_{18}NaO_6^+$] *m/z* = 545.0996.

NMR Spectra

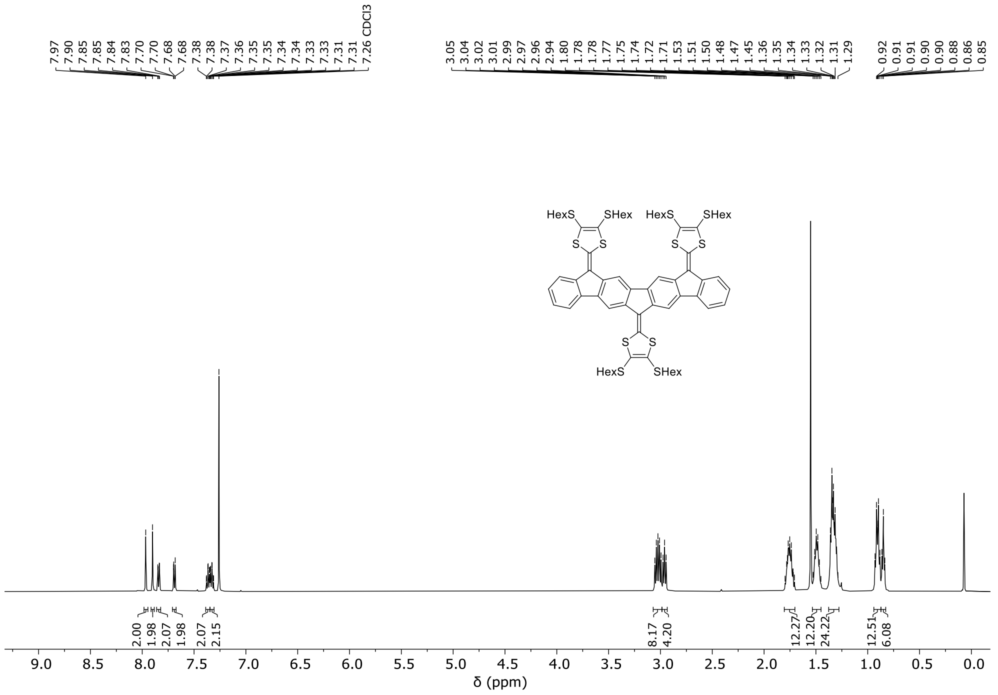

Figure S2. ^{1}H NMR (500 MHz) spectrum of compound **1a** in $CDCl_3$.

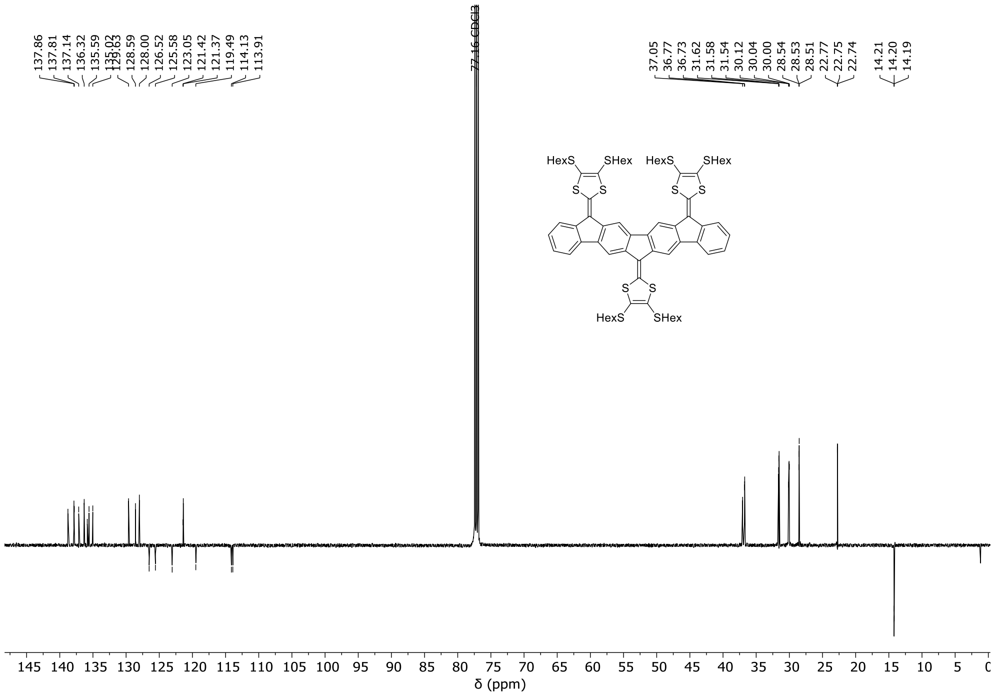

Figure S3. ^{13}C NMR (126 MHz) spectrum of compound **1a** in $CDCl_3$.

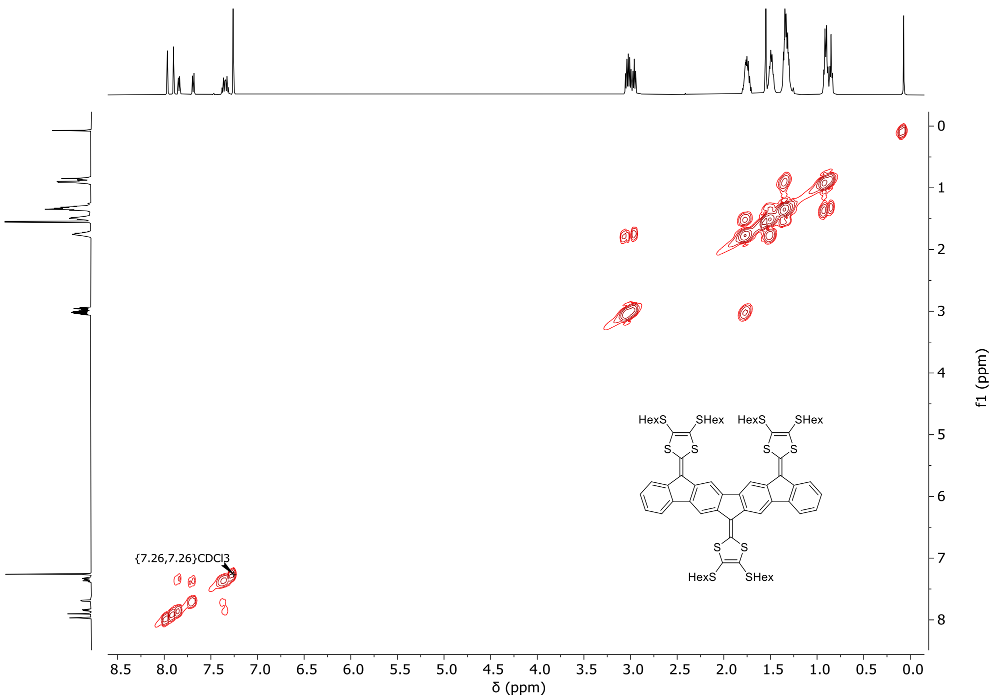

Figure S4. ^{1}H-^{1}H COSY NMR (500 MHz) of compound **1a** in $CDCl_3$.

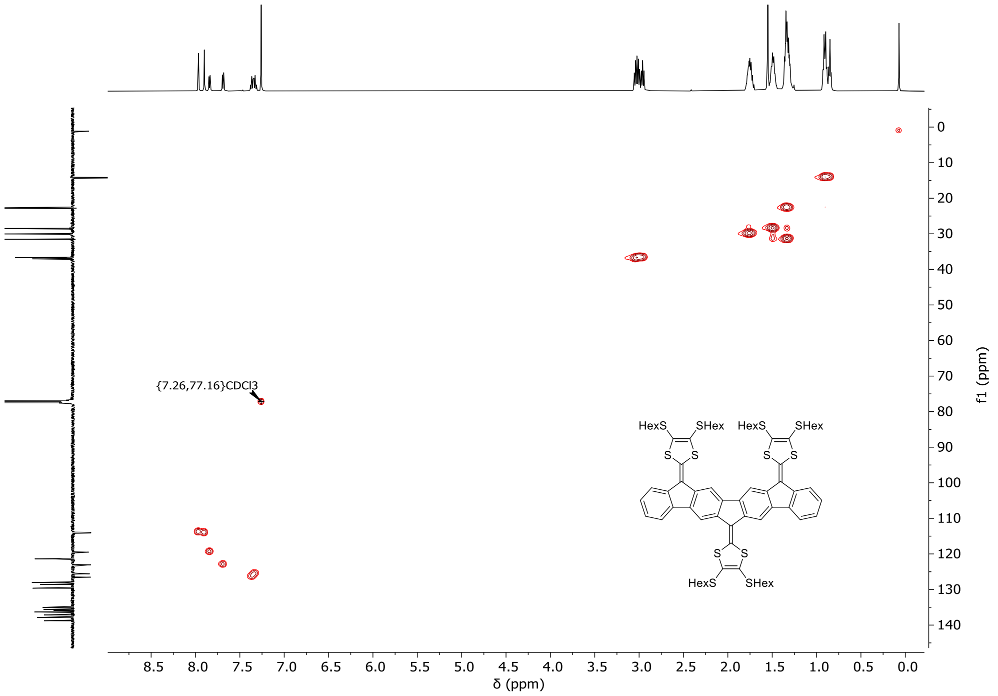

Figure S5. ^{1}H-^{13}C HSQC NMR (500/126 MHz) of compound **1a** in $CDCl_3$.

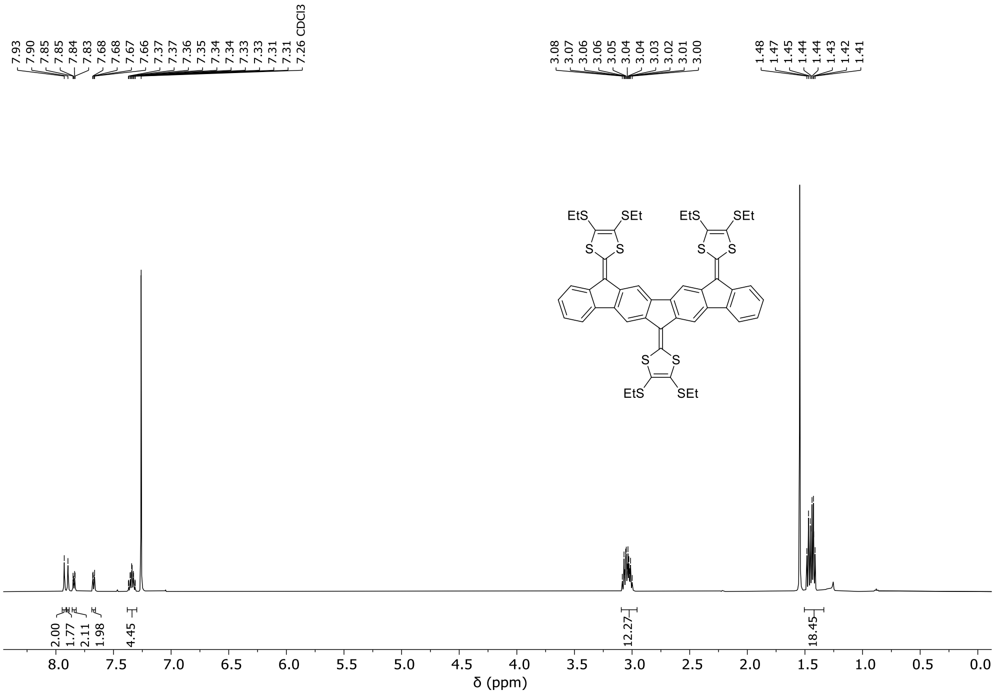

Figure S6. ^{1}H NMR (500 MHz) spectrum of compound **1b** in $CDCl_3$.

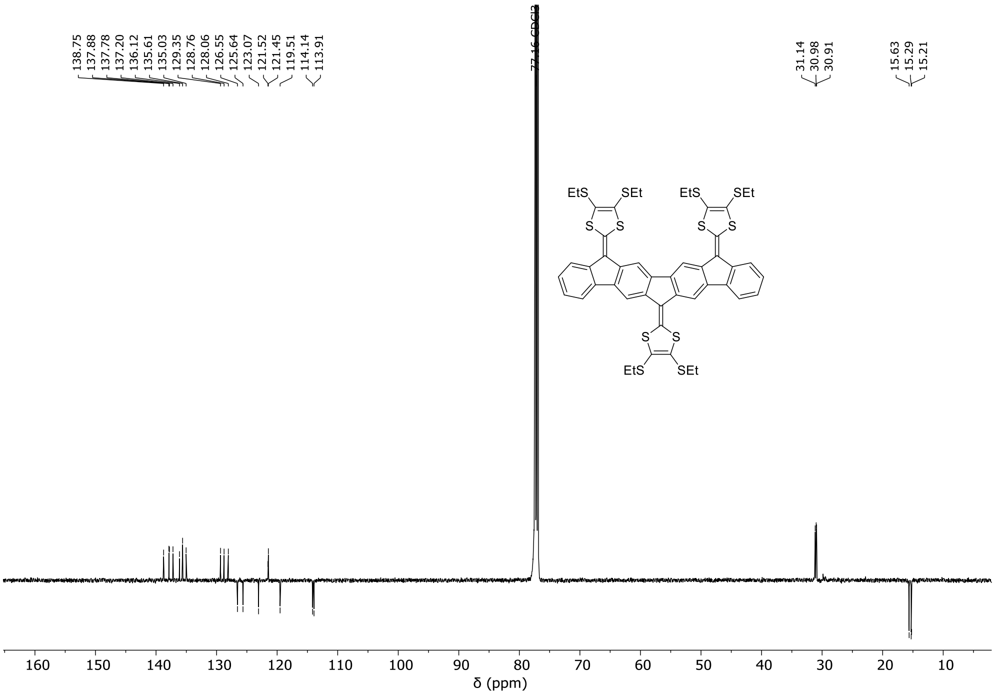

Figure S7. ^{13}C NMR (126 MHz) spectrum of compound **1b** in $CDCl_3$.

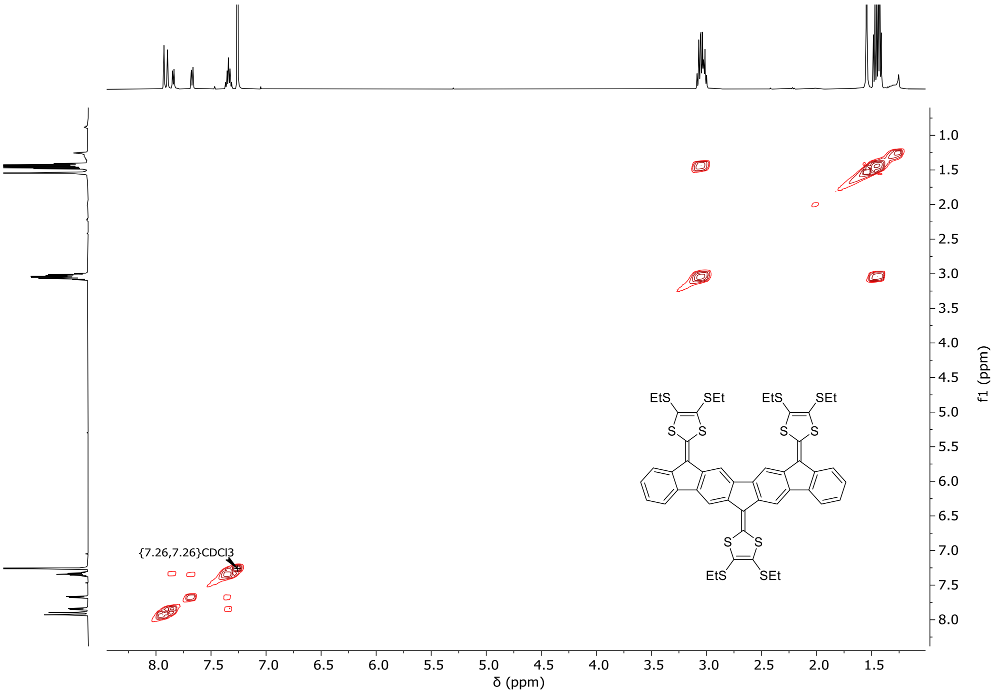

Figure S8. ^{1}H-^{1}H COSY NMR (500 MHz) of compound **1b** in $CDCl_3$.

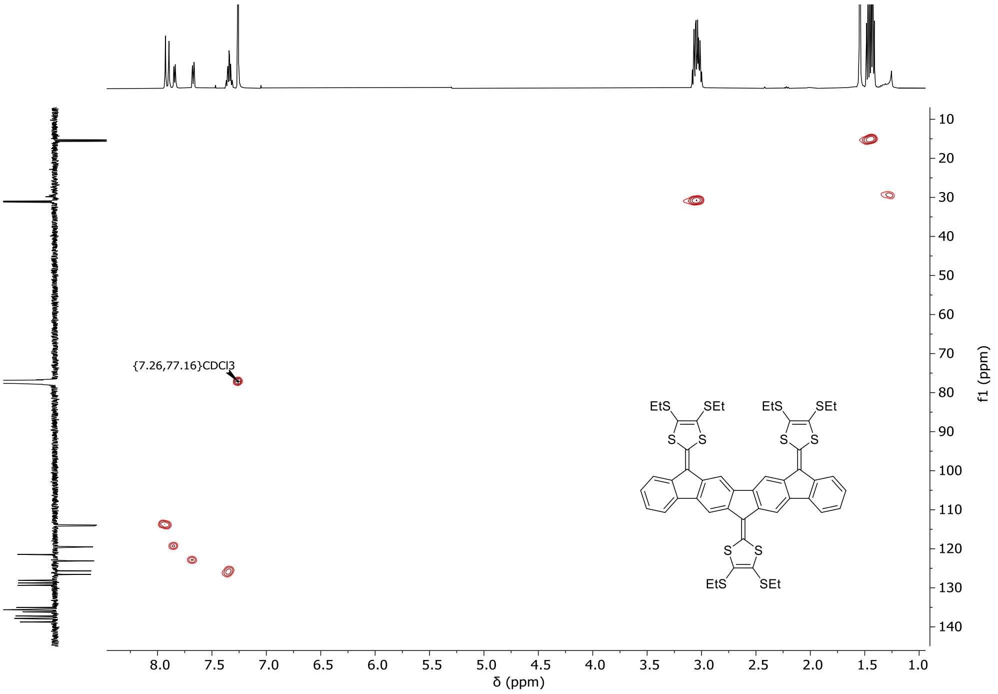

Figure S9. ^{1}H-^{13}C HSQC NMR (500/126 MHz) of compound **1a** in $CDCl_3$.

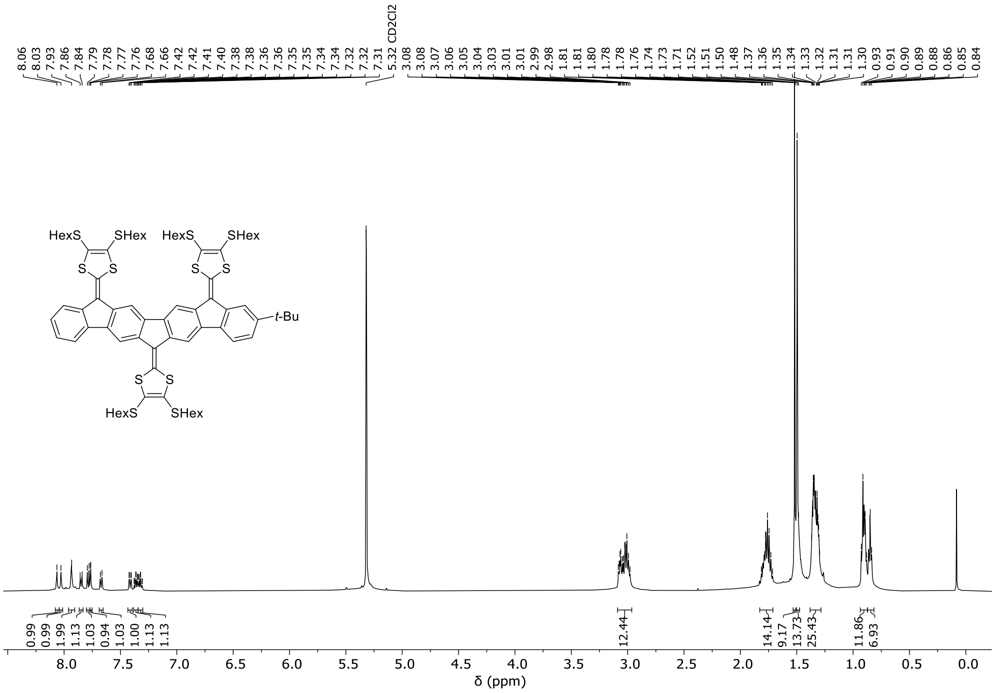

Figure S10. ^{1}H NMR (500 MHz) spectrum of compound **2a** in CD_2Cl_2.

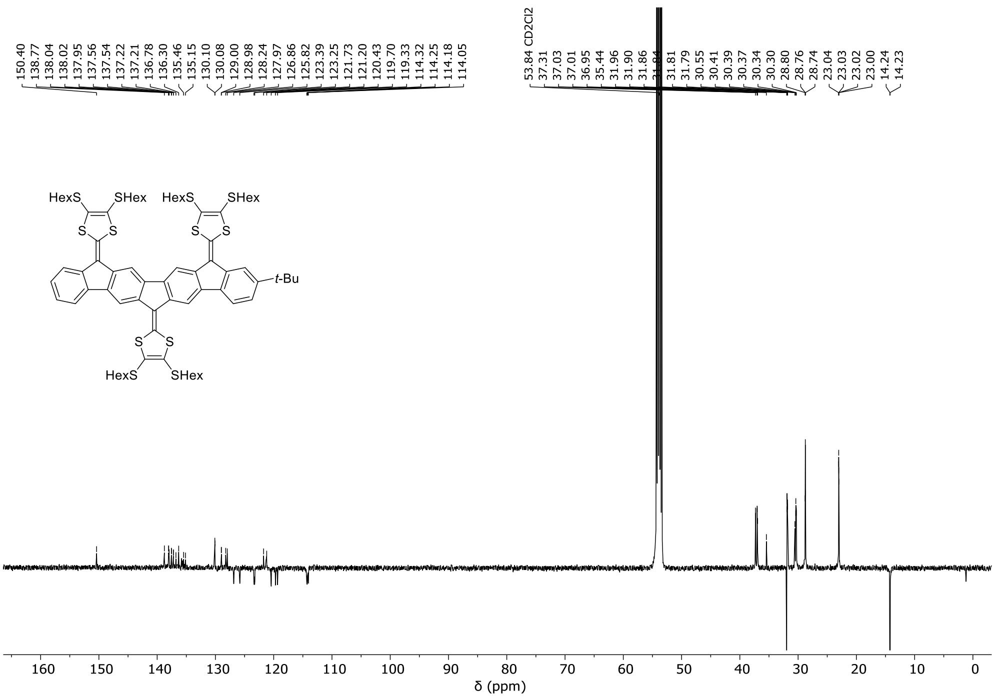

Figure S11. ^{13}C NMR (126 MHz) spectrum of compound **2a** in CD_2Cl_2.

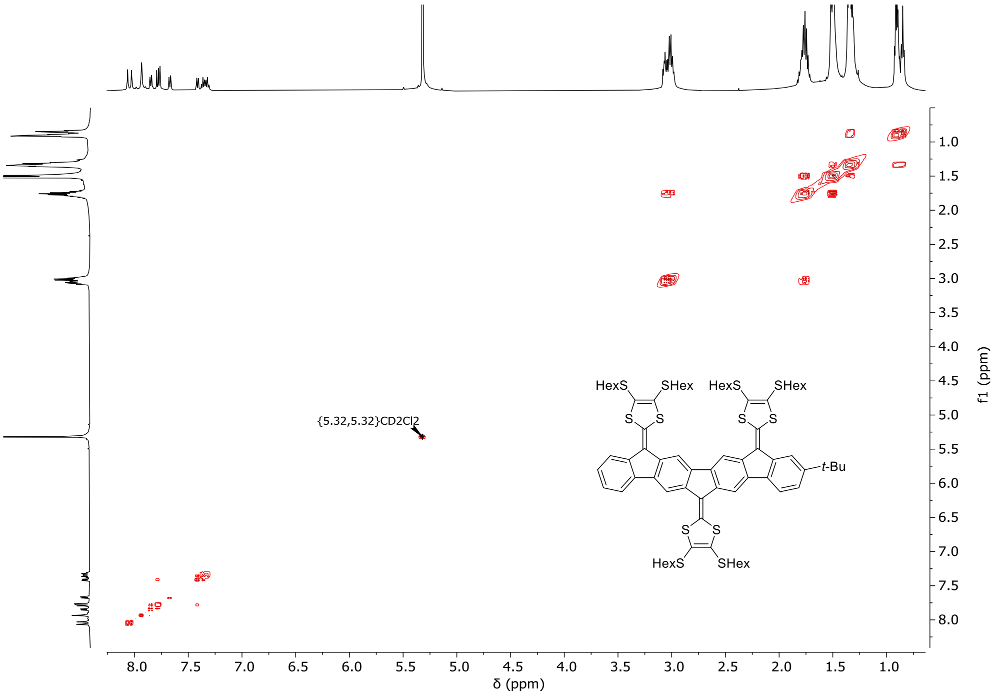

Figure S12. ^{1}H-^{1}H COSY NMR (500 MHz) of compound **2a** in CD_2Cl_2.

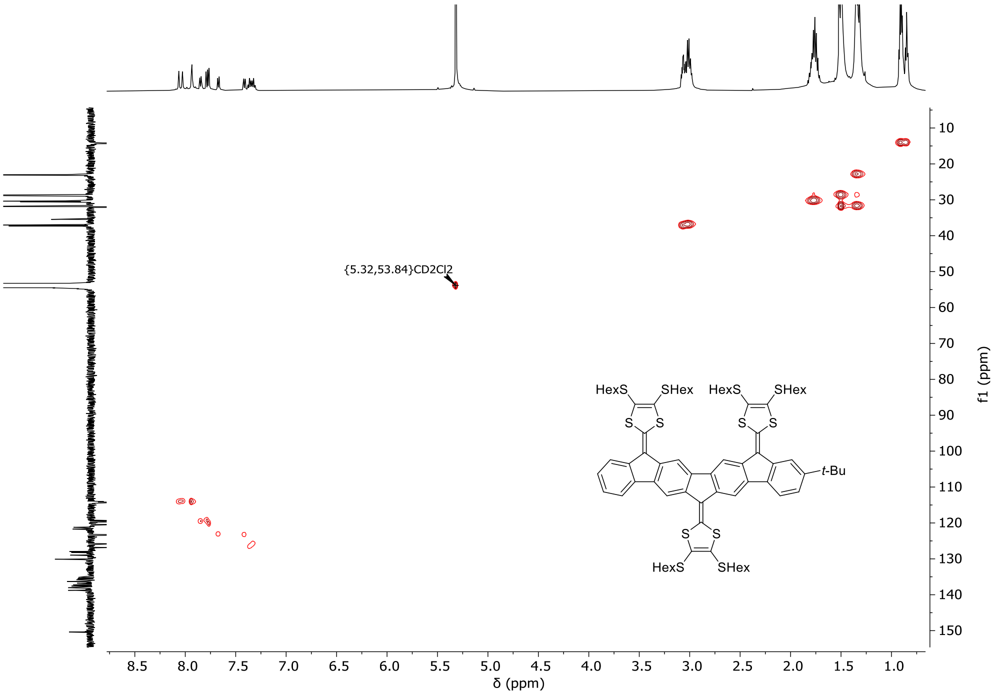

Figure S13. ^{1}H-^{13}C HSQC NMR (500/126 MHz) of compound **2a** in CD_2Cl_2.

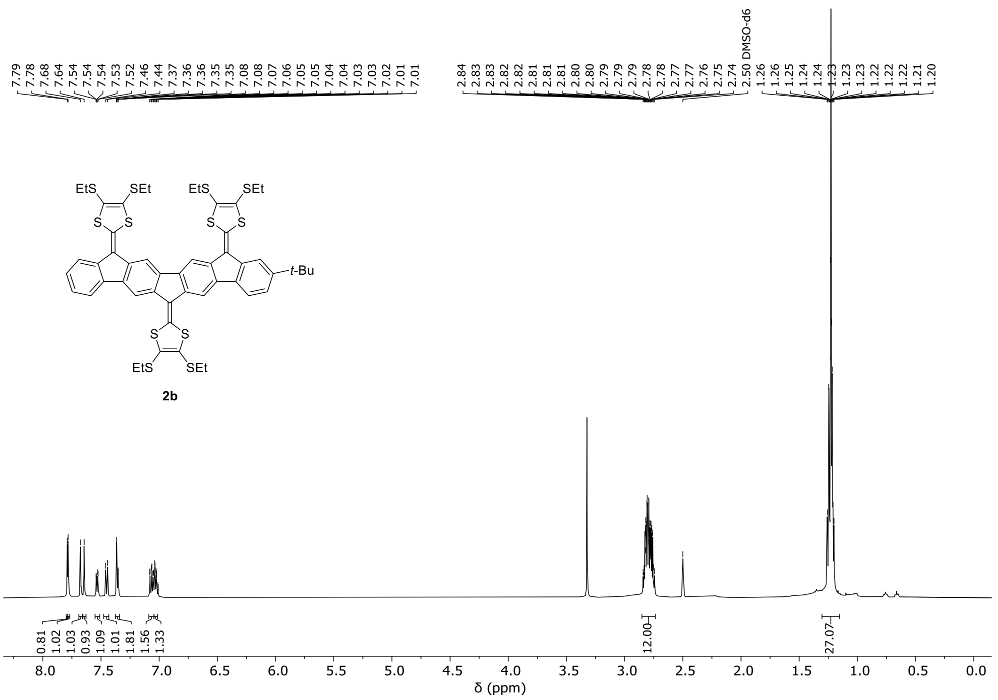

Figure S14. ^{1}H NMR (500 MHz) spectrum of compound **2b** in CS_2 (DMSO-d_6 lock tube).

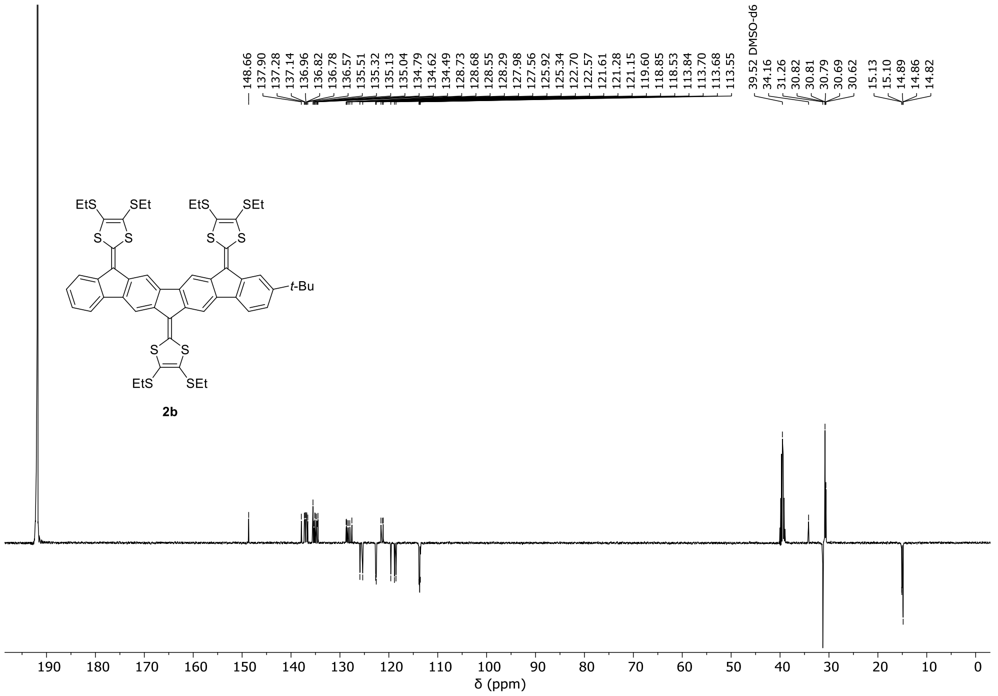

Figure S15. ^{13}C NMR (126 MHz) spectrum of compound **2b** in CS_2 (DMSO-d_6 lock tube).

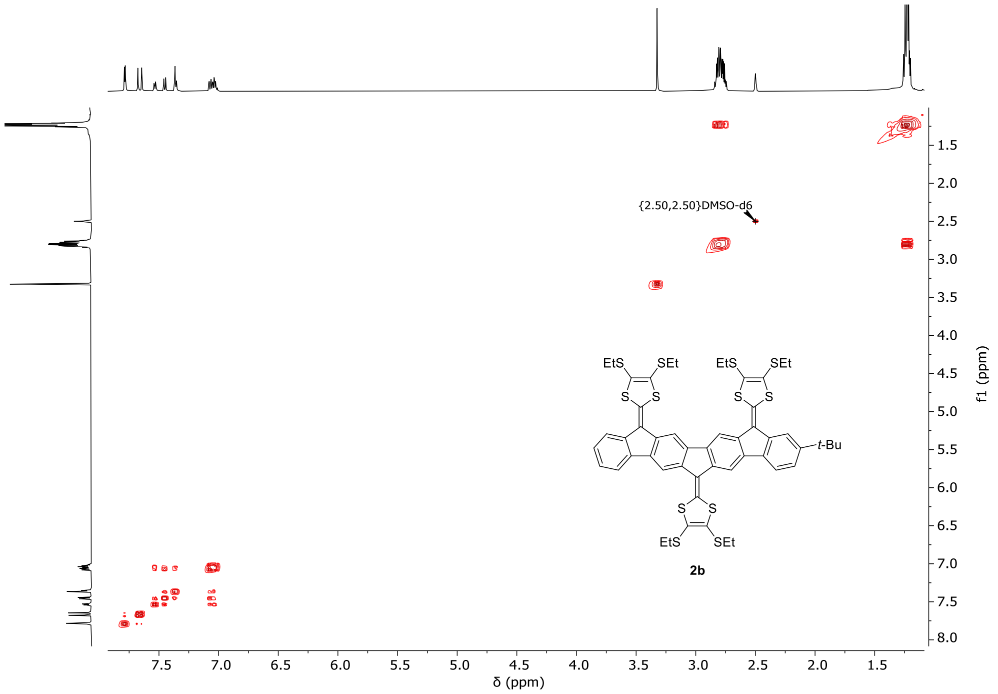

Figure S16. ^{1}H–^{1}H COSY NMR (500 MHz) spectrum of compound **2b** in CS_2 (DMSO-d_6 lock tube).

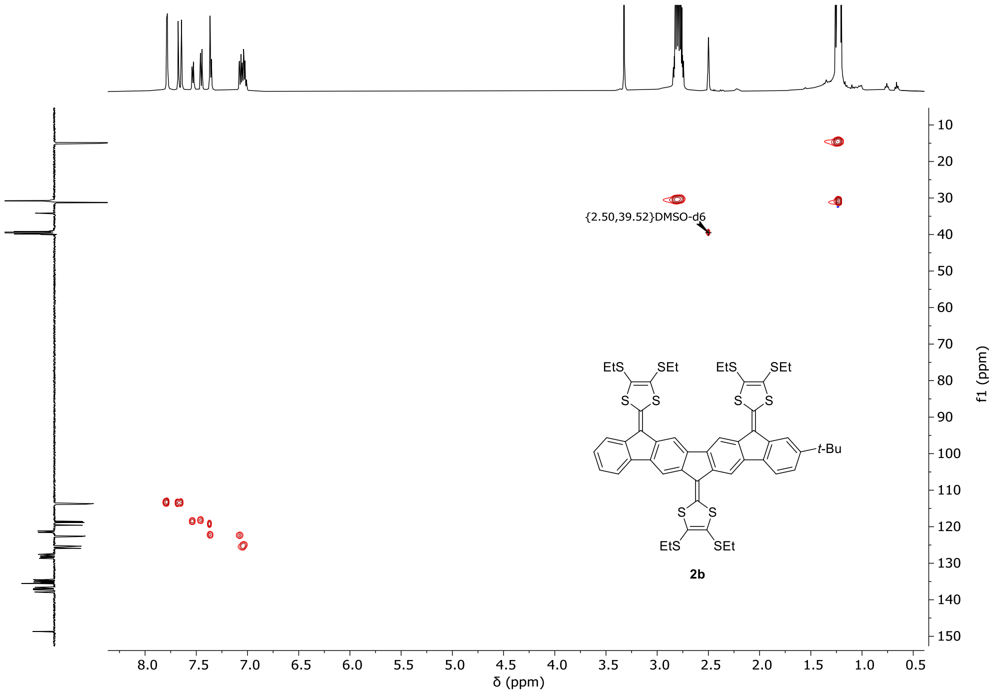

Figure S17. ^{1}H-^{13}C HSQC NMR (500/126 MHz) spectrum of compound **2b** in CS_2 (DMSO-d_6 lock tube).

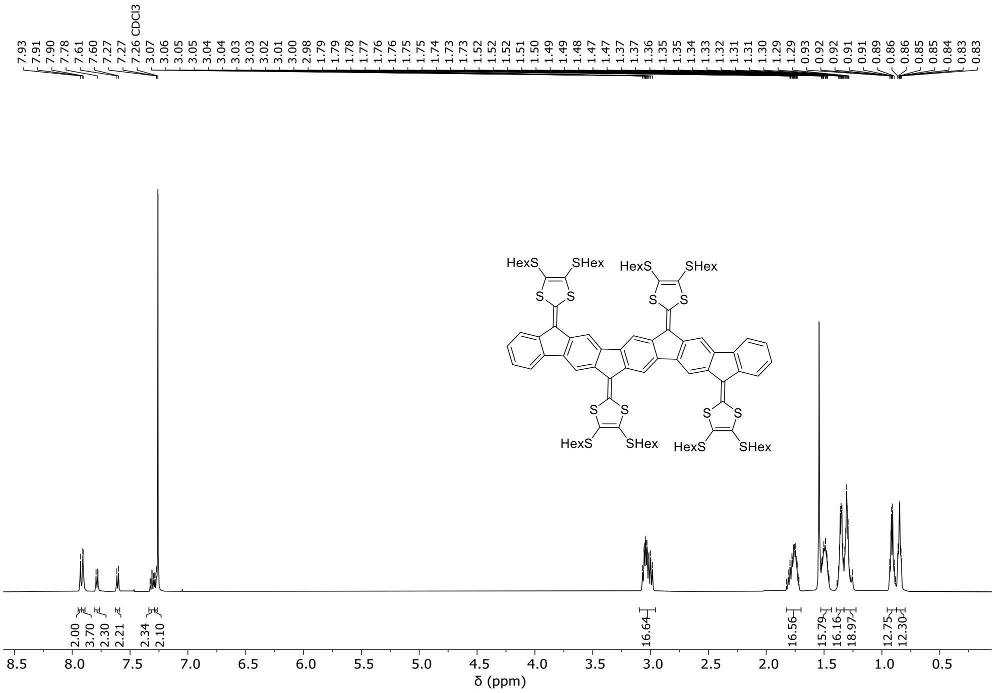

Figure S18. ^{1}H NMR (500 MHz) spectrum of compound **3a** in $CDCl_3$.

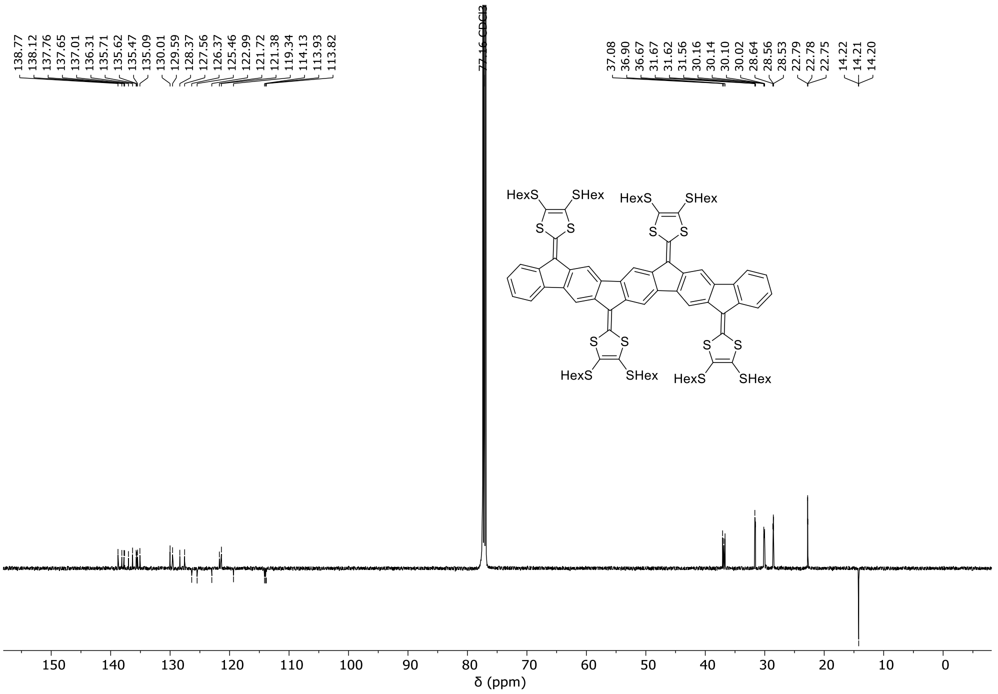

Figure S19. ^{13}C NMR (126 MHz) spectrum of compound **3a** in $CDCl_3$.

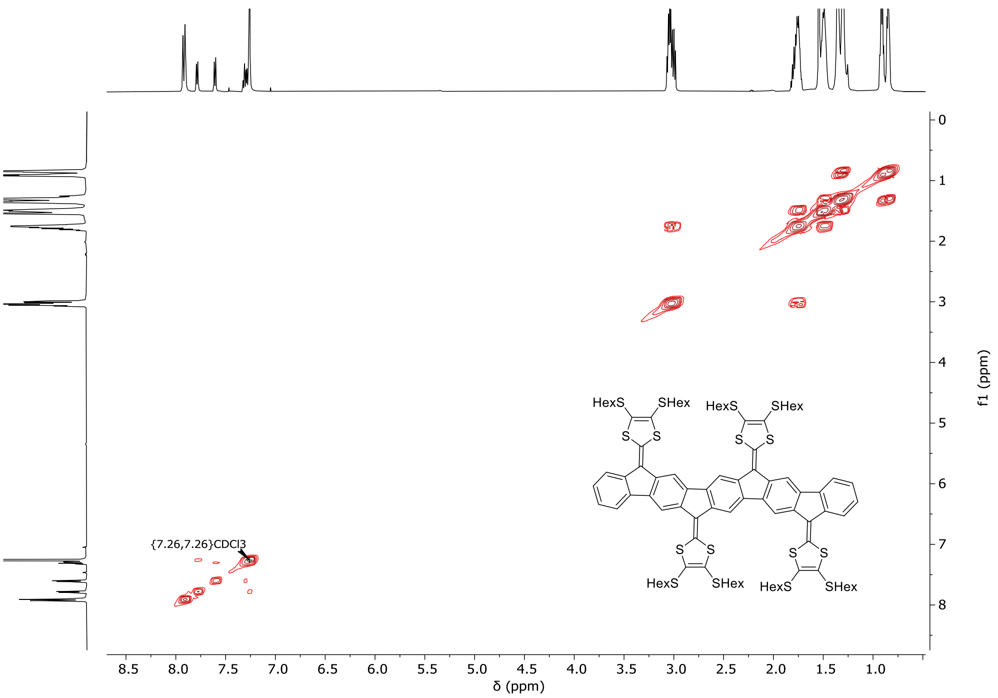

Figure S20. ^{1}H-^{1}H COSY NMR (500 MHz) of compound **3a** in $CDCl_3$.

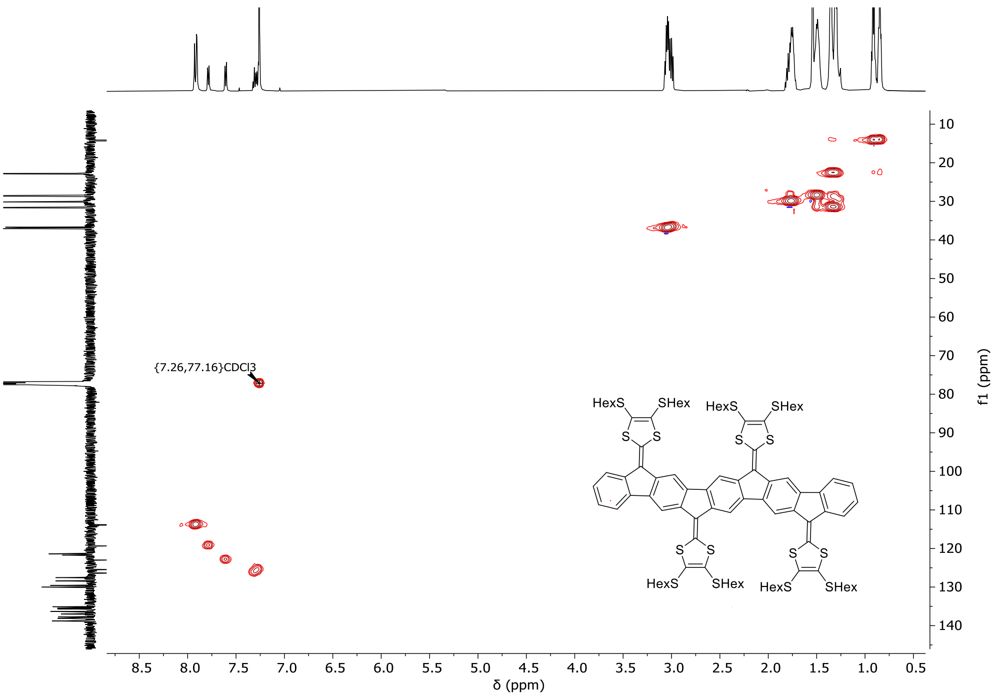

Figure S21. ^{1}H-^{13}C HSQC NMR (500/126 MHz) of compound **3a** in $CDCl_3$.

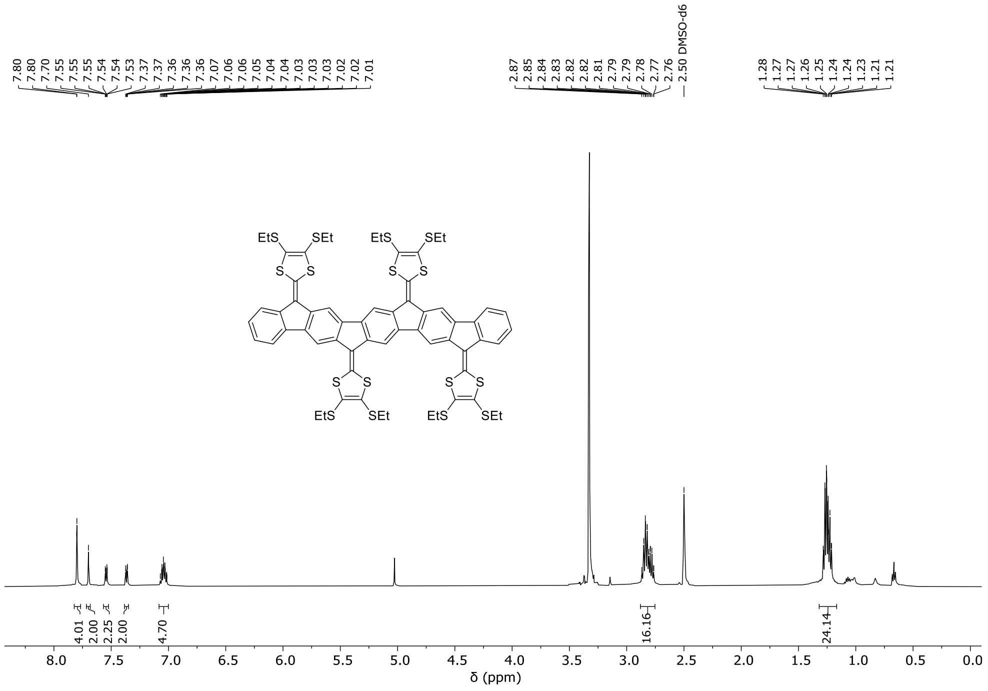

Figure S22. ^{1}H NMR (500 MHz) spectrum of compound **3b** in CS_2 (DMSO-d_6 lock tube).

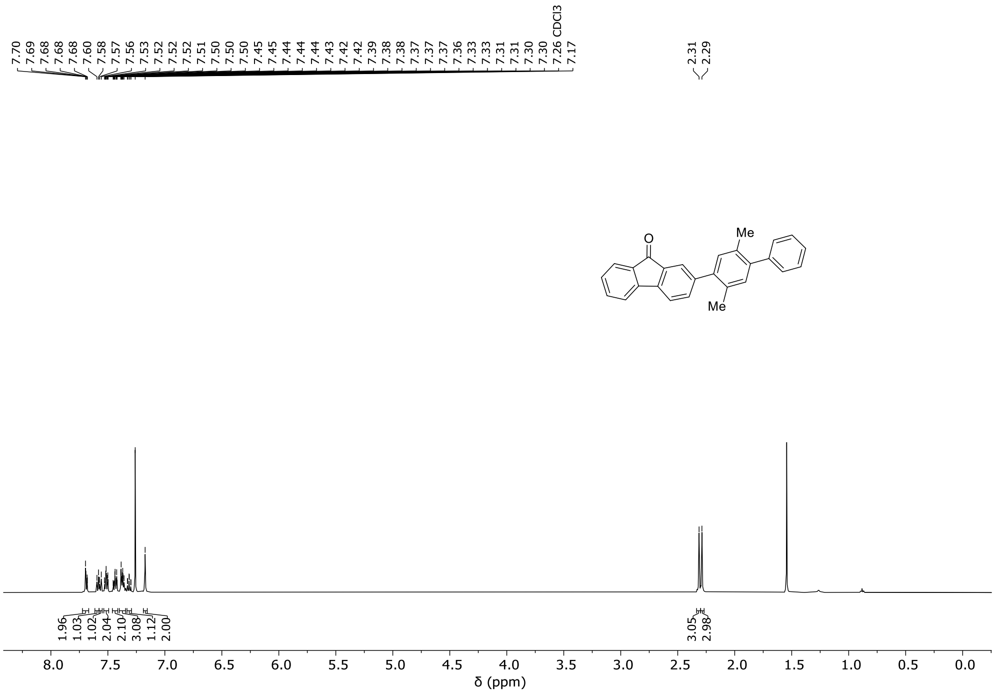

Figure S23. ^{1}H NMR (500 MHz) spectrum of compound **10** in $CDCl_3$.

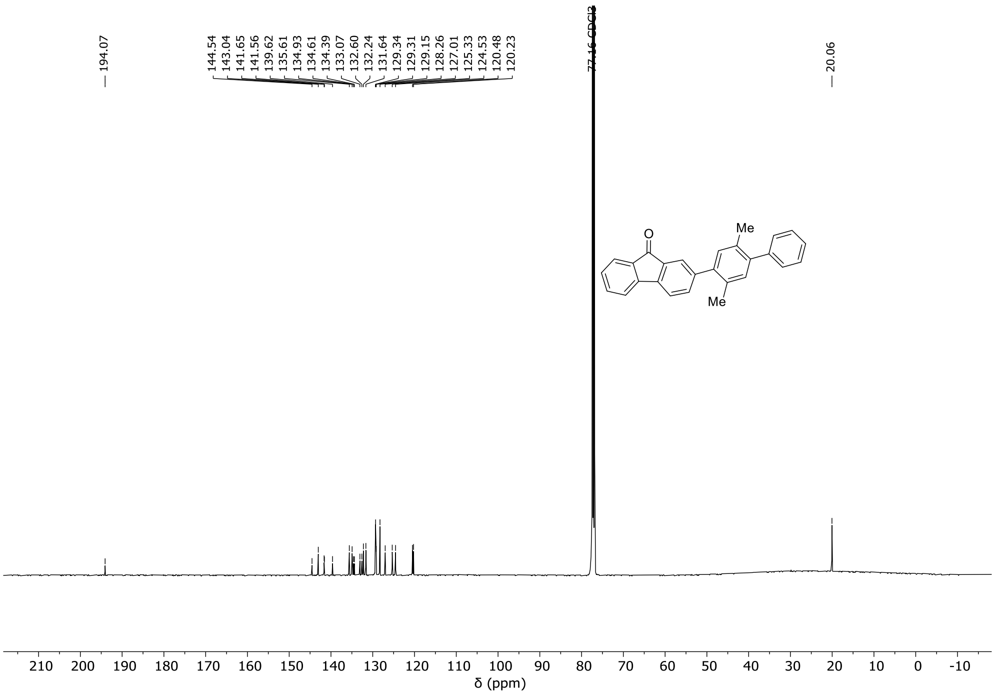

Figure S24. ^{13}C NMR (126 MHz) spectrum of compound **10** in $CDCl_3$.

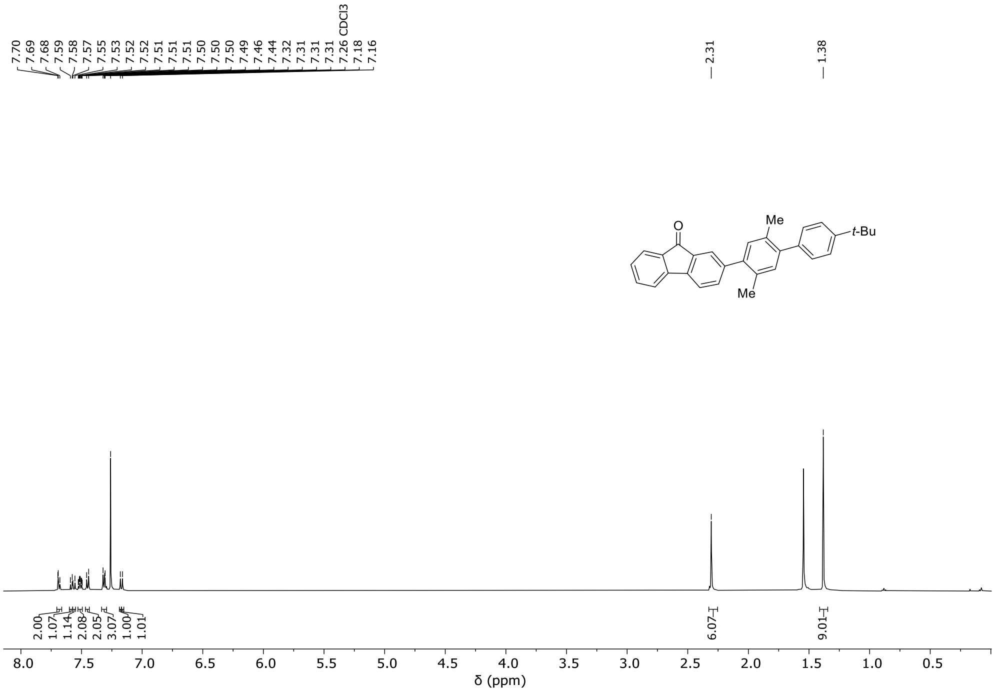

Figure S25. ^{1}H NMR (500 MHz) spectrum of compound **11** in $CDCl_3$.

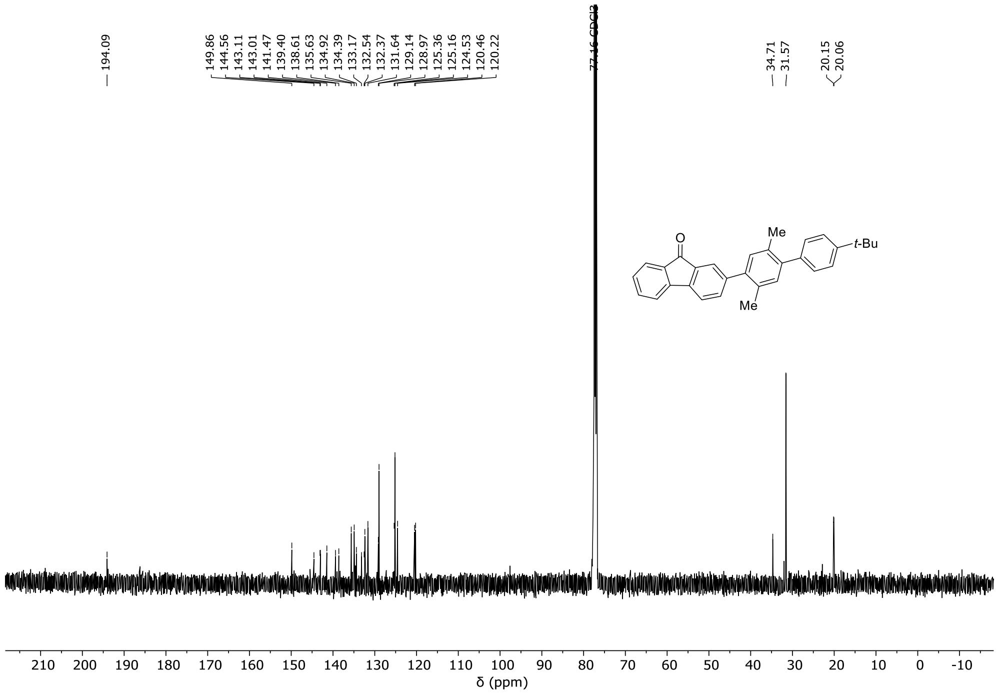

Figure S26. ^{13}C NMR (126 MHz) spectrum of compound **11** in $CDCl_3$.

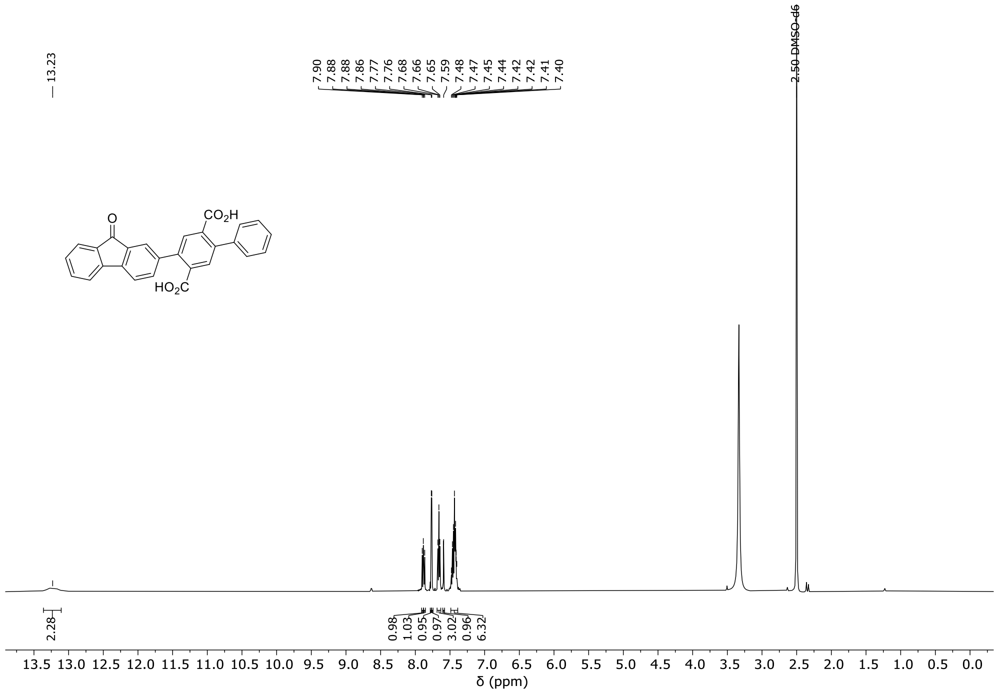

Figure S27. ^{1}H NMR (500 MHz) spectrum of compound **12** in DMSO-d_6.

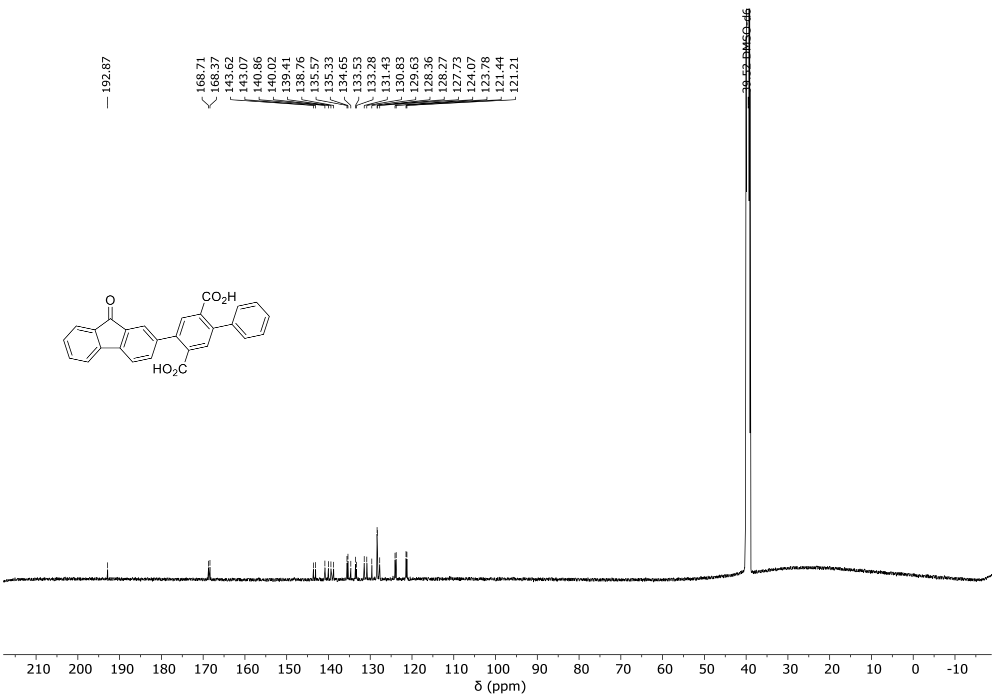

Figure S28. ^{13}C NMR (126 MHz) spectrum of compound **12** in DMSO-d_6.

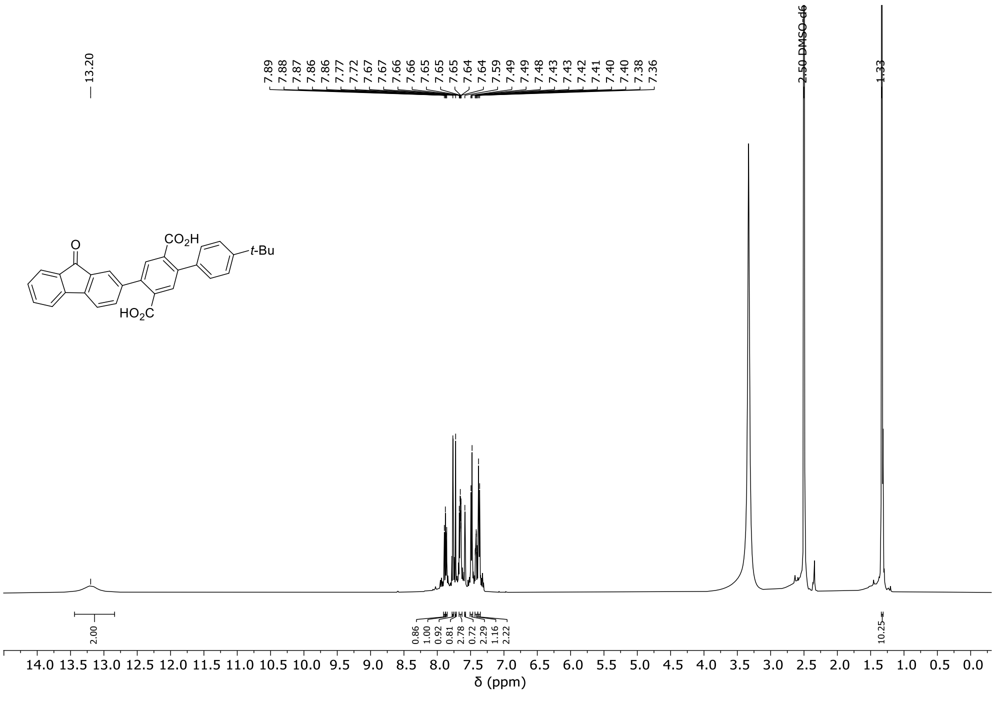

Figure S29. ^{1}H NMR (500 MHz) spectrum of compound **13** in DMSO-d_6.

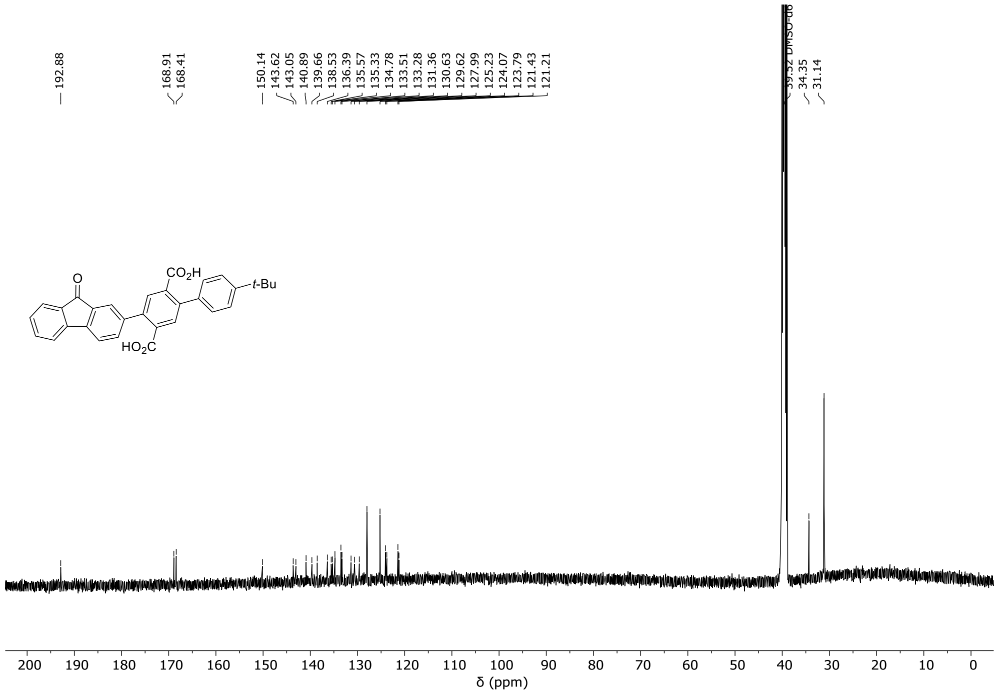

Figure S30. ^{13}C NMR (126 MHz) spectrum of compound **13** in DMSO-d_6.

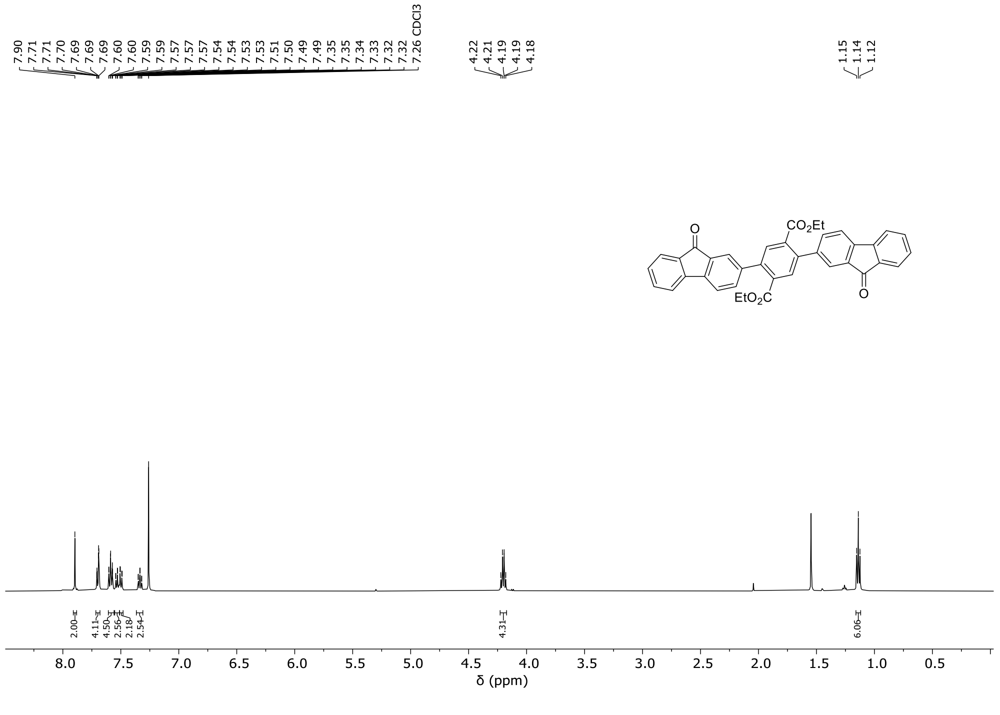

Figure S31. ^{1}H NMR (500 MHz) spectrum of compound **16** in $CDCl_3$.

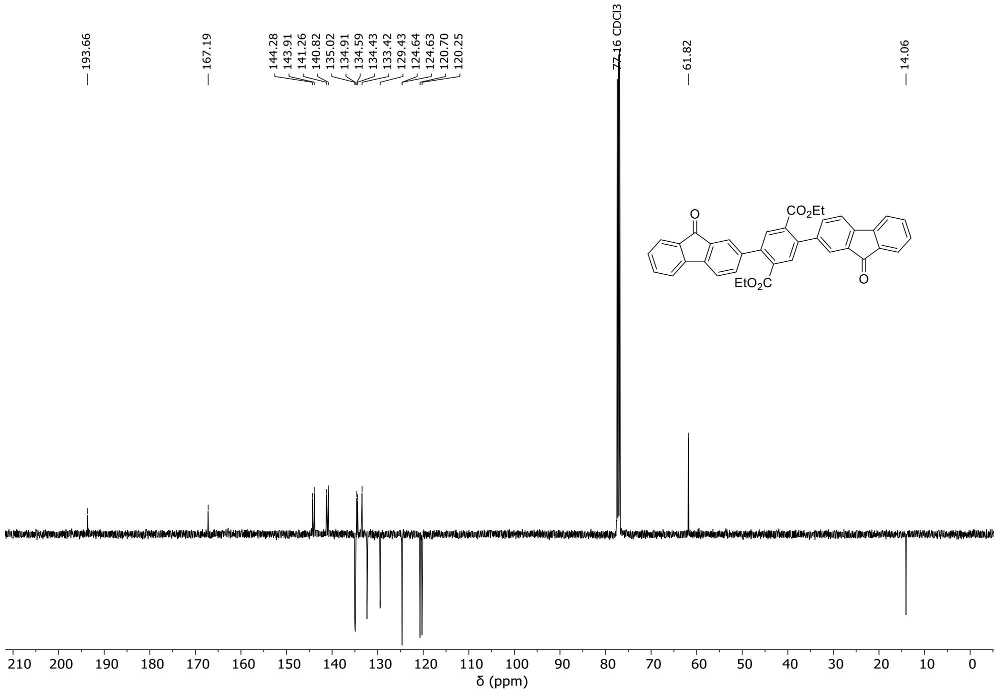

Figure S32. ^{13}C NMR (126 MHz) spectrum of compound **16** in $CDCl_3$.

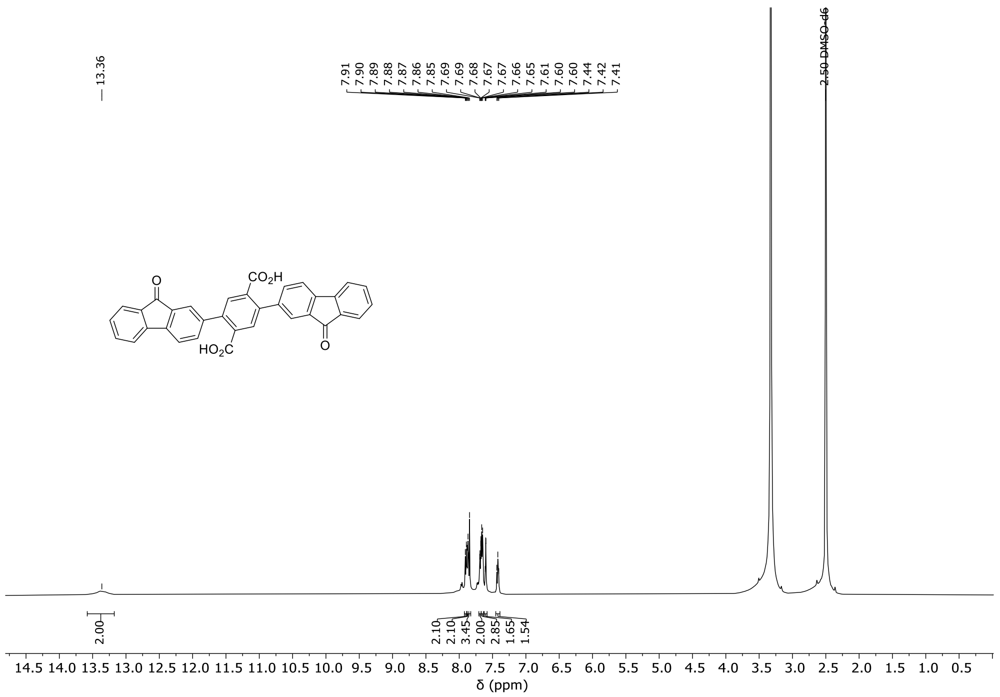

Figure S33. ^{1}H NMR (500 MHz) spectrum of compound **17** in DMSO-d_6.

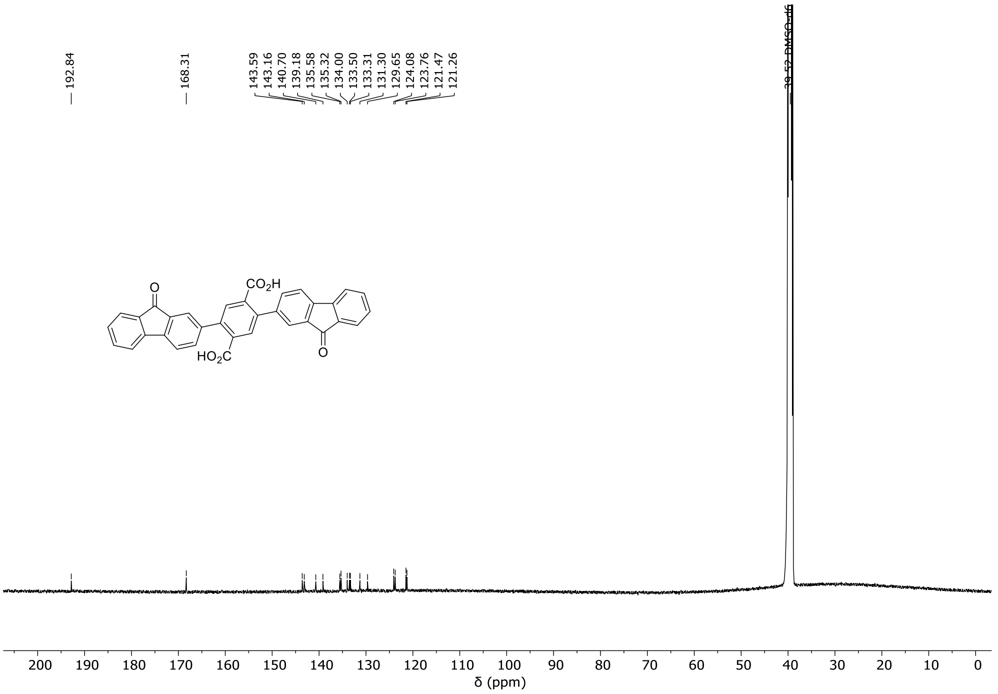

Figure S34. ^{13}C NMR (126 MHz) spectrum of compound **17** in DMSO-d_6.

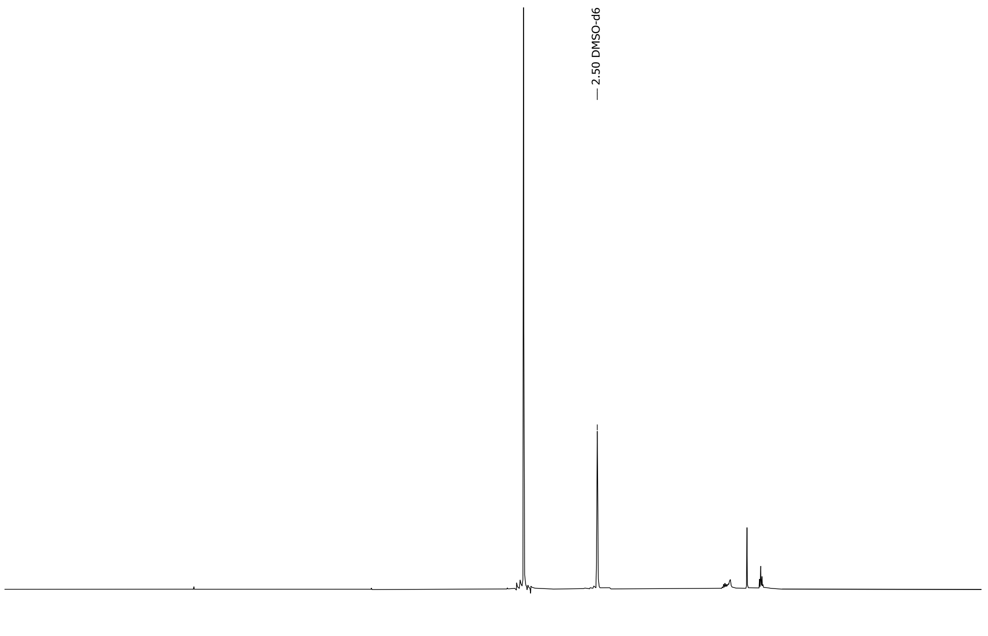

Figure S35. ^{1}H NMR (500 MHz) of neat CS_2 (DMSO-d_6 lock tube).

NMR Dilution Study of 1a

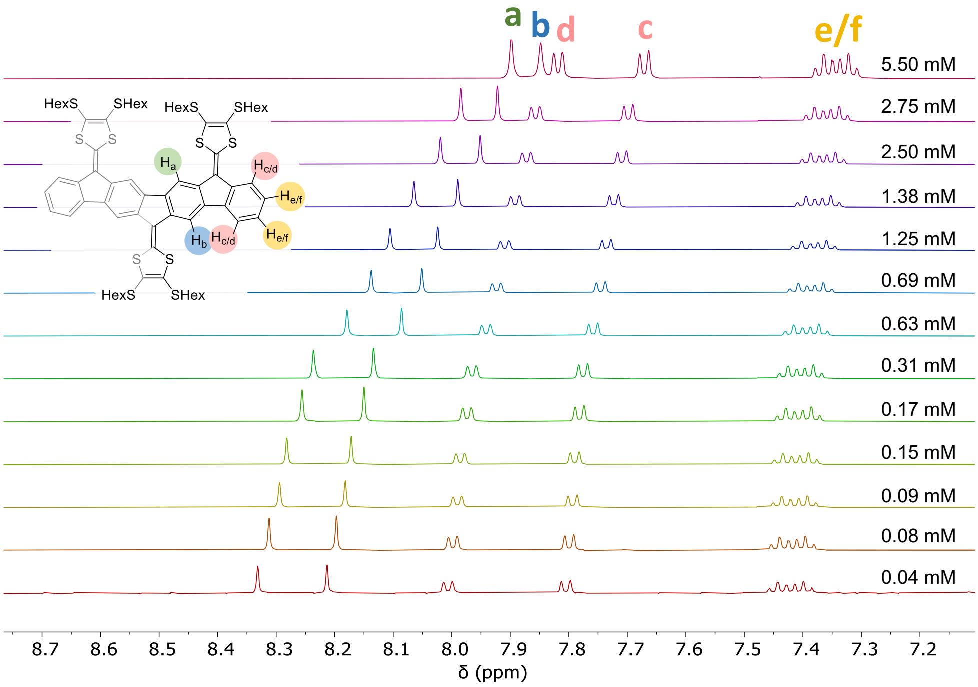

Figure S36. Segment of ^{1}H NMR spectroscopic data of tri-DTF **1a** recorded at different concentrations in CD_2Cl_2 at 25 °C.

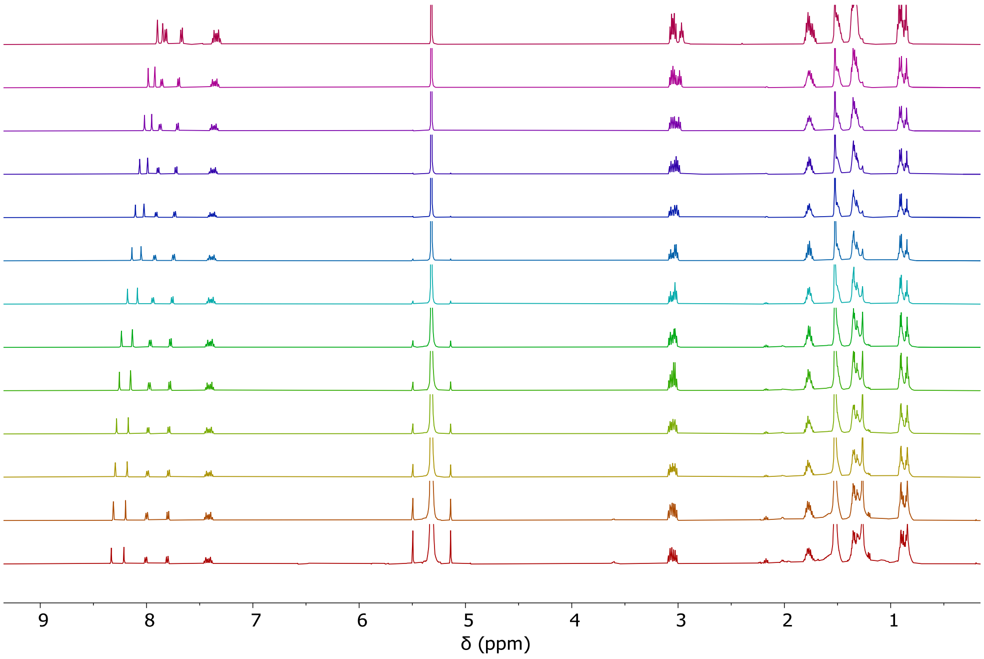

Figure S37. Full ^{1}H NMR spectra of tri-DTF **1a** recorded at different concentrations in CD_2Cl_2 at 25 °C.

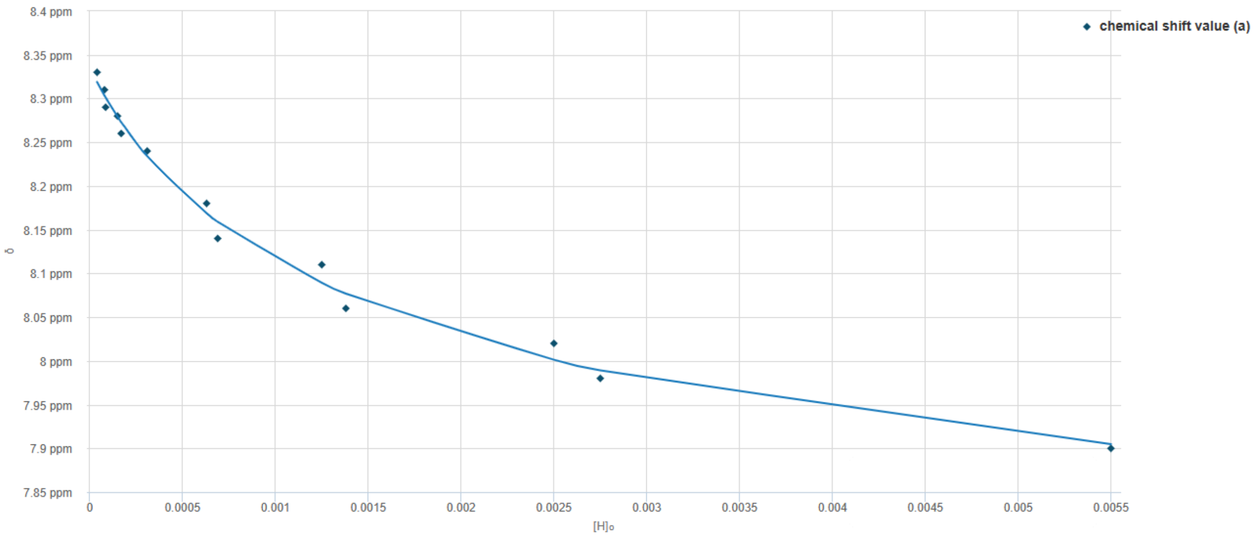

Figure S38. Chemical shift (δ_H) of the proton denoted H_a of compound **1a** as a function of the concentration of **1a**.

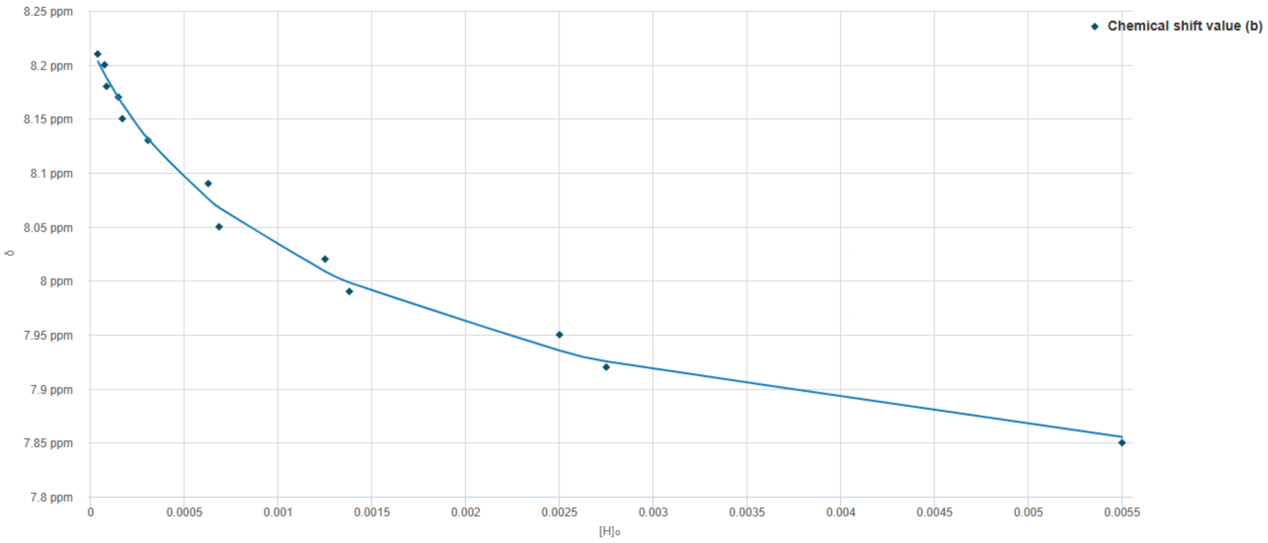

Figure S39. Chemical shift (δ_H) of the proton denoted H_B of compound **1a** as a function of the concentration of **1a**.

Table S2. Self-association constant (K_e) obtained using either proton denoted H_A or H_B for compound **1a**. Calculated by curve fitting of the change in chemical shift value as a function of the concentration using the online tool *Bindfit*[49] using a dimer aggregation model for simplicity. From these calculated values a self-association constant of ca. 600 M^{-1} is estimated.

	K_e	Error
H_A	589.78 M^{-1}	± 10.27%
H_B	610.96 M^{-1}	± 9.82%

HRMS Spectra

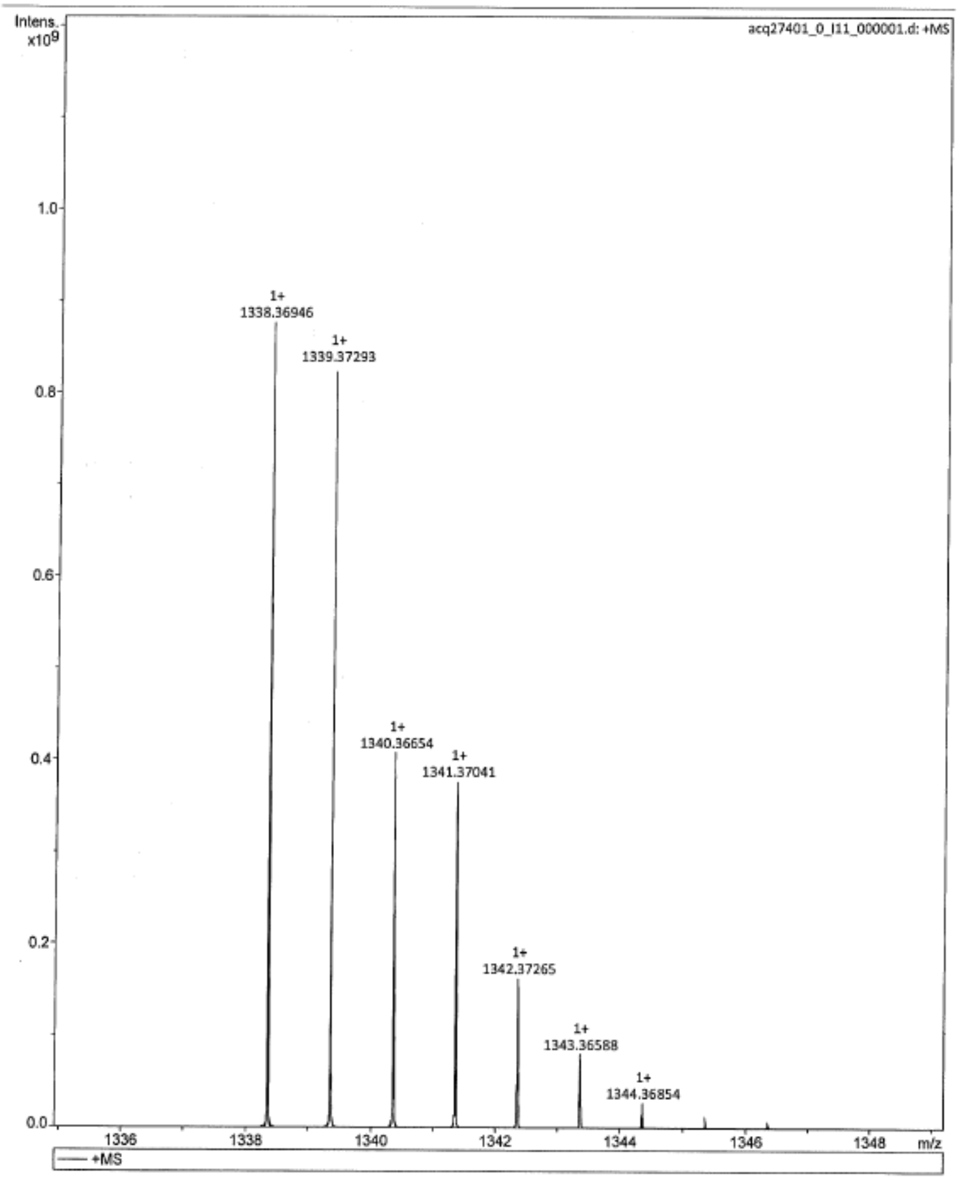

Figure S40. HRMS (MALDI$^+$ FT-ICR) spectrum of **1a**.

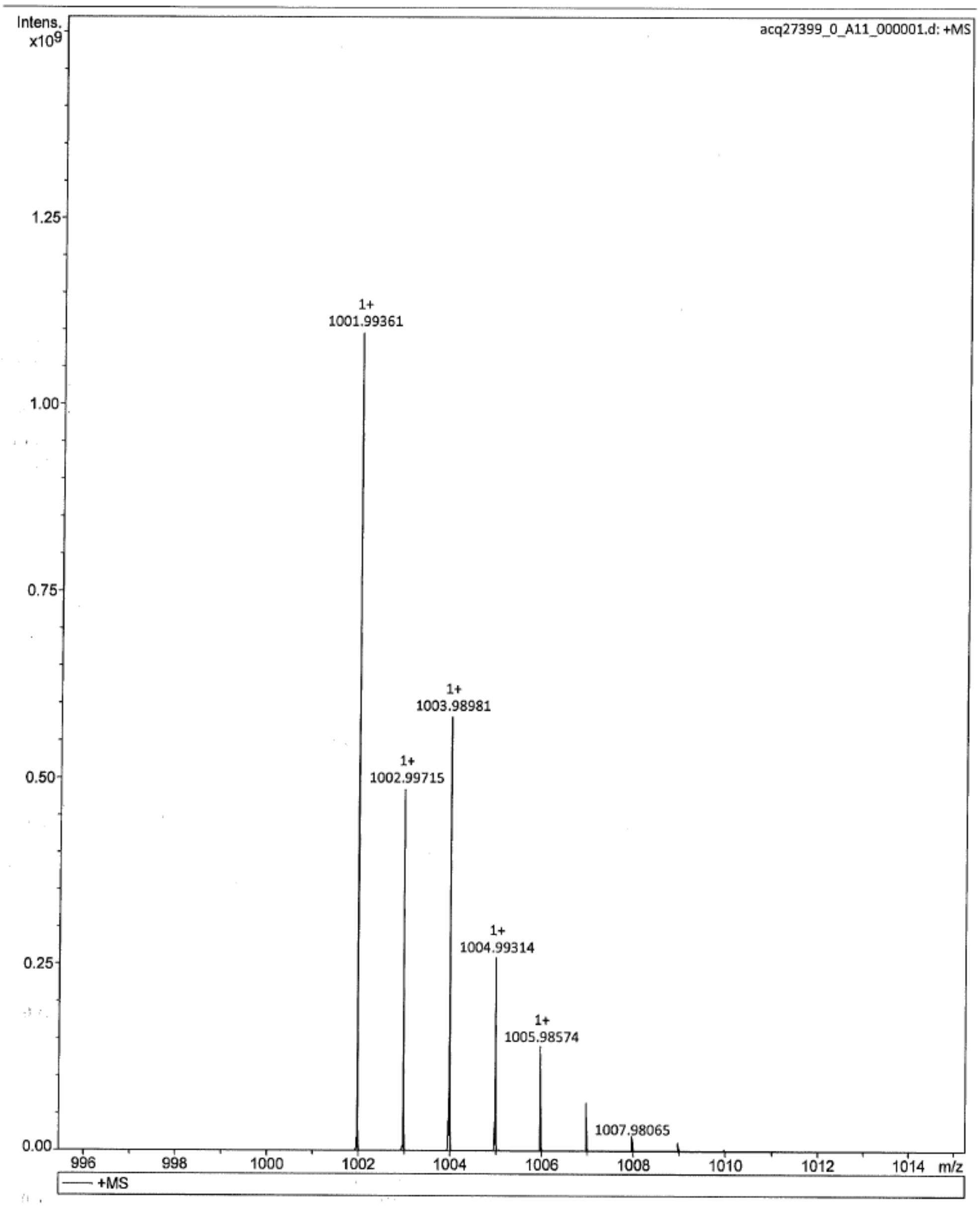

Figure S41. HRMS (MALDI^{+} FT-ICR) spectrum of **1b**.

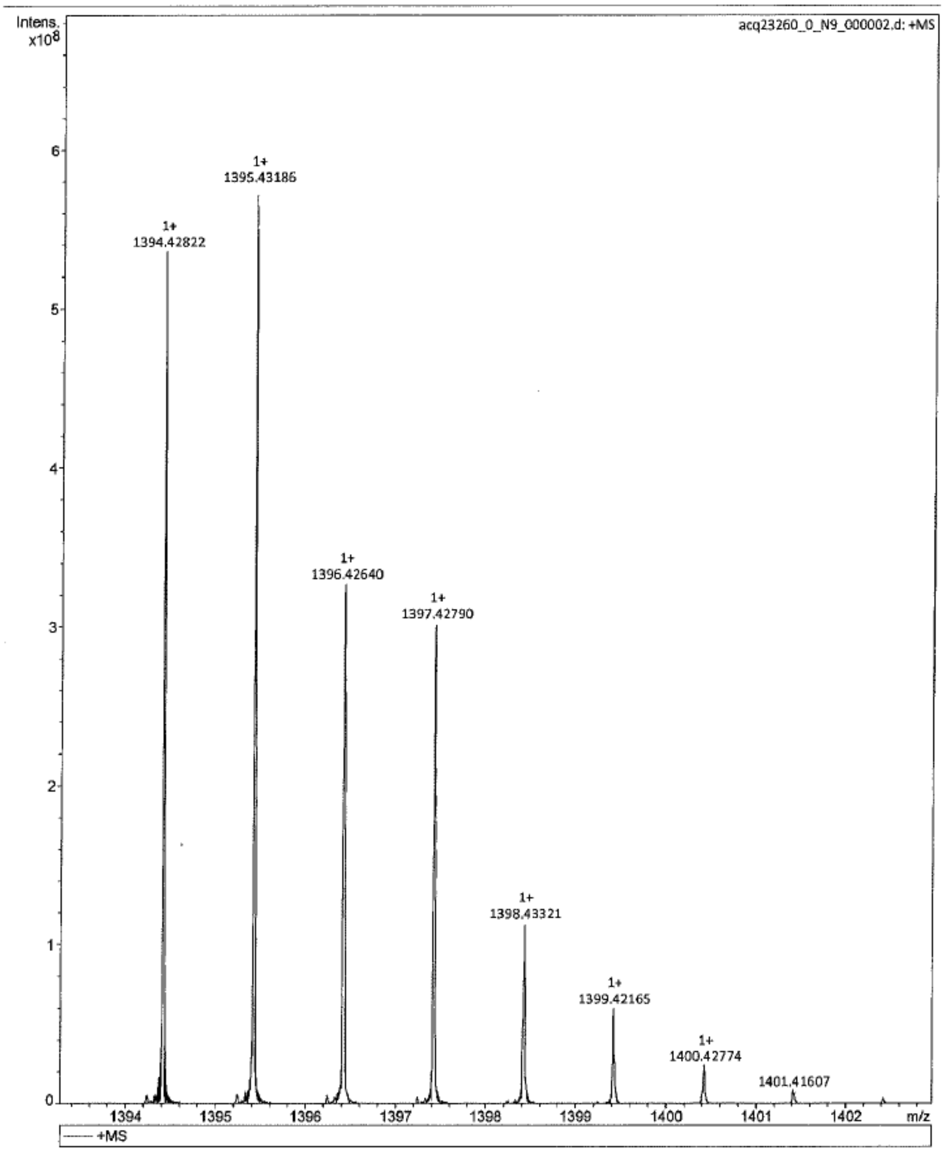

Figure S42. HRMS (MALDI$^+$ FT-ICR) spectrum of **2a**.

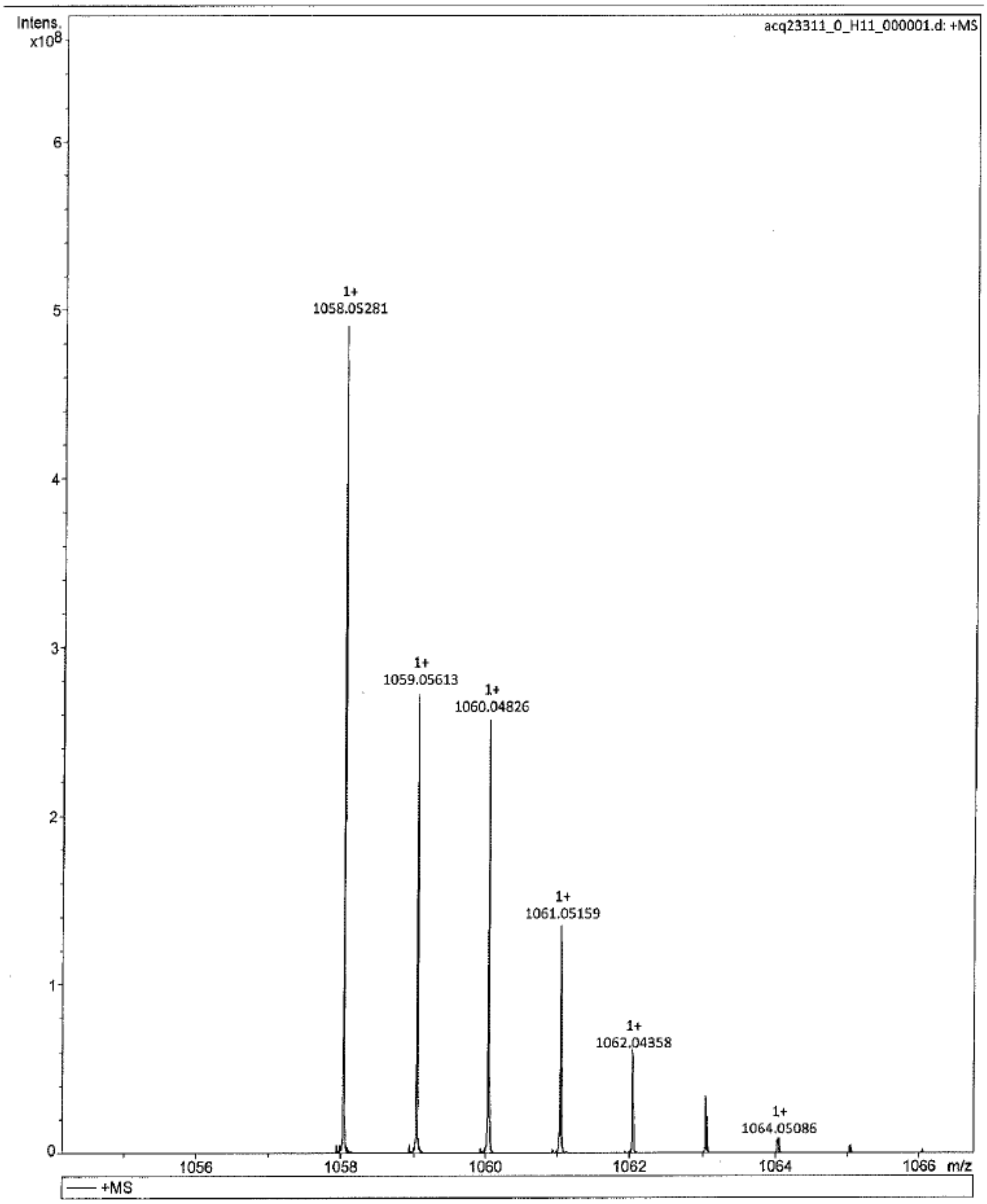

Figure S43. HRMS (MALDI$^+$ FT-ICR) spectrum of **2b**.

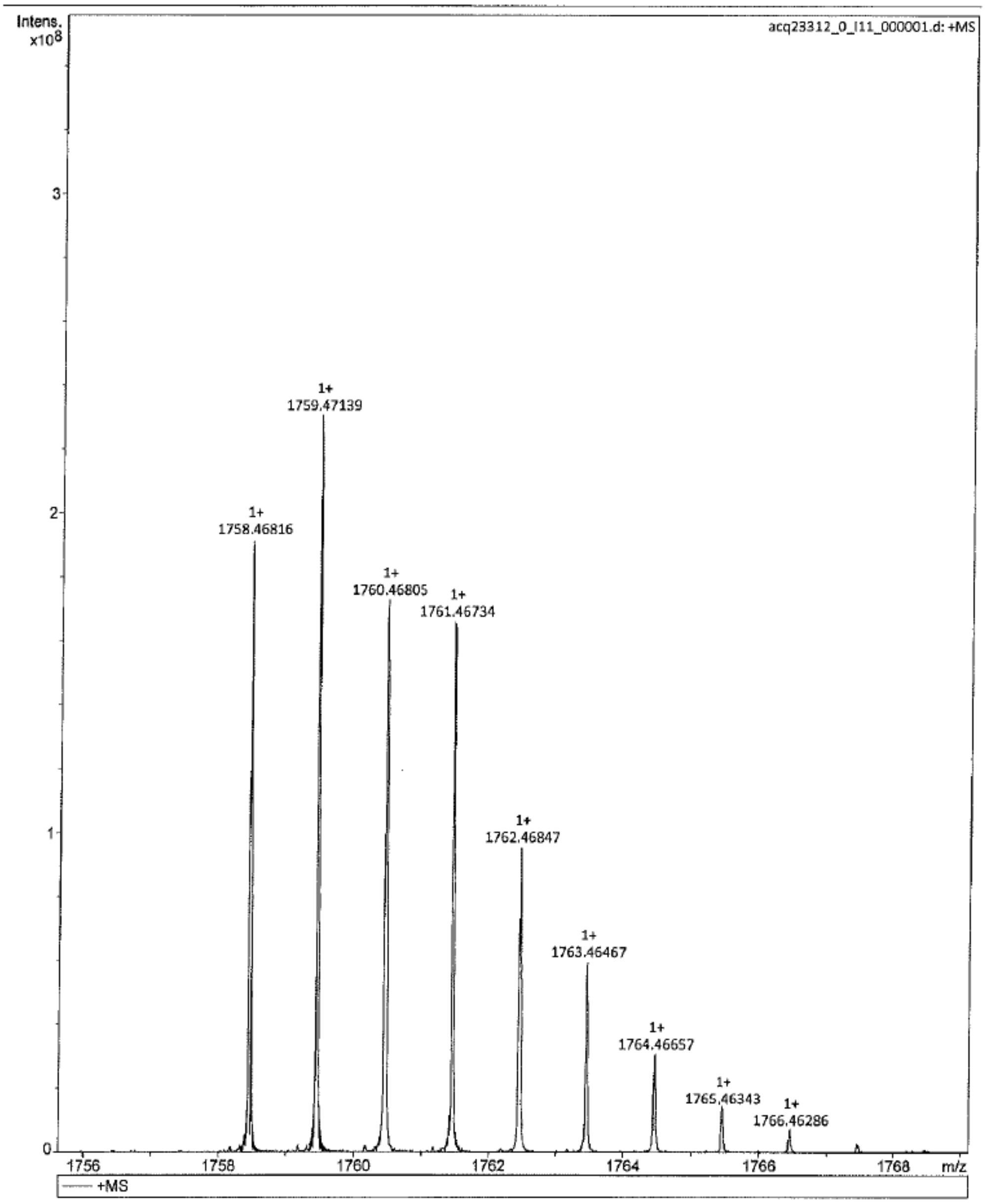

Figure S44. HRMS ($MALDI^+$ FT-ICR) spectrum of **3a**.

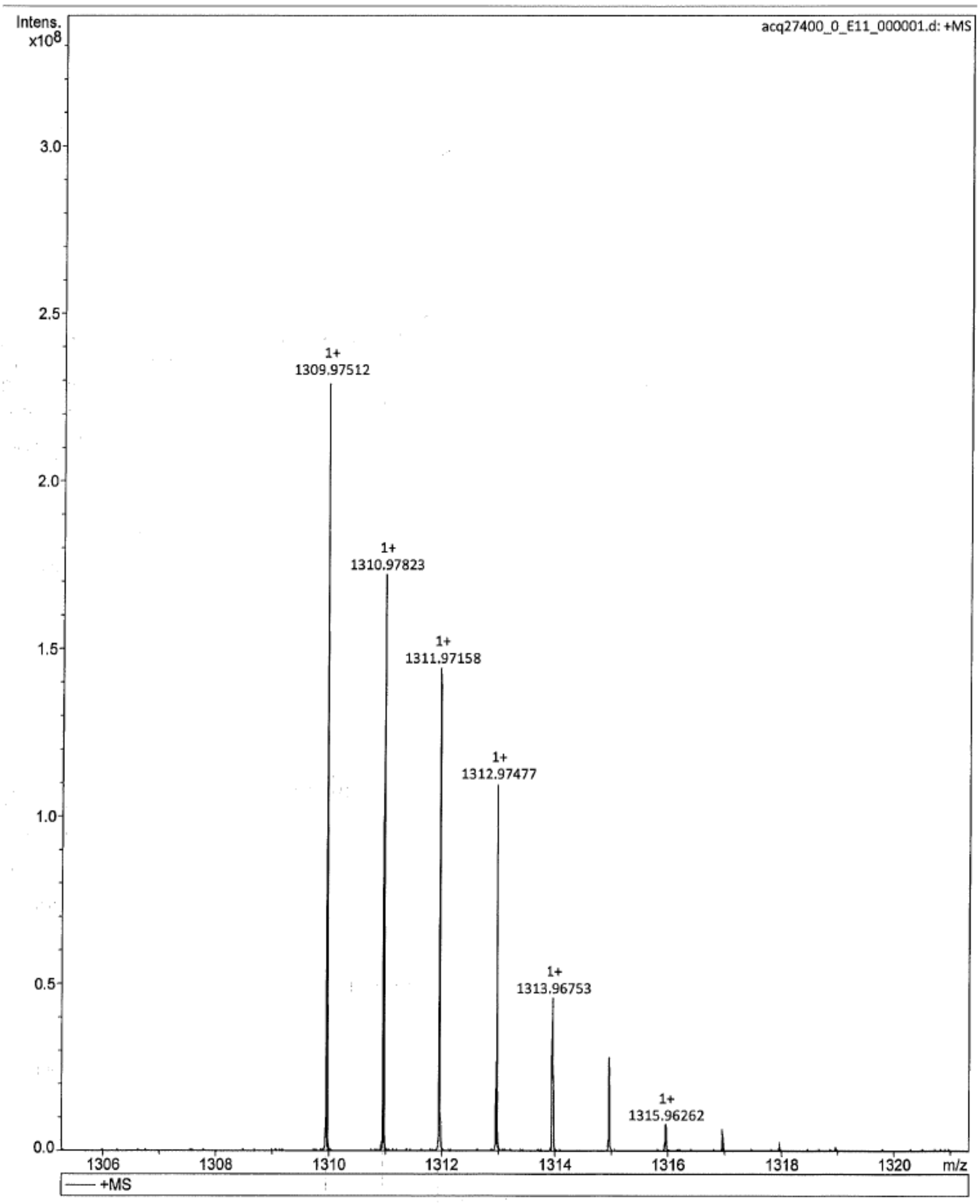

Figure S45. HRMS (MALDI$^+$ FT-ICR) spectrum of **3b**.

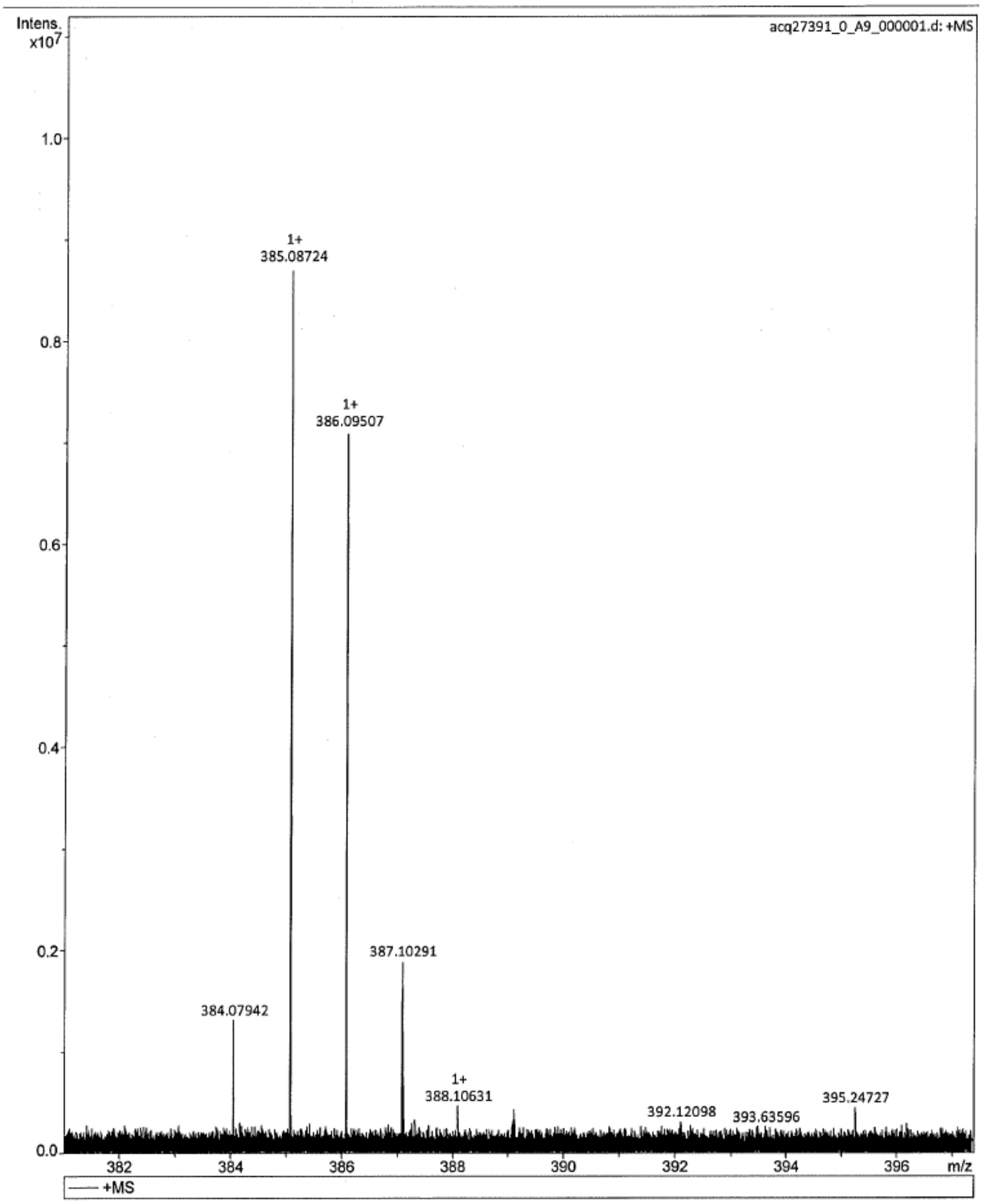

Figure S46. HRMS (MALDI$^+$ FT-ICR) spectrum of **4**.

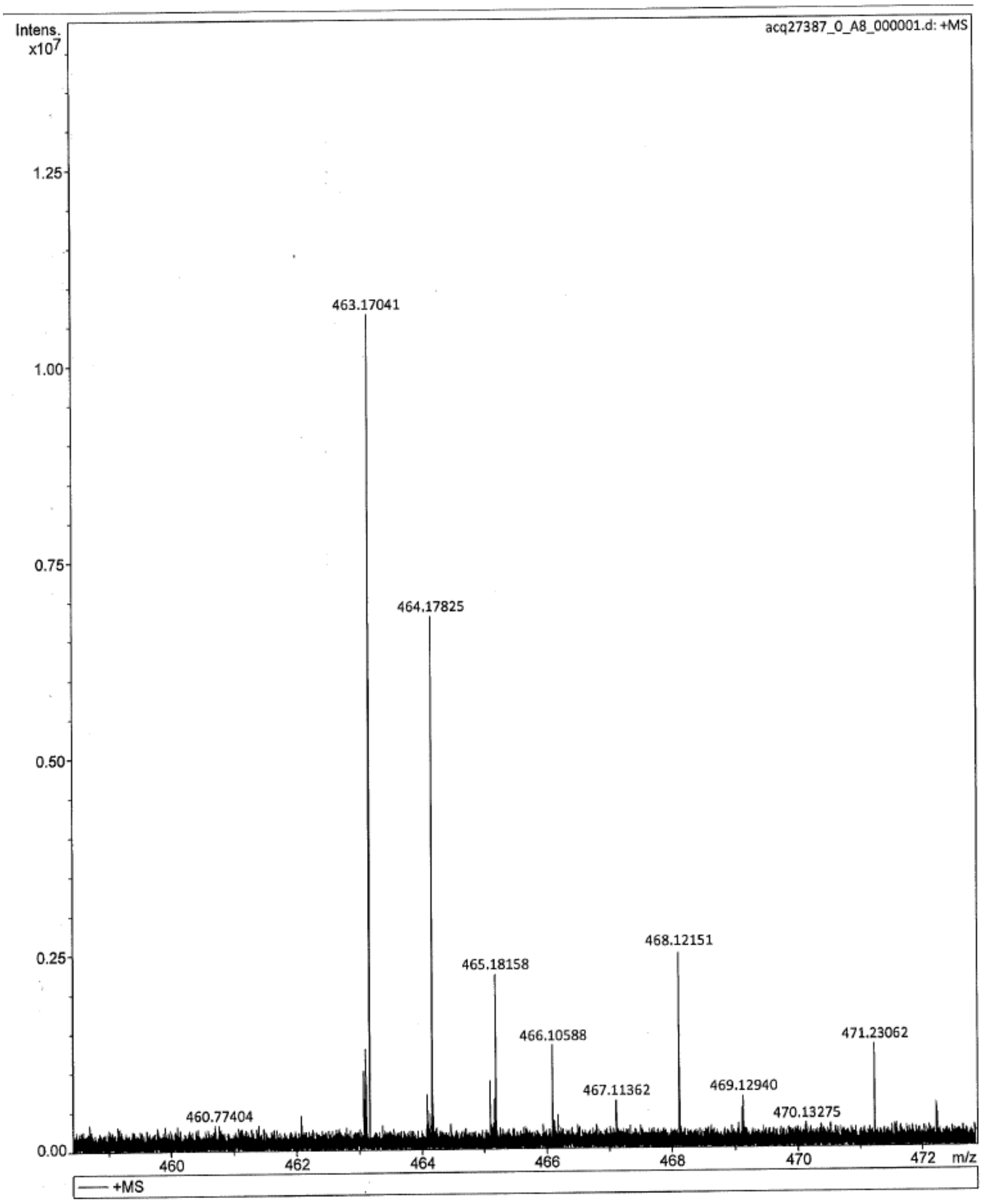

Figure S47. HRMS (MALDI$^+$ FT-ICR) spectrum of **5**.

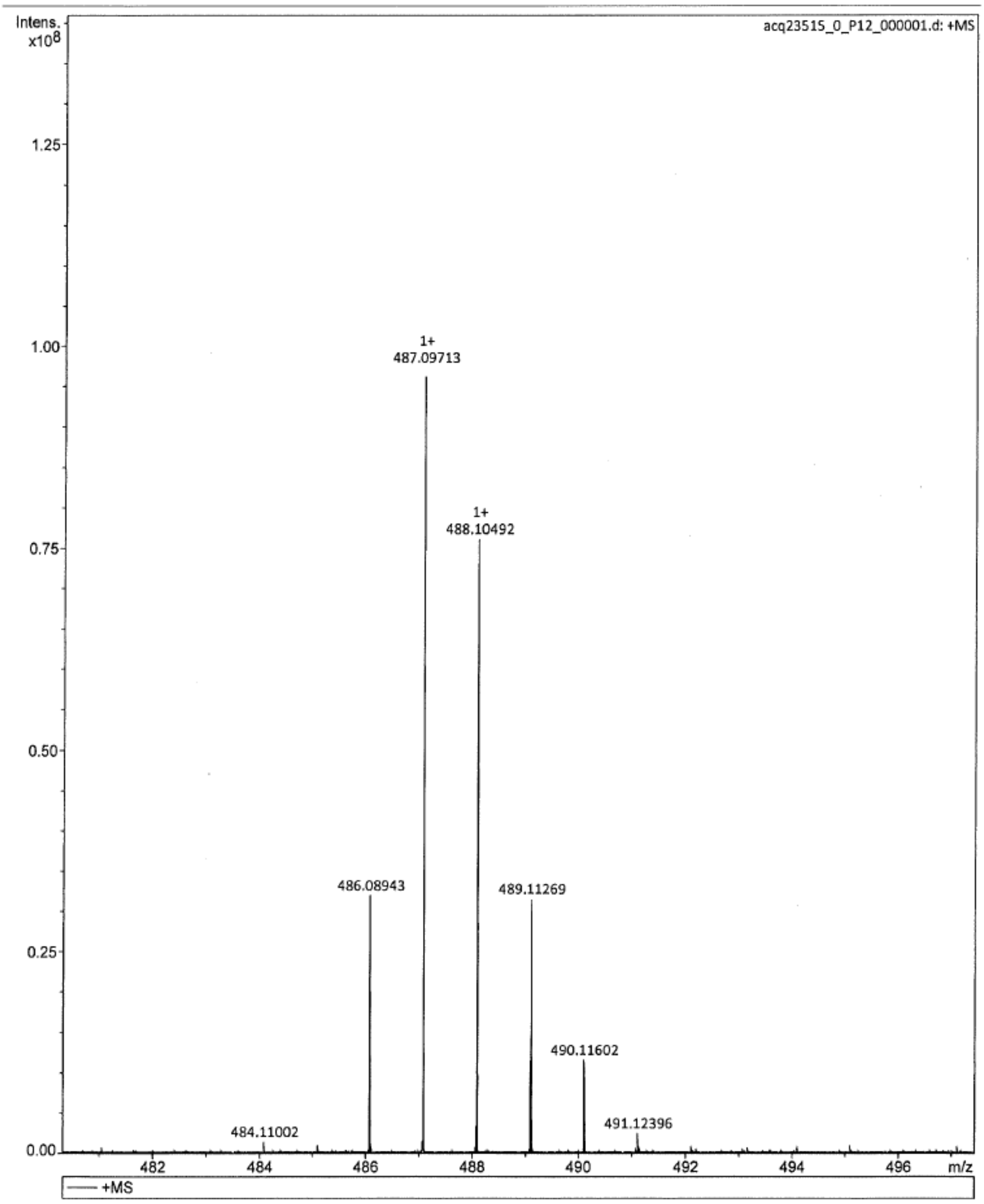

Figure S48. HRMS (MALDI$^+$ FT-ICR) spectrum of **6**.

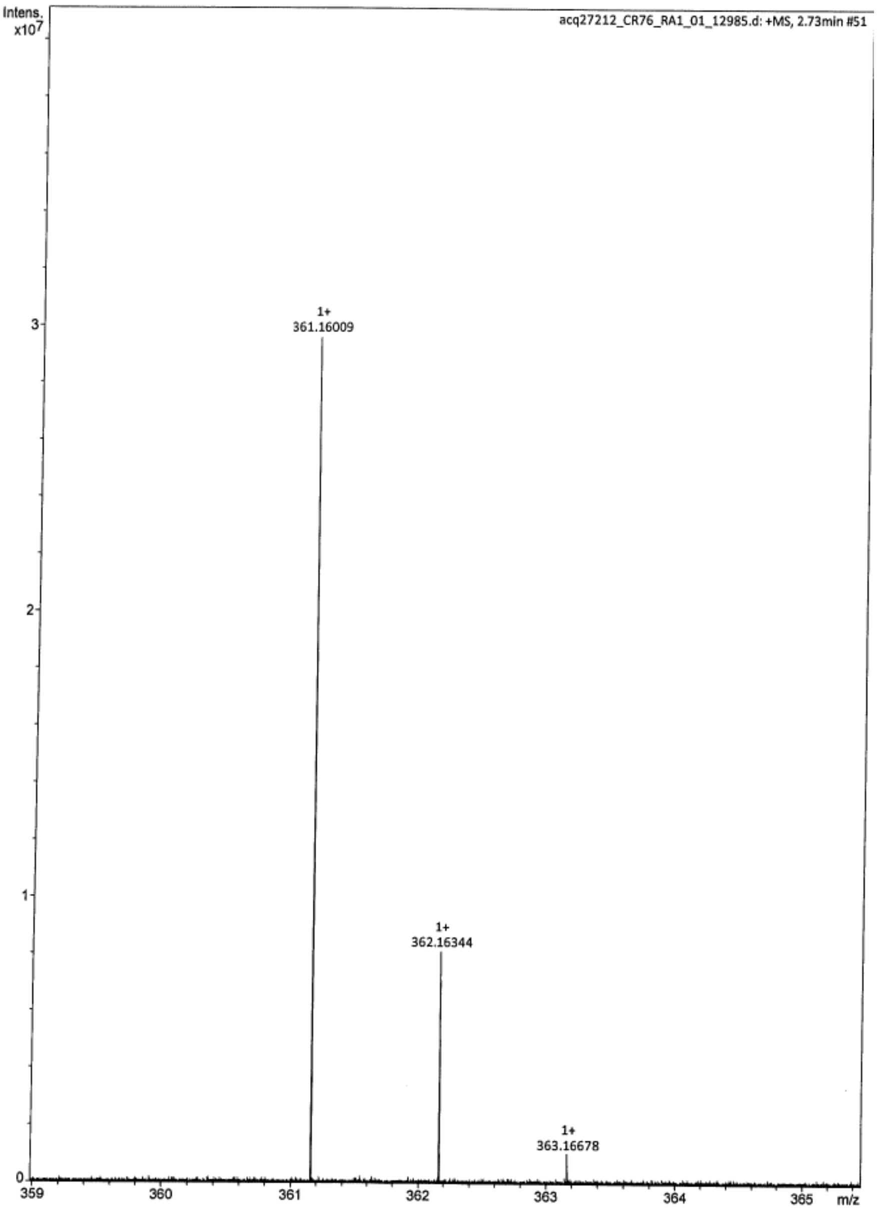

Figure S49. HRMS (ESP$^+$ FT-ICR) spectrum of **10**.

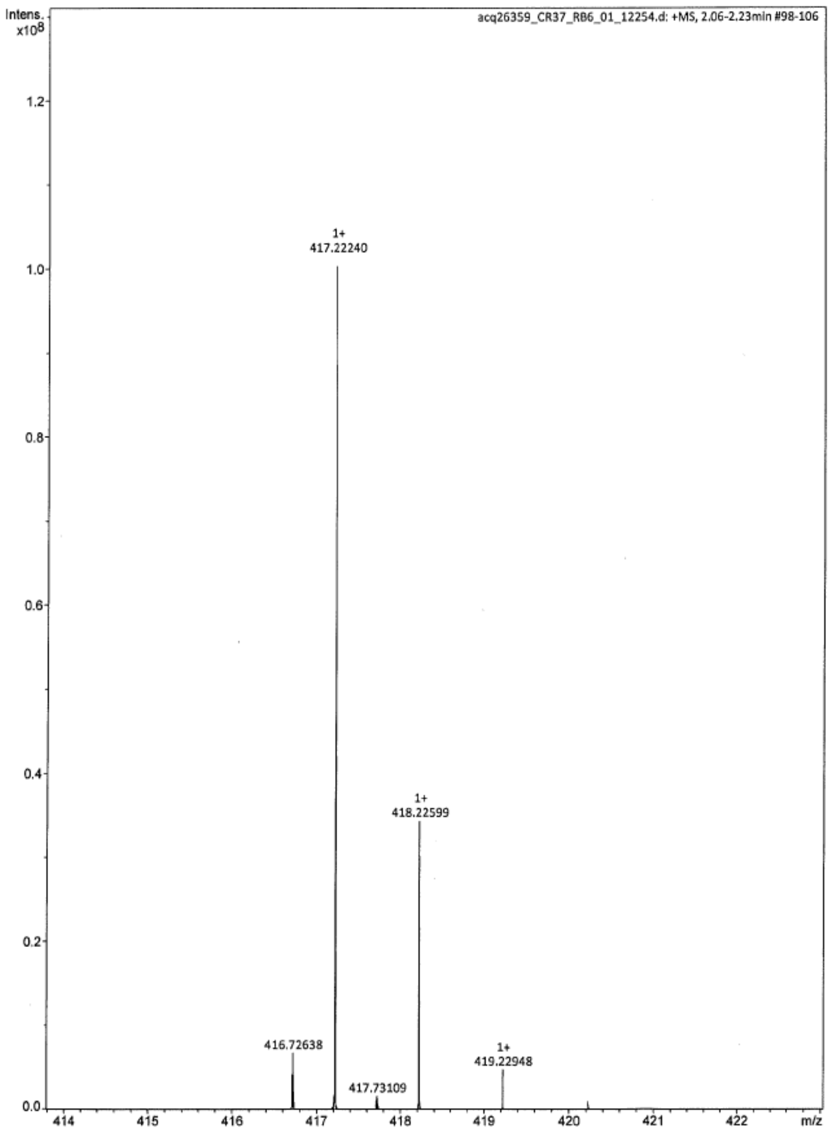

Figure S50. HRMS (ESP$^+$ FT-ICR) spectrum of **11**.

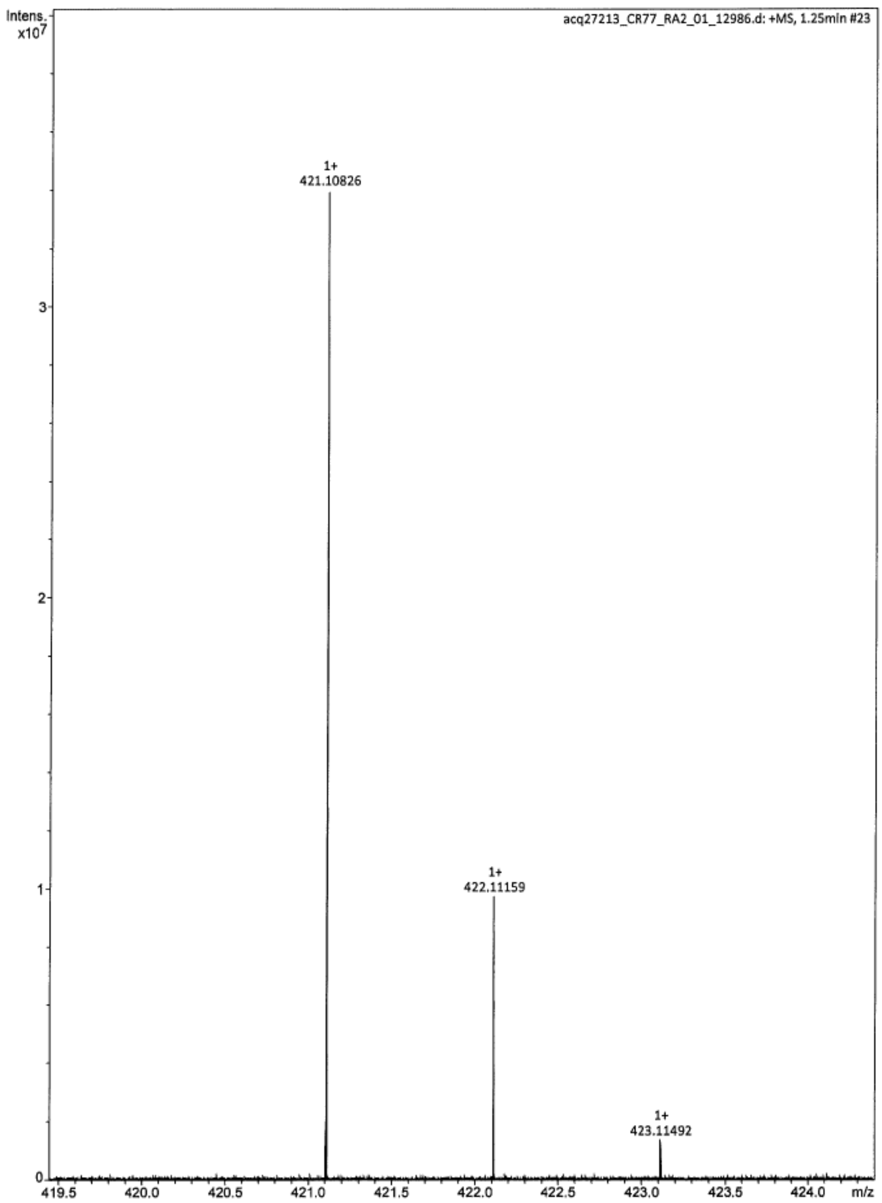

Figure S51. HRMS (ESP$^+$ FT-ICR) spectrum of **12**.

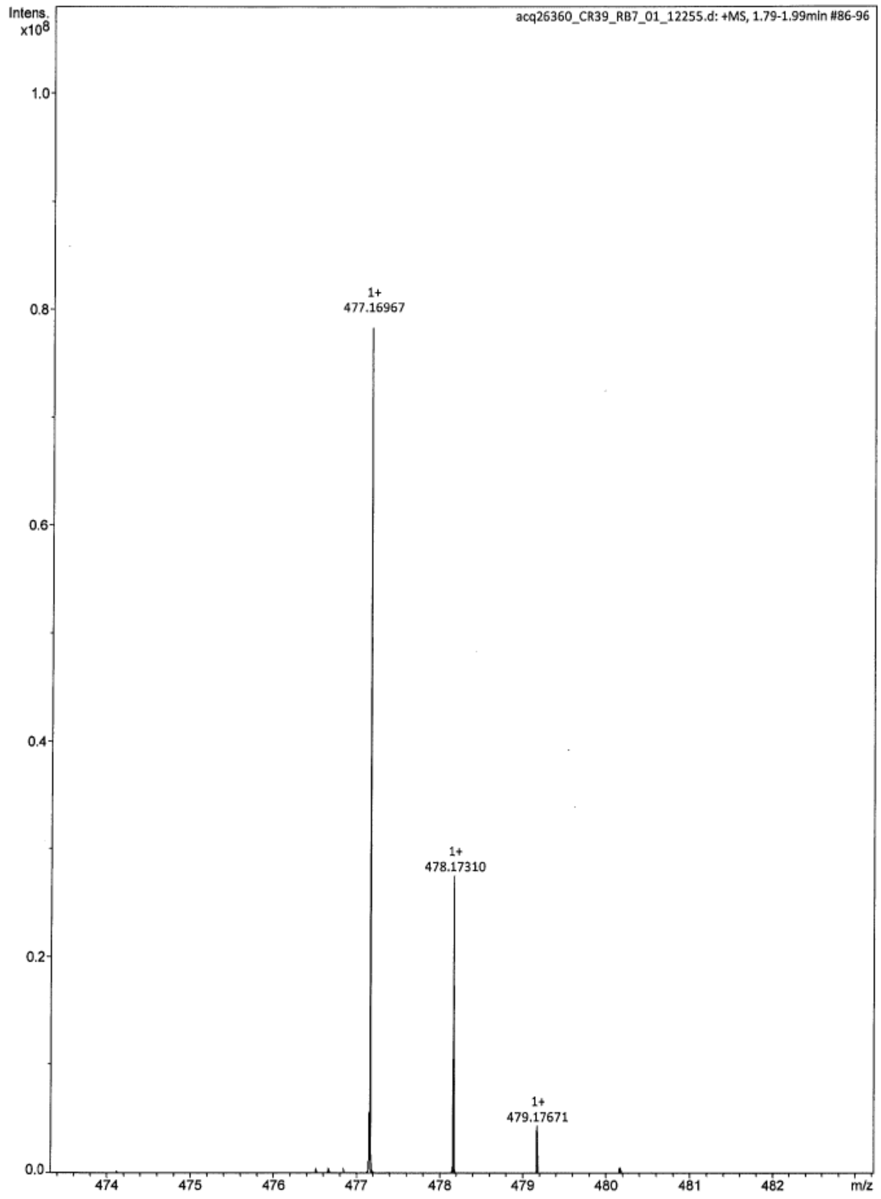

Figure S52. HRMS (ESP$^+$ FT-ICR) spectrum of **13**.

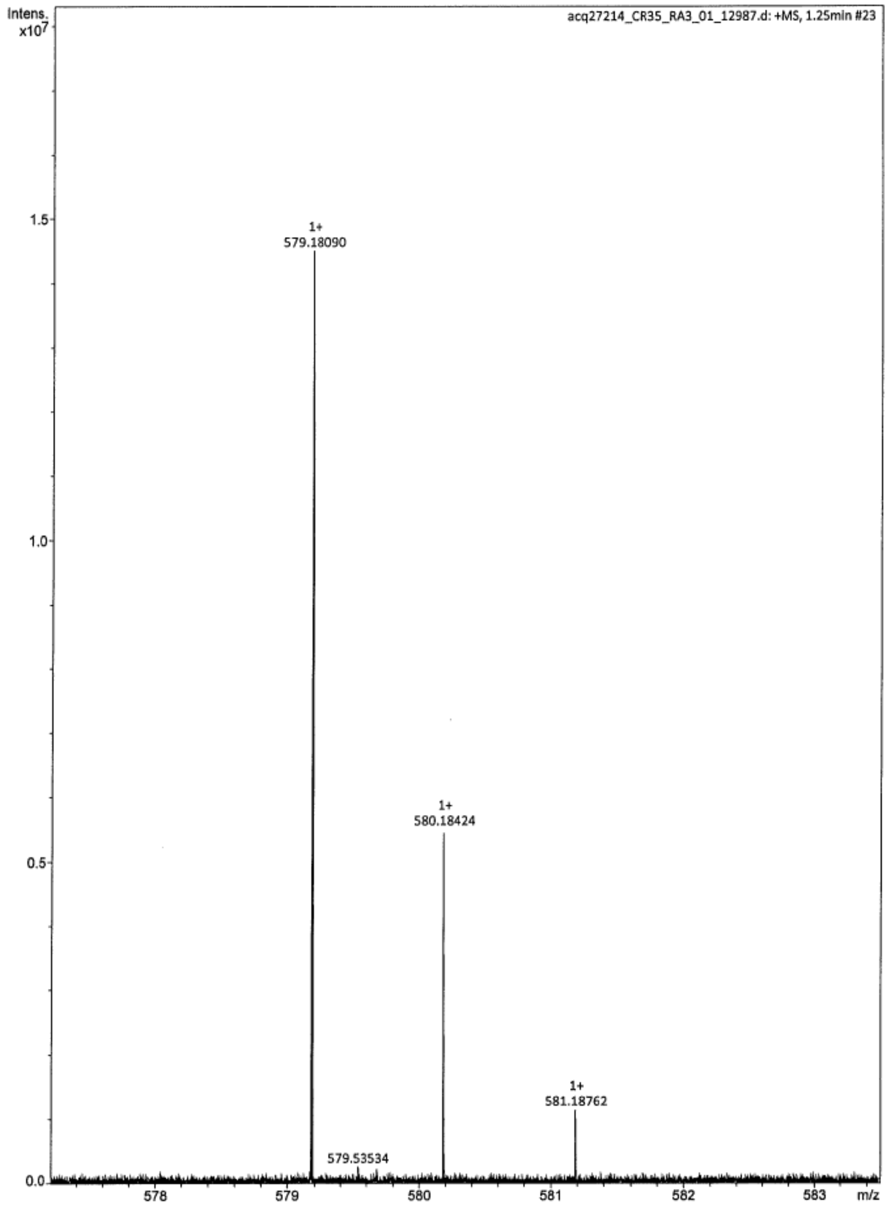

Figure S53. HRMS (ESP$^+$ FT-ICR) spectrum of **16**.

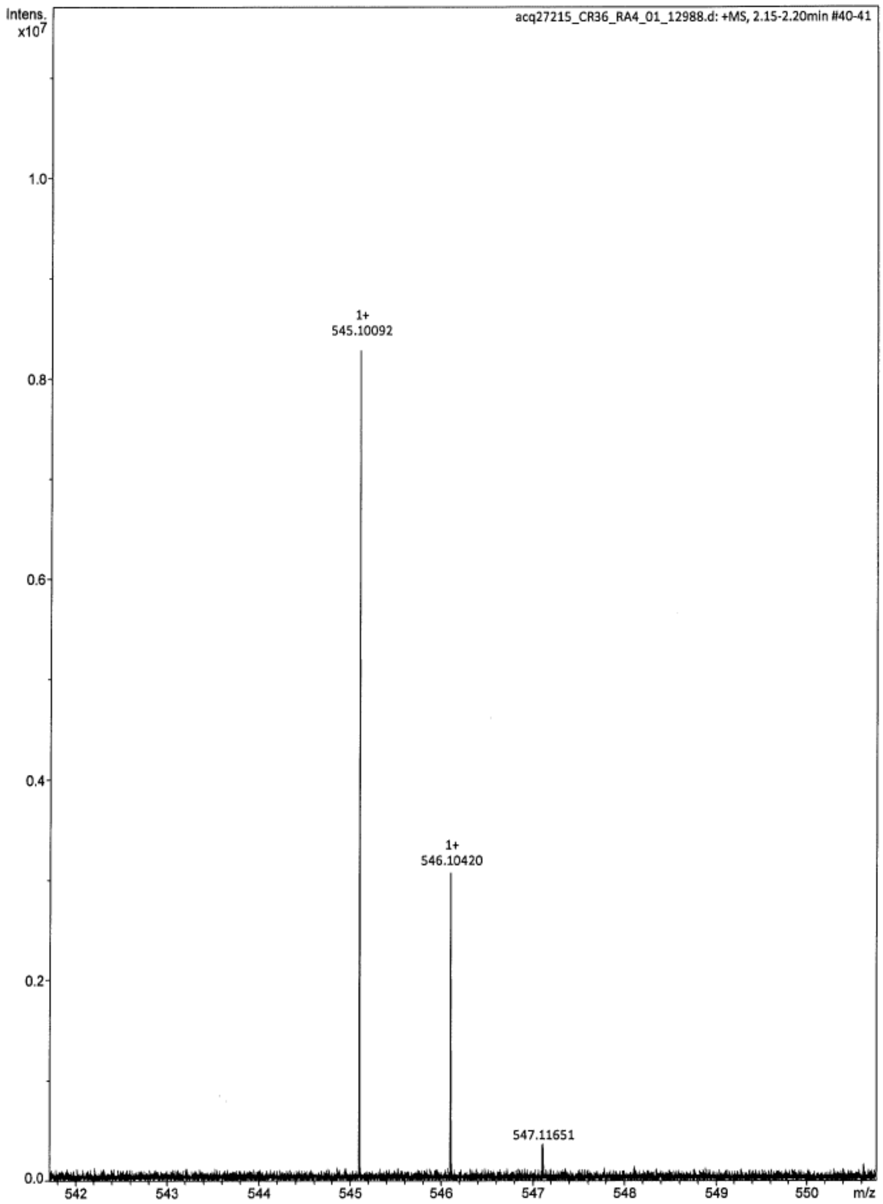

Figure S54. HRMS (ESP$^+$ FT-ICR) spectrum of **17**.

UV-Vis Absorption Spectra

Table S3. Absorption maxima and extinction coefficients for compounds **1-3** in CH_2Cl_2 at room temperature.

Compound	λ_{max} (nm), ε (in brackets; 10^4 cm^{-1} M^{-1})
**1a**	496 (9.60), 469 (5.97), 379 (5.01), 360[a] (4.31), 322 (5.84), 291[a] (4.84), 273 (6.64)
**1b**	495 (12.9), 467 (7.97), 379 (6.85), 360[a] (5.86), 320 (7.88), 291[a] (6.43), 273 (8.66)
**2a**	496 (9.96), 469 (6.16), 391 (5.60), 363[a] (4.87), 322 (6.20), 291[a] (5.20), 272 (6.86)
**2b**	493 (9.12), 466 (5.63), 381 (5.22), 362[a] (4.42), 320 (5.85), 289[a] (4.80), 272 (6.30)
**3a**	507 (10.7), 478 (6.87), 428 (7.24), 399 (8.69), 380[a] (6.62), 332 (6.94), 296 (5.94), 273 (7.27)
**3b**	505 (6.14), 477 (4.56), 426 (5.21), 399 (6.15), 380[a] (4.90), 331 (5.02), 295 (4.53), 272 (5.43)

[a] Shoulder.

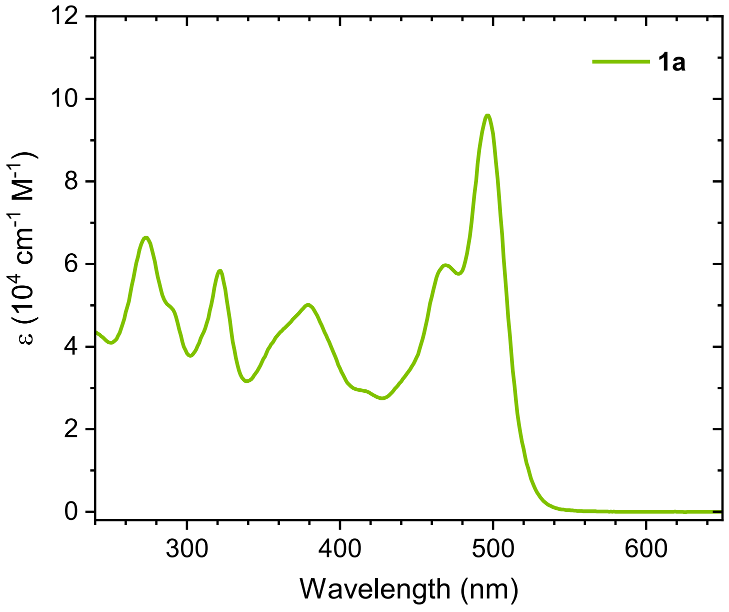

Figure S55. UV-Vis absorption spectrum of compound **1a** recorded in CH_2Cl_2.

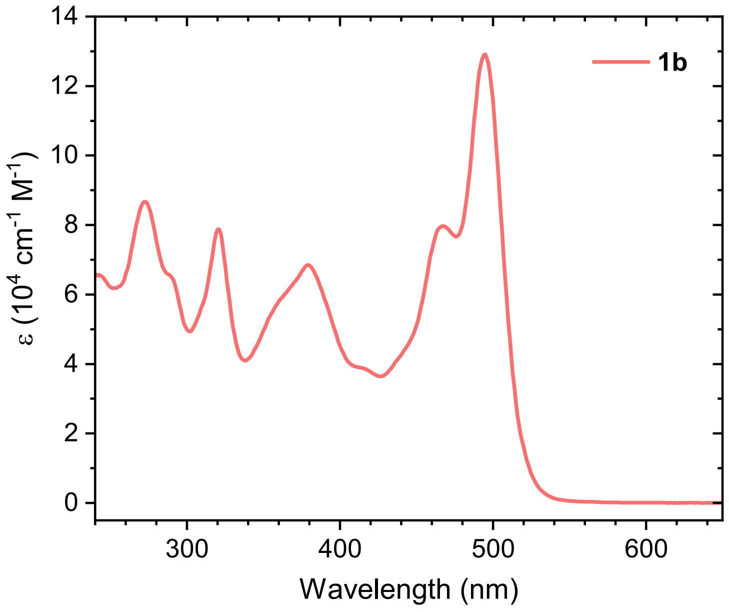

Figure S56. UV-Vis absorption spectrum of compound **1b** recorded in CH_2Cl_2.

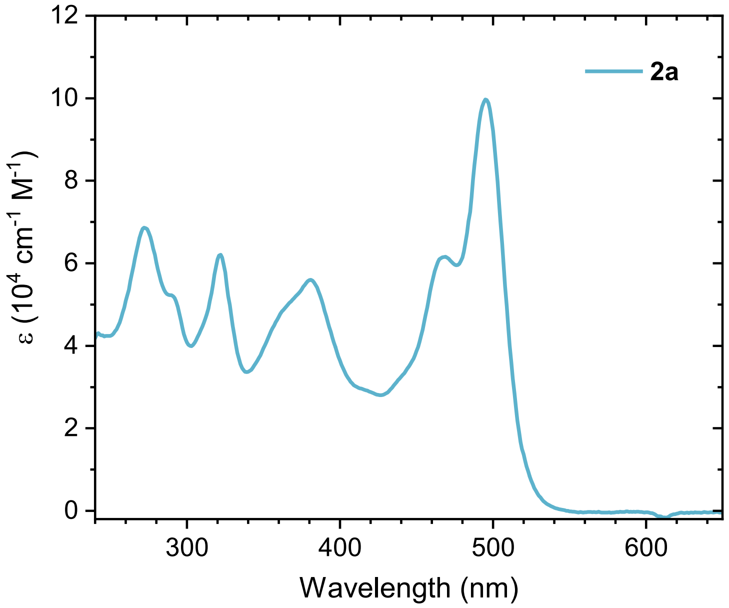

Figure S57. UV-Vis absorption spectrum of compound **2a** recorded in CH_2Cl_2.

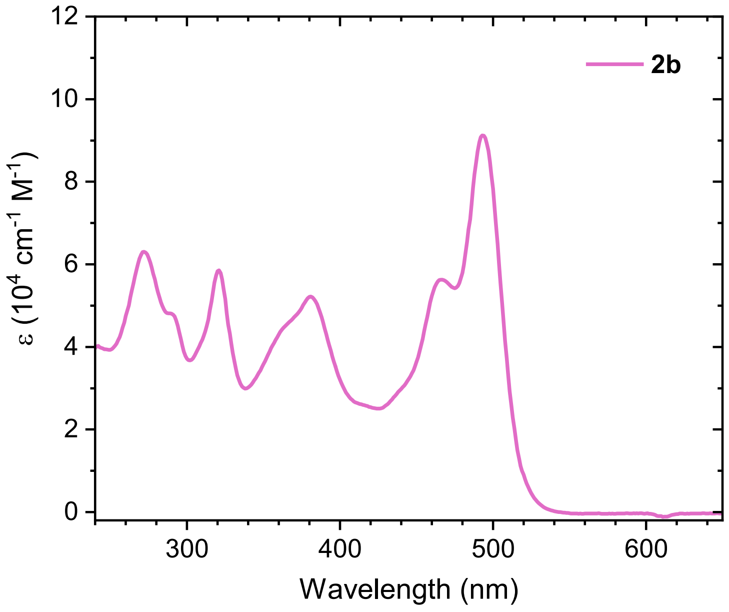

Figure S58. UV-Vis absorption spectrum of compound **2b** recorded in CH_2Cl_2.

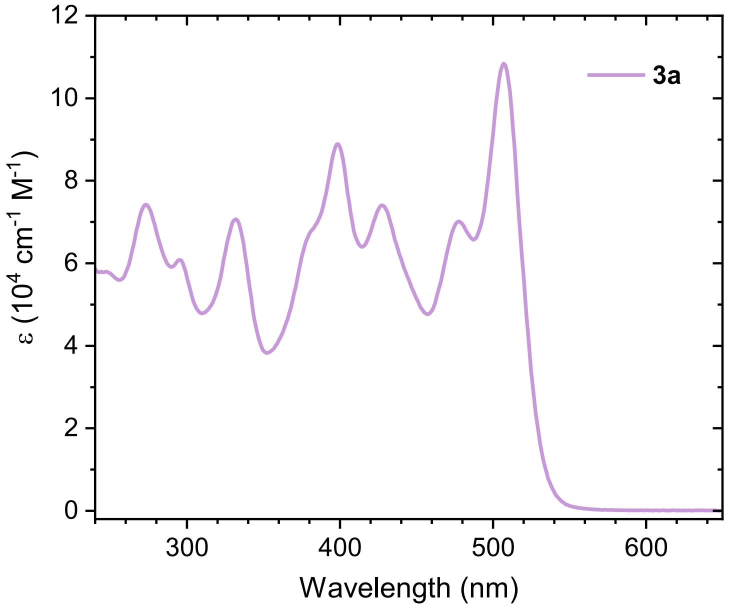

Figure S59. UV-Vis absorption spectrum of compound **3a** recorded in CH_2Cl_2.

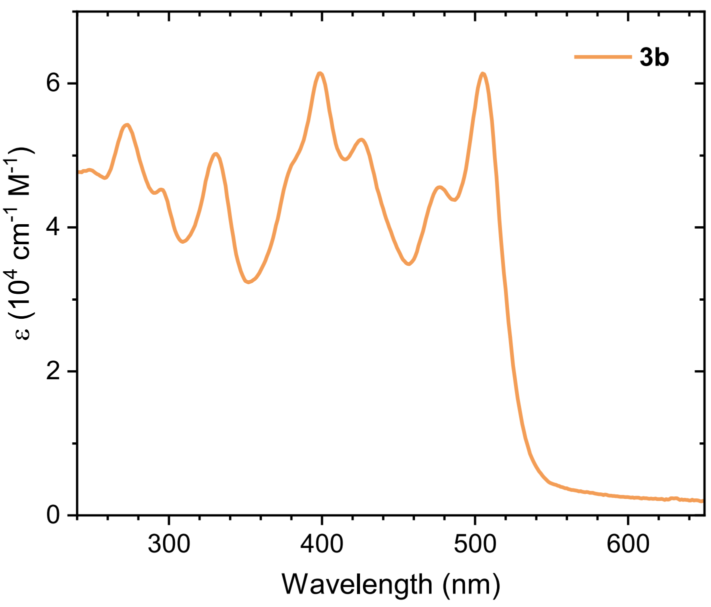

Figure S60. UV-Vis absorption spectrum of compound **3b** recorded in CH_2Cl_2.

Electrochemistry Data

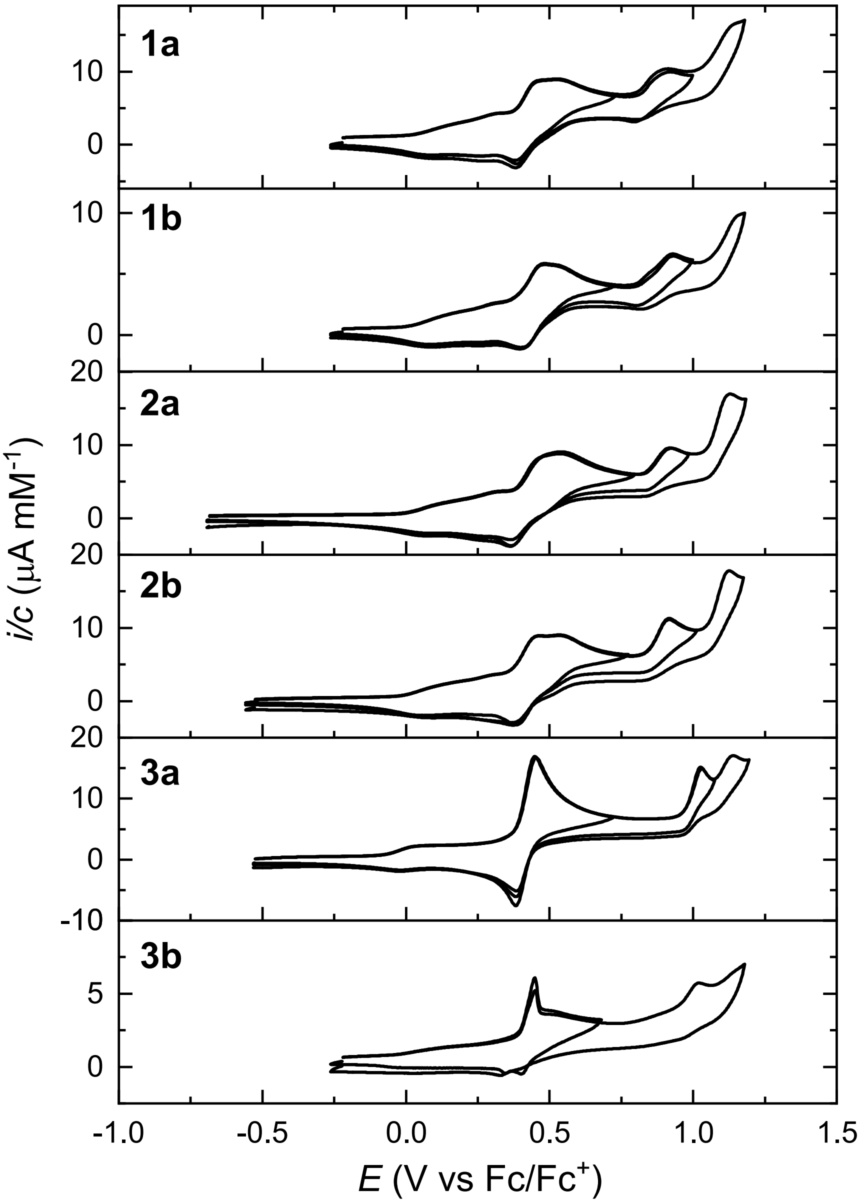

Figure S61. Cyclic voltammograms of compounds **1**-**3**. The voltammograms were recorded in CH_2Cl_2 using n-Bu_4NPF_6 (0.1M) as supporting electrolyte with a scan rate of 0.1 V/s. The compounds were measured at concentrations (from the top): 0.25 mM (**1a**), 0.25 mM (**1b**), 0.50 mM (**2a**), 0.50 mM (**2b**), 0.25 mM (**3a**). Due to solubility issues for **3b** it was not possible to determine the concentration.

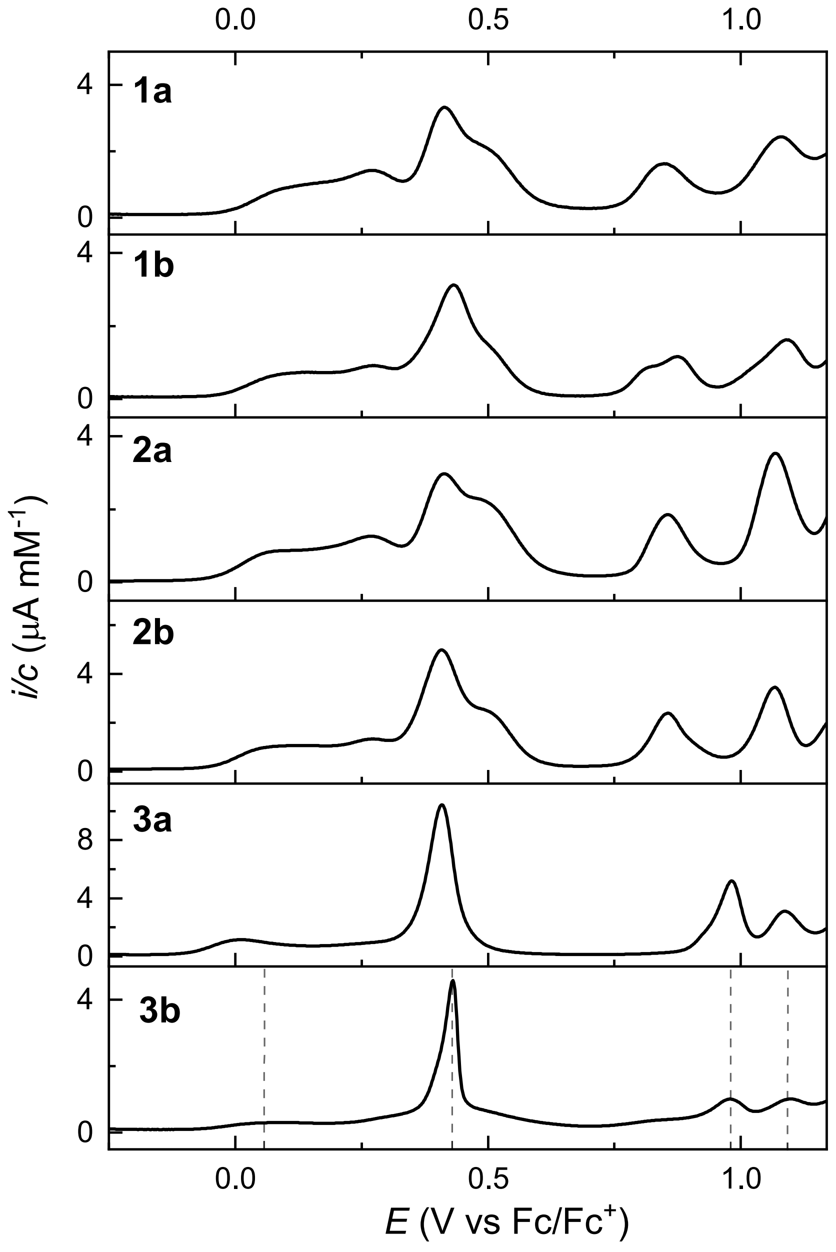

Figure S62. Differential pulse voltammograms of compounds **1**-**3**. The voltammograms were recorded in CH_2Cl_2 using *n*-Bu_4NPF_6 (0.1M) as supporting electrolyte with a scan rate of 0.2 V/s. The compounds were measured at concentrations (from the top): 0.25 mM (**1a**), 0.25 mM (**1b**), 0.50 mM (**2a**), 0.50 mM (**2b**), 0.25 mM (**3a**). Due to solubility issues for **3b** it was not possible to determine the concentration.

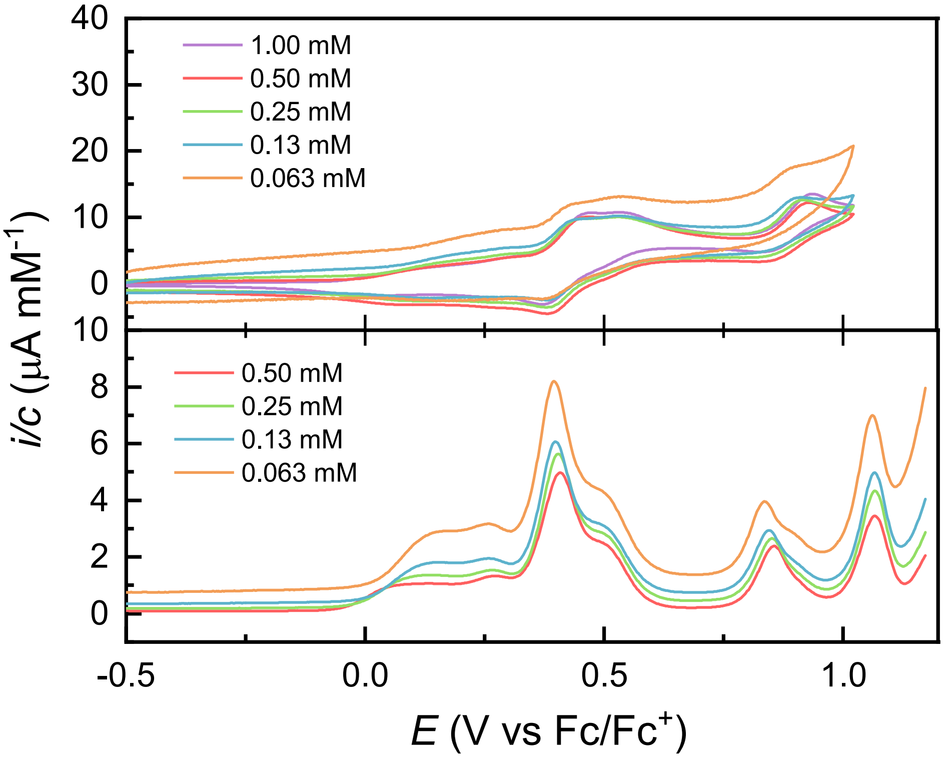

Figure S63. Cyclic voltammograms (top) and differential pulse voltammograms (bottom) of compound **2b** measured at different concentrations in CH_2Cl_2 using *n*-Bu_4NPF_6 (0.1 M) as supporting electrolyte. The CV was recorded with a scan rate of 0.1 V/s and the DPV was recorded with a scan rate of 0.2 V/s.

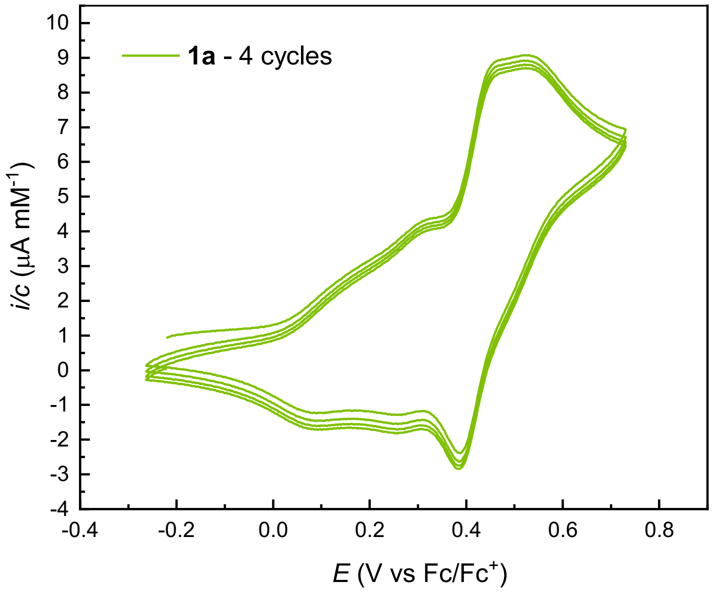

Figure S64. Cyclic voltammogram of compound **1a** recorded over 4 cycles in CH_2Cl_2 using *n*-Bu_4NPF_6 (0.1M) as supporting electrolyte at a scan rate of 0.1 V/s.

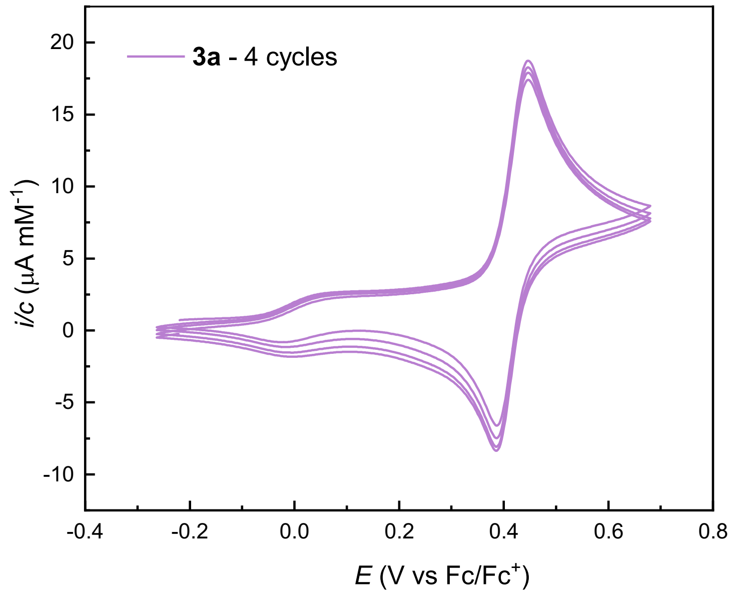

Figure S65. Cyclic voltammogram of compound **3a** recorded over 4 cycles in CH_2Cl_2 using *n*-Bu_4NPF_6 (0.1M) as supporting electrolyte at a scan rate of 0.1 V/s.

Chemical Oxidation

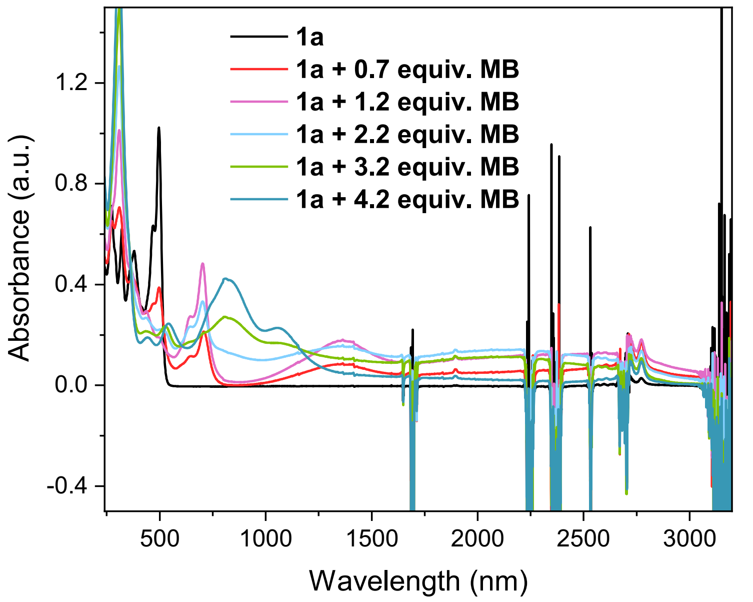

Figure S66. UV-Vis-NIR absorption spectra of **1a** with addition of different equivalents of tris(4-bromophenyl)ammoniumyl hexachloroantimonate (‘magic blue", **MB**), recorded in CH_2Cl_2 at room temperature on 0.008 mM solutions.

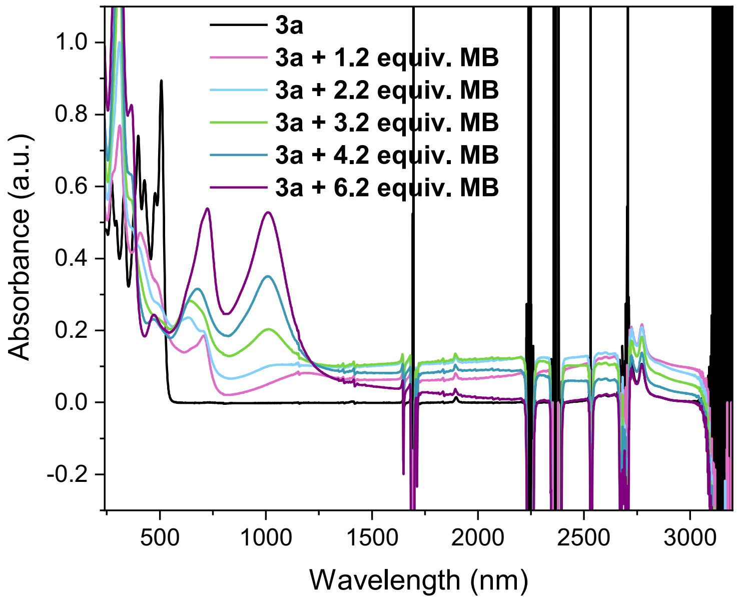

Figure S67. UV-Vis-NIR absorption spectra of **3a** with addition of different equivalents of **MB**, recorded in CH_2Cl_2 at room temperature on 0.008 mM solutions.

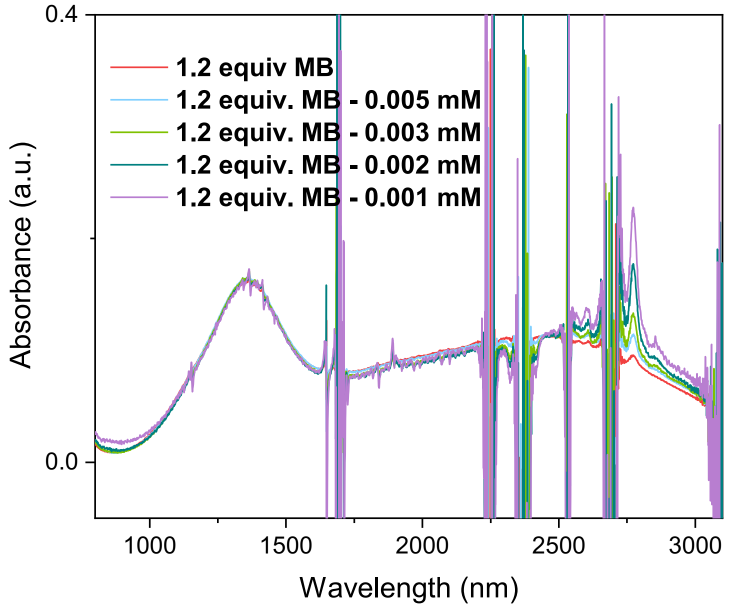

Figure S68. NIR absorption spectra of **1a** + 1.2 equivalents of **MB** recorded at different concentrations of **1a** in CH_2Cl_2 at room temperature.

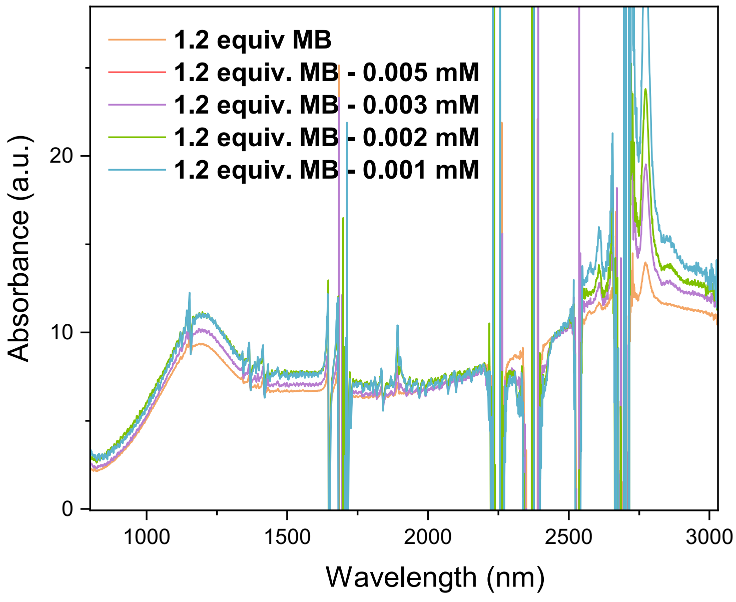

Figure S69. NIR absorption spectra of **3a** + 1.2 equivalents of **MB** recorded at different concentrations of **3a** in CH_2Cl_2 at room temperature.

ESR Measurements

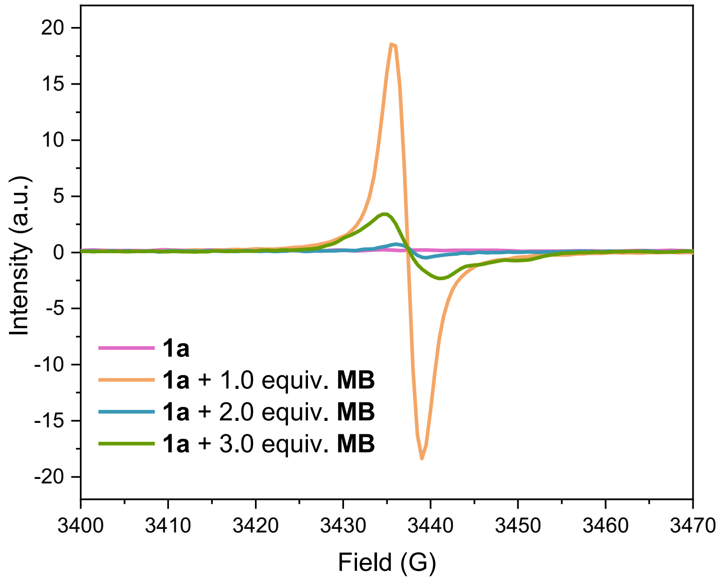

Figure S70. ESR spectra of **1a** with addition of different equivalents of **MB** recorded in CH_2Cl_2 on 0.1 mM concentrations at room temperature. Two small bumps are observed around 3445 G and 3450 G in the spectrum of **1a** + 3.0 equiv. MB (green line) due to slight over titration with **MB**.

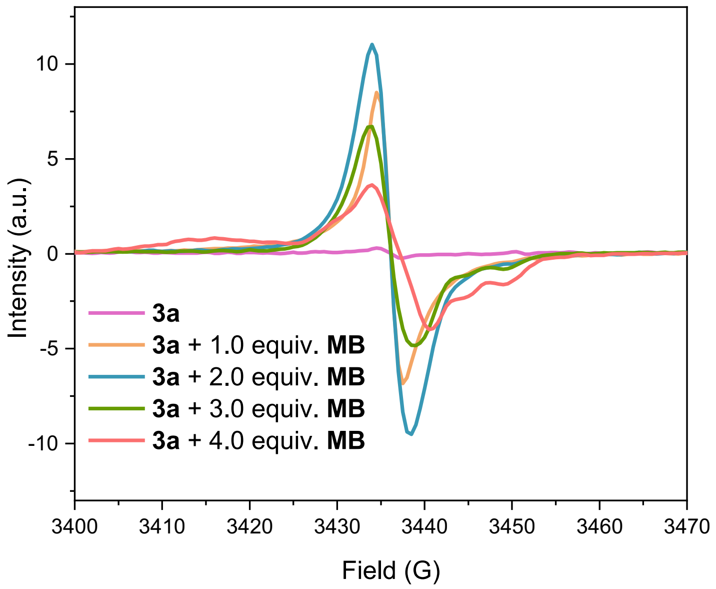

Figure S71. ESR spectra of **3a** with addition of different equivalents of **MB** recorded in CH_2Cl_2 on 0.1 mM concentrations at room temperature. A bump at 3415 G and two bumps at 3445 G and 3450 G are observed in the spectrum of **3a** + 4.0 equiv. MB (red line) due to slight over titration with **MB**.

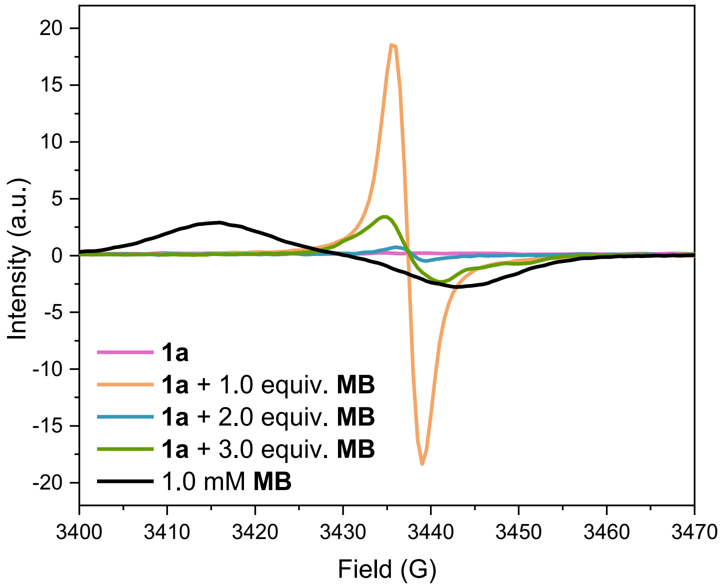

Figure S72. ESR spectra of **1a** with overlay of ESR spectrum of **MB** (1.0 mM) to recognize over titration of **MB** for the spectrum of **1a** + 3.0 equiv. MB (green line). All measurements were performed in CH_2Cl_2 at room temperature.

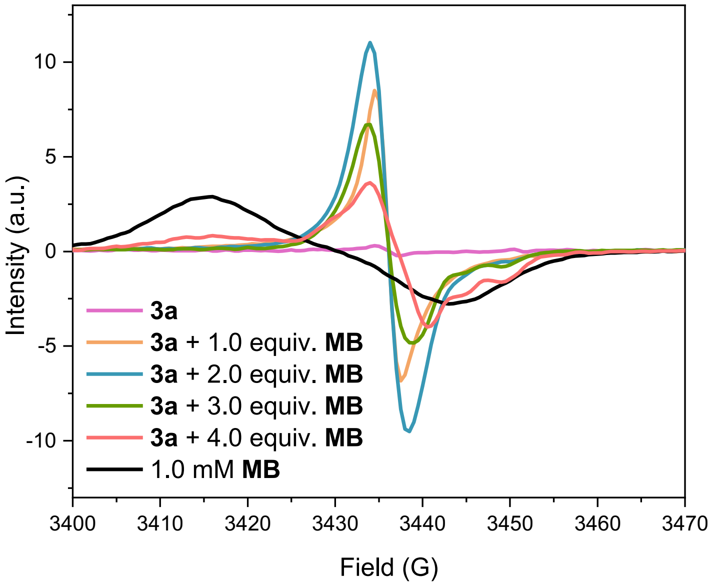

Figure S73. ESR spectra of **3a** with overlay of ESR spectrum of **MB** (1.0 mM) to recognize over titration of **MB** for the spectrum of **3a** + 4.0 equiv. MB (red line). All measurements were performed in CH_2Cl_2 at room temperature.

Crystallography

Crystallographic data for compound **1b** has been deposited to the Cambridge Crystallographic Data Centre. CCDC 2501210 contains the supplementary crystallographic data of compound **1b**, which can be obtained free of charge from The Cambridge Crystallographic Data Centre via their website. It was proven to be a challenge to grow crystals of **1b** suitable for single crystal X-ray diffraction due to its nature of nucleation forming thin needles starting from the nucleation point and growing into each other. In addition to that, data reduction for data of other needles which resulted in some diffraction also all resulted in the monoclinic space group of $P2_1/c$ with a β angle very close to 90°. This could suggest orthorhombic symmetry, but the best data reduction and model was achieved in the monoclinic space group $P2_1/c$ indicating this is a special case of β being close to 90° in a monoclinic crystal. The best diffracting needle gave the data deposited to CCDC which is reported in this paper.

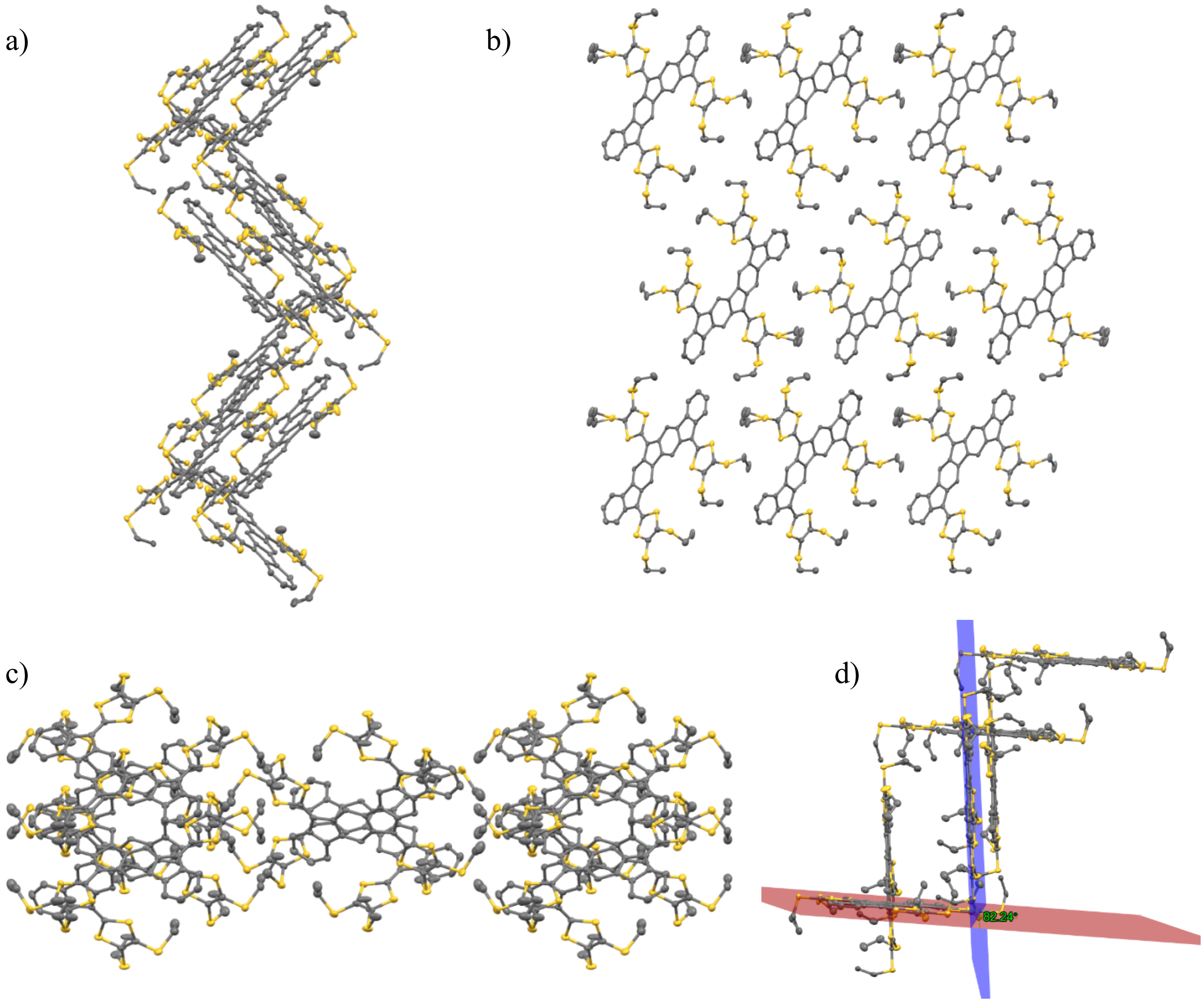

Figure S74. Crystal packing of compound **1b** seen from a) the *a* axis, b) *b* axis c) and *c* axis. d) Molecular structure of **1b** with planes defined as a mean from the atoms of the core and DTF units (excluding the SEt chains). The angle between the two planes is 82.24°. Hydrogen atoms removed for clarity.

Table S4. Crystal data and structure refinement for mo_d8v7047 (compound **1b**)

CCDC number	2501210
Empirical formula	$C_{48}H_{42}S_{12}$
Formula weight	1003.53
Temperature [K]	100.00
Crystal system	monoclinic
Space group (number)	$P2_1/c$ (14)
a [Å]	32.143(4)
b [Å]	5.1980(5)
c [Å]	27.022(3)
α [°]	90
β [°]	90.100(4)
γ [°]	90
Volume [Å^3]	4514.9(9)
Z	4
ρ_{calc} [gcm^{-3}]	1.476
μ [mm^{-1}]	0.617
F(000)	2088
Crystal size [mm^3]	0.065×0.113×0.676
Crystal colour	clear orange
Crystal shape	needle
Radiation	MoK_α (λ=0.71073 Å)
2θ range [°]	3.94 to 50.00 (0.84 Å)
Index ranges	$-38 \le h \le 38$ $-6 \le k \le 6$ $-32 \le l \le 32$
Reflections collected	70740
Independent reflections	7945 R_{int} = 0.1609 R_{sigma} = 0.0736
Completeness to θ = 25.000°	99.9 %
Data / Restraints / Parameters	7945 / 37 / 566
Absorption correction T_{min}/T_{max} (method)	0.6757 / 0.7456 (multi-scan)
Goodness-of-fit on F^2	1.032
Final R indexes [I≥2σ(I)]	R_1 = 0.0594 wR_2 = 0.1356
Final R indexes [all data]	R_1 = 0.1103 wR_2 = 0.1521
Largest peak/hole [eÅ^{-3}]	0.75/−0.50

Table S5. Atomic coordinates and U_{eq} [Å^2] for mo_d8v7047

Atom	*x*	*y*	*z*	U_{eq}
S4	0.76035(5)	−0.6734(2)	0.14680(5)	0.0356(3)
S2	0.81608(4)	−0.2867(2)	0.19554(4)	0.0276(3)
S10	0.62915(4)	0.3057(2)	0.37548(5)	0.0279(3)
S12	0.57551(4)	−0.0957(2)	0.33040(5)	0.0331(3)
S3	0.68801(5)	−0.4776(2)	0.22919(5)	0.0387(4)
S1	0.75144(4)	−0.0868(2)	0.26037(4)	0.0263(3)
S5	0.83029(4)	1.3084(2)	0.46999(4)	0.0248(3)
S7	0.88681(4)	1.6801(2)	0.52196(5)	0.0332(3)
S6	0.89651(4)	1.0792(2)	0.41123(5)	0.0281(3)
S8	0.96279(5)	1.4246(3)	0.45509(7)	0.0506(4)
S9	0.56496(4)	0.5029(2)	0.44180(5)	0.0309(3)
S11	0.50200(4)	0.1059(2)	0.41246(5)	0.0362(3)
C32	0.7529(2)	−0.2837(9)	0.07571(19)	0.0536(18)
H32A	0.764662	−0.208831	0.045543	0.080
H32B	0.753908	−0.156555	0.102465	0.080
H32C	0.723903	−0.333395	0.069595	0.080
C31	0.7774(2)	−0.5153(10)	0.09019(18)	0.0435(15)
H31A	0.806883	−0.463922	0.094284	0.052
H31B	0.776140	−0.641452	0.062778	0.052
C29	0.76658(16)	−0.4251(8)	0.18958(17)	0.0270(11)
C28	0.80317(15)	−0.0608(8)	0.24151(16)	0.0208(10)
C21	0.83099(14)	0.1094(8)	0.26116(15)	0.0195(10)
C22	0.82198(14)	0.3052(8)	0.29922(15)	0.0191(10)
C23	0.78588(14)	0.3699(8)	0.32496(15)	0.0192(10)
H23	0.760586	0.279818	0.319409	0.023
C24	0.78800(14)	0.5695(8)	0.35886(15)	0.0175(10)
C25	0.75496(14)	0.6763(8)	0.38928(15)	0.0172(9)
C26	0.71365(14)	0.6035(8)	0.39300(15)	0.0182(10)
H26	0.703008	0.466439	0.373460	0.022
C27	0.68801(14)	0.7347(8)	0.42590(15)	0.0193(10)
C1	0.64434(15)	0.7035(8)	0.44011(16)	0.0228(10)
C42	0.61657(14)	0.5301(8)	0.42138(16)	0.0211(10)
C44	0.58034(15)	0.1553(9)	0.37302(18)	0.0283(11)
C47	0.5455(2)	0.0582(11)	0.2816(2)	0.0494(16)
H47A	0.539499	−0.069308	0.255329	0.059
H47B	0.518531	0.116712	0.295339	0.059
C48	0.5679(2)	0.2863(10)	0.2591(2)	0.0486(16)
H48A	0.595150	0.231246	0.246850	0.073
H48B	0.571591	0.420054	0.284258	0.073
H48C	0.551396	0.355055	0.231548	0.073
C34	0.6530(2)	−0.1146(12)	0.1692(2)	0.0526(16)
H34A	0.681558	−0.077070	0.158698	0.079
H34B	0.636110	0.041682	0.166535	0.079
H34C	0.641231	−0.248925	0.147964	0.079
C33	0.65332(18)	−0.2049(10)	0.2216(2)	0.0450(15)
H33A	0.624790	−0.254938	0.231407	0.054
H33B	0.662457	−0.062824	0.243449	0.054
C30	0.73716(16)	−0.3391(8)	0.22057(17)	0.0282(11)
C20	0.87510(15)	0.1352(8)	0.24882(16)	0.0228(10)
C19	0.90142(16)	−0.0014(9)	0.21755(17)	0.0292(12)
H19	0.891110	−0.143173	0.199043	0.035

C18	0.94276(17)	0.0708(10)	0.21362(19)	0.0367(13)
H18	0.960652	−0.022804	0.192241	0.044
C17	0.95845(17)	0.2756(10)	0.24010(18)	0.0340(12)
H17	0.986764	0.323897	0.236254	0.041
C16	0.93303(15)	0.4122(9)	0.27244(17)	0.0286(11)
H16	0.943723	0.552621	0.291040	0.034
C15	0.89208(15)	0.3391(8)	0.27683(16)	0.0237(11)
C14	0.85915(14)	0.4456(8)	0.30851(15)	0.0203(10)
C8	0.70502(14)	0.9399(8)	0.45409(15)	0.0198(10)
C9	0.74609(14)	1.0154(8)	0.44984(15)	0.0198(10)
H9	0.756500	1.156259	0.468530	0.024
C10	0.77181(14)	0.8812(8)	0.41768(16)	0.0193(10)
C11	0.81602(14)	0.9042(8)	0.40594(15)	0.0195(10)
C12	0.82479(14)	0.7072(8)	0.36821(15)	0.0196(10)
C13	0.86108(14)	0.6447(8)	0.34226(15)	0.0202(10)
H13	0.886234	0.736485	0.347693	0.024
C35	0.84374(14)	1.0718(8)	0.42642(16)	0.0187(10)
C36	0.88063(16)	1.4284(8)	0.47968(17)	0.0273(11)
C38A	0.8741(4)	1.5217(16)	0.5793(3)	0.049(2)
H38A	0.875853	1.647565	0.606720	0.058
H38B	0.845136	1.457747	0.577648	0.058
C39B	0.8774(7)	1.292(4)	0.5952(8)	0.047(4)
H39A	0.878771	1.155091	0.570181	0.070
H39B	0.848460	1.345876	0.599452	0.070
H39C	0.888291	1.226670	0.626677	0.070
C37	0.91112(16)	1.3220(9)	0.45265(18)	0.0301(12)
C40	0.99234(17)	1.1418(10)	0.4392(2)	0.0395(13)
H40A	1.019304	1.150278	0.456880	0.047
H40B	0.977248	0.989634	0.451932	0.047
C41	1.0011(2)	1.0984(14)	0.3856(2)	0.0619(19)
H41A	0.975214	1.051394	0.368601	0.093
H41B	1.021349	0.958913	0.382088	0.093
H41C	1.012326	1.256247	0.371074	0.093
C7	0.67257(15)	1.0380(8)	0.48698(16)	0.0225(10)
C6	0.67401(16)	1.2349(9)	0.52164(17)	0.0271(11)
H6	0.698800	1.331489	0.526311	0.033
C5	0.63901(16)	1.2884(9)	0.54920(18)	0.0314(12)
H5	0.639483	1.422628	0.573057	0.038
C4	0.60302(17)	1.1451(9)	0.54189(19)	0.0346(13)
H4	0.579179	1.181341	0.561411	0.042
C3	0.60101(16)	0.9514(9)	0.50704(18)	0.0293(12)
H3	0.575951	0.857541	0.502358	0.035
C2	0.63592(15)	0.8950(8)	0.47889(16)	0.0232(11)
C43	0.55105(15)	0.2440(9)	0.40357(18)	0.0291(12)
C45	0.46865(17)	0.3866(10)	0.4063(2)	0.0385(13)
H45A	0.439985	0.339155	0.416082	0.046
H45B	0.478468	0.521701	0.429395	0.046
C46	0.46788(18)	0.4915(10)	0.35597(19)	0.0403(13)
H46A	0.496195	0.537800	0.345949	0.060
H46B	0.450176	0.644973	0.355118	0.060
H46C	0.456746	0.361950	0.333171	0.060
C38B	0.9024(7)	1.511(4)	0.5790(7)	0.050(3)
H38C	0.931337	1.449972	0.574506	0.061
H38D	0.902751	1.638094	0.606204	0.061
C39A	0.9025(4)	1.3026(19)	0.5898(4)	0.058(3)

H39D	0.901305	1.179058	0.562473	0.086
H39E	0.893960	1.217721	0.620558	0.086
H39F	0.931060	1.366532	0.593424	0.086

U_{eq} is defined as 1/3 of the trace of the orthogonalized U_{ij} tensor.

Table S6. Anisotropic displacement parameters [Å^2] for mo_d8v7047. The anisotropic displacement factor exponent takes the form: $-2\pi^2[\ h^2(a^*)^2U_{11} + k^2(b^*)^2U_{22} + \ldots + 2hka^*b^*U_{12}\ ]$

Atom	U_{11}	U_{22}	U_{33}	U_{23}	U_{13}	U_{12}
S4	0.0599(10)	0.0204(6)	0.0263(7)	−0.0058(5)	−0.0075(6)	−0.0029(6)
S2	0.0399(8)	0.0220(6)	0.0207(6)	−0.0056(5)	−0.0033(5)	0.0018(5)
S10	0.0283(7)	0.0247(6)	0.0307(7)	−0.0055(5)	−0.0001(5)	−0.0014(5)
S12	0.0368(8)	0.0266(6)	0.0358(8)	−0.0038(5)	−0.0010(6)	−0.0026(6)
S3	0.0406(9)	0.0269(7)	0.0485(9)	−0.0076(6)	−0.0068(7)	−0.0052(6)
S1	0.0330(8)	0.0211(6)	0.0248(6)	−0.0046(5)	−0.0059(5)	−0.0012(5)
S5	0.0293(7)	0.0219(6)	0.0233(6)	−0.0058(5)	−0.0063(5)	−0.0007(5)
S7	0.0441(9)	0.0223(6)	0.0333(7)	−0.0087(5)	−0.0109(6)	−0.0033(6)
S6	0.0254(7)	0.0256(6)	0.0332(7)	−0.0089(5)	−0.0041(5)	−0.0022(5)
S8	0.0300(9)	0.0362(8)	0.0855(12)	−0.0230(8)	−0.0087(8)	−0.0077(6)
S9	0.0298(8)	0.0289(6)	0.0341(7)	−0.0056(6)	0.0031(6)	−0.0004(5)
S11	0.0300(8)	0.0325(7)	0.0462(8)	−0.0024(6)	0.0021(6)	−0.0030(6)
C32	0.116(6)	0.021(3)	0.024(3)	−0.003(2)	−0.019(3)	0.004(3)
C31	0.076(5)	0.034(3)	0.021(3)	−0.011(2)	−0.005(3)	−0.004(3)
C29	0.045(3)	0.015(2)	0.020(2)	−0.0017(19)	−0.009(2)	−0.003(2)
C28	0.028(3)	0.019(2)	0.015(2)	−0.0022(18)	0.001(2)	0.0017(19)
C21	0.028(3)	0.019(2)	0.012(2)	0.0012(18)	−0.0057(19)	0.0032(19)
C22	0.030(3)	0.014(2)	0.013(2)	0.0021(17)	−0.006(2)	0.0020(19)
C23	0.021(3)	0.020(2)	0.017(2)	0.0006(18)	−0.0048(19)	0.0009(19)
C24	0.021(3)	0.018(2)	0.014(2)	0.0021(17)	−0.0042(18)	0.0020(18)
C25	0.022(3)	0.018(2)	0.012(2)	−0.0002(17)	−0.0048(18)	0.0036(18)
C26	0.026(3)	0.016(2)	0.012(2)	−0.0005(17)	−0.0016(19)	−0.0016(18)
C27	0.022(3)	0.019(2)	0.016(2)	0.0012(18)	−0.0020(19)	0.0019(18)
C1	0.031(3)	0.019(2)	0.018(2)	0.0025(18)	−0.002(2)	0.005(2)
C42	0.023(3)	0.019(2)	0.022(2)	−0.0033(19)	0.001(2)	0.0029(19)
C44	0.029(3)	0.024(2)	0.031(3)	−0.001(2)	0.002(2)	−0.004(2)
C47	0.051(4)	0.055(4)	0.042(4)	−0.011(3)	−0.008(3)	0.003(3)
C48	0.087(5)	0.031(3)	0.027(3)	−0.001(2)	−0.008(3)	−0.001(3)
C34	0.046(4)	0.063(4)	0.048(4)	0.005(3)	−0.009(3)	−0.005(3)
C33	0.039(4)	0.038(3)	0.058(4)	−0.003(3)	0.005(3)	0.001(3)
C30	0.039(3)	0.021(2)	0.025(3)	−0.002(2)	−0.007(2)	−0.001(2)
C20	0.030(3)	0.022(2)	0.017(2)	0.0038(19)	−0.002(2)	0.002(2)
C19	0.034(3)	0.030(3)	0.023(3)	−0.011(2)	−0.002(2)	0.007(2)
C18	0.032(3)	0.047(3)	0.031(3)	−0.009(2)	0.000(2)	0.016(3)
C17	0.026(3)	0.046(3)	0.030(3)	−0.008(2)	−0.002(2)	0.006(2)
C16	0.028(3)	0.032(3)	0.026(3)	−0.004(2)	−0.004(2)	0.001(2)
C15	0.029(3)	0.021(2)	0.021(2)	−0.0025(19)	−0.004(2)	0.005(2)
C14	0.026(3)	0.021(2)	0.014(2)	0.0010(18)	−0.0037(19)	0.0033(19)
C8	0.024(3)	0.019(2)	0.016(2)	0.0002(18)	−0.0046(19)	0.0027(19)
C9	0.031(3)	0.014(2)	0.014(2)	−0.0037(17)	−0.003(2)	0.0016(19)
C10	0.022(3)	0.017(2)	0.018(2)	0.0018(18)	−0.0024(19)	0.0009(18)
C11	0.024(3)	0.018(2)	0.016(2)	−0.0007(18)	−0.0030(19)	0.0037(19)
C12	0.031(3)	0.016(2)	0.012(2)	0.0011(17)	−0.0058(19)	0.0025(19)
C13	0.022(3)	0.023(2)	0.016(2)	0.0011(18)	−0.0052(19)	−0.0047(19)
C35	0.020(3)	0.016(2)	0.020(2)	−0.0002(18)	−0.0045(19)	0.0002(18)
C36	0.036(3)	0.022(2)	0.024(3)	−0.007(2)	−0.007(2)	−0.003(2)
C38A	0.083(7)	0.033(3)	0.030(4)	−0.007(3)	−0.017(6)	−0.005(5)
C39B	0.081(9)	0.030(5)	0.031(5)	−0.007(5)	−0.013(8)	−0.008(7)

C37	0.030(3)	0.025(2)	0.035(3)	−0.005(2)	−0.011(2)	−0.006(2)
C40	0.033(3)	0.041(3)	0.045(3)	−0.001(3)	−0.005(3)	−0.003(2)
C41	0.057(5)	0.084(5)	0.044(4)	−0.006(4)	0.012(3)	−0.016(4)
C7	0.029(3)	0.023(2)	0.016(2)	0.0019(19)	−0.002(2)	0.004(2)
C6	0.027(3)	0.028(3)	0.026(3)	−0.004(2)	−0.006(2)	0.001(2)
C5	0.039(3)	0.032(3)	0.023(3)	−0.011(2)	0.003(2)	0.005(2)
C4	0.037(3)	0.035(3)	0.032(3)	−0.006(2)	0.009(2)	0.007(2)
C3	0.030(3)	0.027(3)	0.030(3)	0.000(2)	0.005(2)	0.003(2)
C2	0.034(3)	0.018(2)	0.018(2)	0.0011(18)	0.000(2)	0.004(2)
C43	0.026(3)	0.028(3)	0.033(3)	−0.001(2)	−0.001(2)	−0.002(2)
C45	0.027(3)	0.040(3)	0.049(4)	−0.005(3)	0.002(3)	0.002(2)
C46	0.038(3)	0.047(3)	0.035(3)	−0.008(3)	−0.004(3)	0.001(3)
C38B	0.086(8)	0.035(4)	0.031(4)	−0.004(4)	−0.019(7)	−0.008(6)
C39A	0.105(8)	0.033(4)	0.035(4)	−0.008(3)	−0.027(6)	−0.001(6)

Table S7. Bond lengths and angles for mo_d8v7047

**Atom–Atom**	**Length [Å]**
S4–C31	1.822(5)
S4–C29	1.744(4)
S2–C29	1.753(5)
S2–C28	1.760(4)
S10–C42	1.750(4)
S10–C44	1.754(5)
S12–C44	1.747(5)
S12–C47	1.819(6)
S3–C33	1.815(6)
S3–C30	1.752(5)
S1–C28	1.745(5)
S1–C30	1.756(5)
S5–C35	1.757(4)
S5–C36	1.753(5)
S7–C36	1.748(4)
S7–C38A	1.802(9)
S7–C38B	1.84(2)
S6–C35	1.746(5)
S6–C37	1.751(5)
S8–C37	1.745(5)
S8–C40	1.803(5)
S9–C42	1.755(5)
S9–C43	1.754(5)
S11–C43	1.749(5)
S11–C45	1.818(5)
C32–H32A	0.9800
C32–H32B	0.9800
C32–H32C	0.9800
C32–C31	1.491(8)
C31–H31A	0.9900
C31–H31B	0.9900
C29–C30	1.341(7)
C28–C21	1.365(6)
C21–C22	1.476(6)
C21–C20	1.463(6)
C22–C23	1.395(6)
C22–C14	1.422(6)
C23–H23	0.9500
C23–C24	1.385(6)
C24–C25	1.454(6)
C24–C12	1.405(6)
C25–C26	1.385(6)
C25–C10	1.420(6)
C26–H26	0.9500
C26–C27	1.392(6)
C27–C1	1.465(6)
C27–C8	1.419(6)
C1–C42	1.365(6)
C1–C2	1.471(6)
C44–C43	1.335(7)
C47–H47A	0.9900
C47–H47B	0.9900
C47–C48	1.516(8)
C48–H48A	0.9800
C48–H48B	0.9800
C48–H48C	0.9800
C34–H34A	0.9800
C34–H34B	0.9800
C34–H34C	0.9800
C34–C33	1.491(8)
C33–H33A	0.9900
C33–H33B	0.9900
C20–C19	1.392(6)
C20–C15	1.412(6)
C19–H19	0.9500
C19–C18	1.385(7)
C18–H18	0.9500
C18–C17	1.378(7)
C17–H17	0.9500
C17–C16	1.392(7)
C16–H16	0.9500
C16–C15	1.375(7)
C15–C14	1.471(6)
C14–C13	1.381(6)
C8–C9	1.382(6)
C8–C7	1.463(6)
C9–H9	0.9500
C9–C10	1.389(6)
C10–C11	1.461(6)
C11–C12	1.473(6)

C11–C35	1.363(6)
C12–C13	1.400(6)
C13–H13	0.9500
C36–C37	1.343(7)
C38A–H38A	0.9900
C38A–H38B	0.9900
C38A–C39A	1.487(11)
C39B–H39A	0.9800
C39B–H39B	0.9800
C39B–H39C	0.9800
C39B–C38B	1.461(17)
C40–H40A	0.9900
C40–H40B	0.9900
C40–C41	1.491(7)
C41–H41A	0.9800
C41–H41B	0.9800
C41–H41C	0.9800
C7–C6	1.388(6)
C7–C2	1.410(7)
C6–H6	0.9500
C6–C5	1.379(7)
C5–H5	0.9500
C5–C4	1.390(7)
C4–H4	0.9500
C4–C3	1.380(7)
C3–H3	0.9500
C3–C2	1.388(6)
C45–H45A	0.9900
C45–H45B	0.9900
C45–C46	1.467(7)
C46–H46A	0.9800
C46–H46B	0.9800
C46–H46C	0.9800
C38B–H38C	0.9900
C38B–H38D	0.9900
C39A–H39D	0.9800
C39A–H39E	0.9800
C39A–H39F	0.9800
**Atom–Atom–Atom**	**Angle [°]**
C29–S4–C31	100.9(2)
C29–S2–C28	97.1(2)
C42–S10–C44	96.7(2)
C44–S12–C47	101.3(2)
C30–S3–C33	102.6(2)
C28–S1–C30	97.3(2)
C36–S5–C35	97.0(2)
C36–S7–C38A	101.2(3)
C36–S7–C38B	102.7(6)
C35–S6–C37	97.2(2)
C37–S8–C40	104.1(2)
C43–S9–C42	96.7(2)
C43–S11–C45	100.9(2)
H32A–C32–H32B	109.5
H32A–C32–H32C	109.5
H32B–C32–H32C	109.5
C31–C32–H32A	109.5
C31–C32–H32B	109.5

C31–C32–H32C	109.5
S4–C31–H31A	108.5
S4–C31–H31B	108.5
C32–C31–S4	115.1(4)
C32–C31–H31A	108.5
C32–C31–H31B	108.5
H31A–C31–H31B	107.5
S4–C29–S2	117.9(3)
C30–C29–S4	125.5(4)
C30–C29–S2	116.5(4)
S1–C28–S2	112.3(2)
C21–C28–S2	123.5(4)
C21–C28–S1	124.1(3)
C28–C21–C22	126.1(4)
C28–C21–C20	127.2(4)
C20–C21–C22	106.7(4)
C23–C22–C21	132.7(4)
C23–C22–C14	119.2(4)
C14–C22–C21	108.1(4)
C22–C23–H23	121.0
C24–C23–C22	118.0(4)
C24–C23–H23	121.0
C23–C24–C25	128.7(4)
C23–C24–C12	122.7(4)
C12–C24–C25	108.6(4)
C26–C25–C24	129.6(4)
C26–C25–C10	122.1(4)
C10–C25–C24	108.3(4)
C25–C26–H26	120.6
C25–C26–C27	118.8(4)
C27–C26–H26	120.6
C26–C27–C1	133.0(4)
C26–C27–C8	118.9(4)
C8–C27–C1	108.1(4)
C27–C1–C2	106.8(4)
C42–C1–C27	127.0(4)
C42–C1–C2	126.2(4)
S10–C42–S9	112.9(2)
C1–C42–S10	123.4(4)
C1–C42–S9	123.7(3)
S12–C44–S10	115.9(3)
C43–C44–S10	117.0(4)
C43–C44–S12	127.1(4)
S12–C47–H47A	109.1
S12–C47–H47B	109.1
H47A–C47–H47B	107.8
C48–C47–S12	112.5(4)
C48–C47–H47A	109.1
C48–C47–H47B	109.1
C47–C48–H48A	109.5
C47–C48–H48B	109.5
C47–C48–H48C	109.5
H48A–C48–H48B	109.5
H48A–C48–H48C	109.5
H48B–C48–H48C	109.5
H34A–C34–H34B	109.5
H34A–C34–H34C	109.5
H34B–C34–H34C	109.5

C33–C34–H34A	109.5
C33–C34–H34B	109.5
C33–C34–H34C	109.5
S3–C33–H33A	109.5
S3–C33–H33B	109.5
C34–C33–S3	110.9(4)
C34–C33–H33A	109.5
C34–C33–H33B	109.5
H33A–C33–H33B	108.1
S3–C30–S1	117.4(3)
C29–C30–S3	125.7(4)
C29–C30–S1	116.6(4)
C19–C20–C21	133.0(4)
C19–C20–C15	118.3(5)
C15–C20–C21	108.7(4)
C20–C19–H19	120.2
C18–C19–C20	119.5(5)
C18–C19–H19	120.2
C19–C18–H18	119.3
C17–C18–C19	121.3(5)
C17–C18–H18	119.3
C18–C17–H17	119.8
C18–C17–C16	120.4(5)
C16–C17–H17	119.8
C17–C16–H16	120.8
C15–C16–C17	118.4(5)
C15–C16–H16	120.8
C20–C15–C14	108.5(4)
C16–C15–C20	122.0(4)
C16–C15–C14	129.5(4)
C22–C14–C15	108.0(4)
C13–C14–C22	122.6(4)
C13–C14–C15	129.4(4)
C27–C8–C7	108.3(4)
C9–C8–C27	122.4(4)
C9–C8–C7	129.3(4)
C8–C9–H9	120.7
C8–C9–C10	118.6(4)
C10–C9–H9	120.7
C25–C10–C11	108.3(4)
C9–C10–C25	119.2(4)
C9–C10–C11	132.5(4)
C10–C11–C12	106.3(4)
C35–C11–C10	126.8(4)
C35–C11–C12	126.9(4)
C24–C12–C11	108.5(4)
C13–C12–C24	119.6(4)
C13–C12–C11	132.0(4)
C14–C13–C12	117.9(4)
C14–C13–H13	121.0
C12–C13–H13	121.0
S6–C35–S5	112.5(2)
C11–C35–S5	123.9(3)
C11–C35–S6	123.6(3)
S7–C36–S5	117.9(3)
C37–C36–S5	116.5(4)
C37–C36–S7	125.6(4)
S7–C38A–H38A	109.2
S7–C38A–H38B	109.2
H38A–C38A–H38B	107.9
C39A–C38A–S7	112.0(8)
C39A–C38A–H38A	109.2
C39A–C38A–H38B	109.2
H39A–C39B–H39B	109.5
H39A–C39B–H39C	109.5
H39B–C39B–H39C	109.5
C38B–C39B–H39A	109.5
C38B–C39B–H39B	109.5
C38B–C39B–H39C	109.5
S8–C37–S6	119.9(3)
C36–C37–S6	116.7(4)
C36–C37–S8	123.3(4)
S8–C40–H40A	108.0
S8–C40–H40B	108.0
H40A–C40–H40B	107.3
C41–C40–S8	117.0(4)
C41–C40–H40A	108.0
C41–C40–H40B	108.0
C40–C41–H41A	109.5
C40–C41–H41B	109.5
C40–C41–H41C	109.5
H41A–C41–H41B	109.5
H41A–C41–H41C	109.5
H41B–C41–H41C	109.5
C6–C7–C8	130.1(4)
C6–C7–C2	121.3(4)
C2–C7–C8	108.6(4)
C7–C6–H6	120.4
C5–C6–C7	119.1(5)
C5–C6–H6	120.4
C6–C5–H5	120.2
C6–C5–C4	119.7(4)
C4–C5–H5	120.2
C5–C4–H4	119.1
C3–C4–C5	121.7(5)
C3–C4–H4	119.1
C4–C3–H3	120.3
C4–C3–C2	119.4(5)
C2–C3–H3	120.3
C7–C2–C1	108.2(4)
C3–C2–C1	133.1(5)
C3–C2–C7	118.7(4)
S11–C43–S9	117.6(3)
C44–C43–S9	116.7(4)
C44–C43–S11	125.5(4)
S11–C45–H45A	109.0
S11–C45–H45B	109.0
H45A–C45–H45B	107.8
C46–C45–S11	113.1(4)
C46–C45–H45A	109.0
C46–C45–H45B	109.0
C45–C46–H46A	109.5
C45–C46–H46B	109.5
C45–C46–H46C	109.5
H46A–C46–H46B	109.5
H46A–C46–H46C	109.5

H46B–C46–H46C	109.5
S7–C38B–H38C	107.7
S7–C38B–H38D	107.7
C39B–C38B–S7	118.3(16)
C39B–C38B–H38C	107.7
C39B–C38B–H38D	107.7
H38C–C38B–H38D	107.1
C38A–C39A–H39D	109.5
C38A–C39A–H39E	109.5
C38A–C39A–H39F	109.5
H39D–C39A–H39E	109.5
H39D–C39A–H39F	109.5
H39E–C39A–H39F	109.5

Table S8. Torsion angles for mo_d8v7047

Atom–Atom–Atom–Atom	Torsion Angle [°]
S4–C29–C30–S3	6.8(7)
S4–C29–C30–S1	179.7(3)
S2–C29–C30–S3	−169.3(3)
S2–C29–C30–S1	3.6(5)
S2–C28–C21–C22	179.2(3)
S2–C28–C21–C20	−0.6(6)
S10–C44–C43–S9	0.8(6)
S10–C44–C43–S11	−173.1(3)
S12–C44–C43–S9	−179.1(3)
S12–C44–C43–S11	7.0(7)
S1–C28–C21–C22	−3.1(6)
S1–C28–C21–C20	177.2(3)
S5–C36–C37–S6	0.3(5)
S5–C36–C37–S8	177.2(3)
S7–C36–C37–S6	−177.8(3)
S7–C36–C37–S8	−0.9(7)
C31–S4–C29–S2	−57.2(3)
C31–S4–C29–C30	126.7(5)
C29–S4–C31–C32	−60.1(5)
C29–S2–C28–S1	2.4(3)
C29–S2–C28–C21	−179.6(4)
C28–S2–C29–S4	179.9(3)
C28–S2–C29–C30	−3.7(4)
C28–S1–C30–S3	171.9(3)
C28–S1–C30–C29	−1.6(4)
C28–C21–C22–C23	0.7(8)
C28–C21–C22–C14	−178.7(4)
C28–C21–C20–C19	−3.5(8)
C28–C21–C20–C15	178.5(4)
C21–C22–C23–C24	−179.6(4)
C21–C22–C14–C15	−0.5(5)
C21–C22–C14–C13	179.6(4)
C21–C20–C19–C18	−179.9(5)
C21–C20–C15–C16	−178.8(4)
C21–C20–C15–C14	1.0(5)
C22–C21–C20–C19	176.8(5)
C22–C21–C20–C15	−1.3(5)
C22–C23–C24–C25	178.6(4)
C22–C23–C24–C12	−0.1(6)
C22–C14–C13–C12	0.4(6)
C23–C22–C14–C15	180.0(4)
C23–C22–C14–C13	0.1(6)
C23–C24–C25–C26	1.6(7)
C23–C24–C25–C10	−179.1(4)
C23–C24–C12–C11	180.0(4)
C23–C24–C12–C13	0.6(6)
C24–C25–C26–C27	178.8(4)
C24–C25–C10–C9	179.9(4)
C24–C25–C10–C11	−0.7(5)
C24–C12–C13–C14	−0.7(6)
C25–C24–C12–C11	1.0(5)
C25–C24–C12–C13	−178.3(4)
C25–C26–C27–C1	−177.6(4)
C25–C26–C27–C8	0.6(6)
C25–C10–C11–C12	1.3(5)
C25–C10–C11–C35	−177.7(4)
C26–C25–C10–C9	−0.8(6)
C26–C25–C10–C11	178.7(4)
C26–C27–C1–C42	−2.7(8)
C26–C27–C1–C2	177.1(4)
C26–C27–C8–C9	0.5(6)
C26–C27–C8–C7	−177.7(4)
C27–C1–C42–S10	0.3(7)
C27–C1–C42–S9	178.6(3)
C27–C1–C2–C7	1.0(5)
C27–C1–C2–C3	−177.9(5)
C27–C8–C9–C10	−1.6(6)
C27–C8–C7–C6	179.5(4)
C27–C8–C7–C2	−0.2(5)
C1–C27–C8–C9	179.0(4)
C1–C27–C8–C7	0.9(5)
C42–S10–C44–S12	179.6(3)
C42–S10–C44–C43	−0.4(4)
C42–S9–C43–S11	173.6(3)
C42–S9–C43–C44	−0.8(4)
C42–C1–C2–C7	−179.2(4)
C42–C1–C2–C3	1.8(8)
C44–S10–C42–S9	−0.2(3)
C44–S10–C42–C1	178.3(4)
C44–S12–C47–C48	61.2(4)
C47–S12–C44–S10	−104.4(3)
C47–S12–C44–C43	75.5(5)
C33–S3–C30–S1	58.4(3)
C33–S3–C30–C29	−128.8(5)
C30–S3–C33–C34	68.9(5)
C30–S1–C28–S2	−0.9(3)
C30–S1–C28–C21	−178.9(4)
C20–C21–C22–C23	−179.5(4)
C20–C21–C22–C14	1.1(5)
C20–C19–C18–C17	0.0(8)
C20–C15–C14–C22	−0.3(5)
C20–C15–C14–C13	179.6(4)
C19–C20–C15–C16	2.8(7)
C19–C20–C15–C14	−177.4(4)
C19–C18–C17–C16	1.4(8)
C18–C17–C16–C15	−0.6(7)
C17–C16–C15–C20	−1.5(7)
C17–C16–C15–C14	178.7(4)
C16–C15–C14–C22	179.5(5)
C16–C15–C14–C13	−0.7(8)
C15–C20–C19–C18	−2.0(7)
C15–C14–C13–C12	−179.5(4)
C14–C22–C23–C24	−0.2(6)
C8–C27–C1–C42	179.1(4)
C8–C27–C1–C2	−1.2(5)
C8–C9–C10–C25	1.8(6)
C8–C9–C10–C11	−177.5(4)
C8–C7–C6–C5	−178.7(4)
C8–C7–C2–C1	−0.5(5)
C8–C7–C2–C3	178.6(4)
C9–C8–C7–C6	1.5(8)
C9–C8–C7–C2	−178.2(4)

C9–C10–C11–C12	−179.4(4)
C9–C10–C11–C35	1.6(8)
C10–C25–C26–C27	−0.4(6)
C10–C11–C12–C24	−1.4(5)
C10–C11–C12–C13	177.8(4)
C10–C11–C35–S5	−3.2(6)
C10–C11–C35–S6	178.1(3)
C11–C12–C13–C14	−179.9(4)
C12–C24–C25–C26	−179.5(4)
C12–C24–C25–C10	−0.2(5)
C12–C11–C35–S5	177.9(3)
C12–C11–C35–S6	−0.7(6)
C35–S5–C36–S7	−179.7(3)
C35–S5–C36–C37	2.0(4)
C35–S6–C37–S8	−179.4(3)
C35–S6–C37–C36	−2.5(4)
C35–C11–C12–C24	177.6(4)
C35–C11–C12–C13	−3.1(8)
C36–S5–C35–S6	−3.6(3)
C36–S5–C35–C11	177.7(4)
C36–S7–C38A–C39A	60.2(8)
C36–S7–C38B–C39B	−50(2)
C38A–S7–C36–S5	67.3(4)
C38A–S7–C36–C37	−114.7(6)
C37–S6–C35–S5	3.7(3)
C37–S6–C35–C11	−177.5(4)
C37–S8–C40–C41	89.1(5)
C40–S8–C37–S6	−31.0(4)
C40–S8–C37–C36	152.3(4)
C7–C8–C9–C10	176.1(4)
C7–C6–C5–C4	0.0(7)
C6–C7–C2–C1	179.7(4)
C6–C7–C2–C3	−1.1(7)
C6–C5–C4–C3	−1.0(8)
C5–C4–C3–C2	0.9(8)
C4–C3–C2–C1	179.0(5)
C4–C3–C2–C7	0.2(7)
C2–C1–C42–S10	−179.4(3)
C2–C1–C42–S9	−1.1(7)
C2–C7–C6–C5	1.0(7)
C43–S9–C42–S10	0.5(3)
C43–S9–C42–C1	−178.0(4)
C43–S11–C45–C46	67.8(4)
C45–S11–C43–S9	54.9(3)
C45–S11–C43–C44	−131.2(5)
C38B–S7–C36–S5	96.8(8)
C38B–S7–C36–C37	−85.1(9)

References

[42] X. Yang, X. Shi, N. Aratani, T. P. Gonçalves, K. W. Huang, H. Yamada, C. Chi, Q. Miao, *Chem. Sci.* **2016**, *7*, 6176–6181.

[43] P. Klein, H. J. Jötten, C. M. Aitchison, R. Clowes, E. Preis, A. I. Cooper, R. S. Sprick, U. Scherf, *Polym. Chem.* **2019**, *10*, 5200–5205.

[44] T. Yu, W. Guan, X. Wang, Y. Zhao, Q. Yang, Y. Li, H. Zhang, *New J. Chem.* **2018**, *42*, 2094–2103.

[45] X. Ren, H. Zhang, M. Song, C. Cheng, H. Zhao, Y. Wu, *Macromol. Chem. Phys.* **2019**, *220*, 1900044.

[46] J. Wang, R. Li, Z. Dong, P. Liu, G. Dong, *Nat. Chem.* **2018**, *10*, 866 – 872.

[47] J. Granhøj, V. B. R. Pedersen, P. L. Krøll, L. Broløs, M. B. Nielsen, *J. Org. Chem.* **2023**, *88*, 12853–12856.

---

[49] https://supramolecular.org

---

[53] Bruker, *SAINT*, *V8.40B*, Bruker AXS Inc., Madison, Wisconsin, USA.

[54] L. Krause, R. Herbst-Irmer, G. M. Sheldrick, D. Stalke, *J. Appl. Cryst.* **2015**, *48*, 3–10.

[55] G. M. Sheldrick, *Acta Cryst.* **2015**, *A71*, 3–8.

[56] G. M. Sheldrick, *Acta Cryst.* **2015**, *C71*, 3–8.

[57] C. R. Groom, I. J. Bruno, M. P. Lightfoot, S. C. Ward, *Acta Cryst.* **2016**, *B72*, 171–179

[58] D. Kratzert, *FinalCif*, *V152*, https://dkratzert.de/finalcif.html.
